# Global proteomic analysis of prenylated proteins in *Plasmodium falciparum* using an alkyne-modified isoprenoid analogue

**DOI:** 10.1038/srep38615

**Published:** 2016-12-07

**Authors:** Kiall F. Suazo, Chad Schaber, Charuta C. Palsuledesai, Audrey R. Odom John, Mark D. Distefano

**Affiliations:** 1 Department of Chemistry, University of Minnesota, Minneapolis, MN 55455 USA; 2 Departments of Pediatrics and of Molecular Microbiology, Washington University School of Medicine, St. Louis, MO 63110 USA

## Abstract

Severe malaria due to *Plasmodium falciparum* infection remains a serious threat to health worldwide and new therapeutic targets are highly desirable. Small molecule inhibitors of prenyl transferases, enzymes that catalyze the post-translational isoprenyl modifications of proteins, exhibit potent antimalarial activity. The antimalarial actions of prenyltransferase inhibitors indicate that protein prenylation is required for malaria parasite development. In this study, we used a chemical biology strategy to experimentally characterize the entire complement of prenylated proteins in the human malaria parasite. In contrast to the expansive mammalian and fungal prenylomes, we find that *P. falciparum* possesses a restricted set of prenylated proteins. The prenylome of *P. falciparum* is dominated by Rab GTPases, in addition to a small number of prenylated proteins that also appear to function primarily in membrane trafficking. Overall, we found robust experimental evidence for a total of only thirteen prenylated proteins in *P. falciparum*, with suggestive evidence for an additional two probable prenyltransferase substrates. Our work contributes to an increasingly complete picture of essential, post-translational hydrophobic modifications in blood-stage *P. falciparum*.

Over the past 15 years, improved efforts at controlling malaria, caused by infection with the protozoan parasite *Plasmodium falciparum*, have significantly decreased the overall number of cases and childhood deaths attributable to severe malaria^1^. However, there remain over 200 million infections and over half a million deaths due to malaria each year^2^. Access to highly effective antimalarial therapies remains a cornerstone of malaria control efforts. Unfortunately, widespread resistance to former first-line agents, such as chloroquine, and emerging resistance to newer treatments, such as the artemisinin-combination therapies, endangers control of malaria worldwide^3^,^4^.

Target-based antimalarial drug development depends on identification of essential biological processes in *P. falciparum* that are amenable to small molecule inhibition. Development of therapeutics for the developing world is hampered by a relative lack of commercial pharmaceutical interest. Therefore, one strategy has been to identify potential antimalarial target proteins whose human homologs have themselves been explored as pharmaceutical targets. These kinds of repurposing approaches thus harness the power of previous large-scale small molecule screening and development pipelines, in hopes of reducing the effort and expense of developing novel antiparasitics for resource-limited settings.

Protein prenyltransferases have emerged as one such “piggybacking” target for antimalarial drug development^5^,^6^. Protein prenylation is the C-terminal modification of cellular proteins with either a farnesyl (15-carbon) or geranylgeranyl (20-carbon) isoprenyl group. Prenyl modification of proteins is catalyzed by several classes of cellular prenyltransferase enzymes, including farnesyl transferase (FT) and geranylgeranyltransferase type I and type II (GGT-1 and GGT-2)^7^,^8^. Prenylation is typically required for the membrane association and therefore the cellular activity of prenyltransferase substrates. For example, farnesylation of the small G-protein oncogene, K-Ras, is required for the transformation of many human cancers, including lung and colon cancer^9^. For this reason, protein farnesyltransferase inhibitors have been extensively explored by the pharmaceutical industry as potential human chemotherapeutics^10^,^11^. Like most eukaryotic organisms, *P. falciparum* malaria parasites also possess protein prenyltransferase activity and have been found to incorporate both farnesyl and geranylgeranyl modifications into protein substrates^12^,^13^. Chemical inhibition of isoprenoid precursor biosynthesis in malaria parasites blocks protein prenylation and is lethal to cultured *P. falciparum*, suggesting that production of isoprenyl substrates for protein prenylation is an essential function of isoprenoid biosynthesis in the parasite^14^. In addition, inhibition of parasite prenyltransferase activity halts parasite replication^15^,^16^,^17^,^18^, providing compelling evidence that protein prenylation is indispensable for malaria parasite growth.

Since protein prenyltransferase activity is required by *P. falciparum*, identification of prenyltransferase substrates will likely reveal additional antimalarial targets. Bioinformatic approaches have been previously used to predict a limited number of potential prenylated proteins in the malaria genome^19^. However, since *Plasmodium* spp. are evolutionarily divergent from organisms used to generate these models, and few prenylated proteins have been experimentally confirmed in malaria parasites, it is not clear how well bioinformatics algorithms perform in predicting prenyltransferase substrates for *Plasmodium* spp. In this work, we use a chemical labeling approach to metabolically tag, potentially, the full complement of prenylated proteins in asexual *P. falciparum* parasites. Our approach was to metabolically incorporate an alkyne-modified isoprenoid analogue into the pool of prenyltransferase protein substrates. This additional alkyne functional group permits selective binding of prenylated proteins to streptavidin beads, via click chemistry with biotin-azide. The resulting prenylated proteins were identified by subsequent tryptic digestion and LC-MS analysis, coupled with bioinformatics analysis.

## Results

### An alkyne-functionalized isoprenoid analogue is metabolically incorporated into malaria parasites

**Figure 1:**
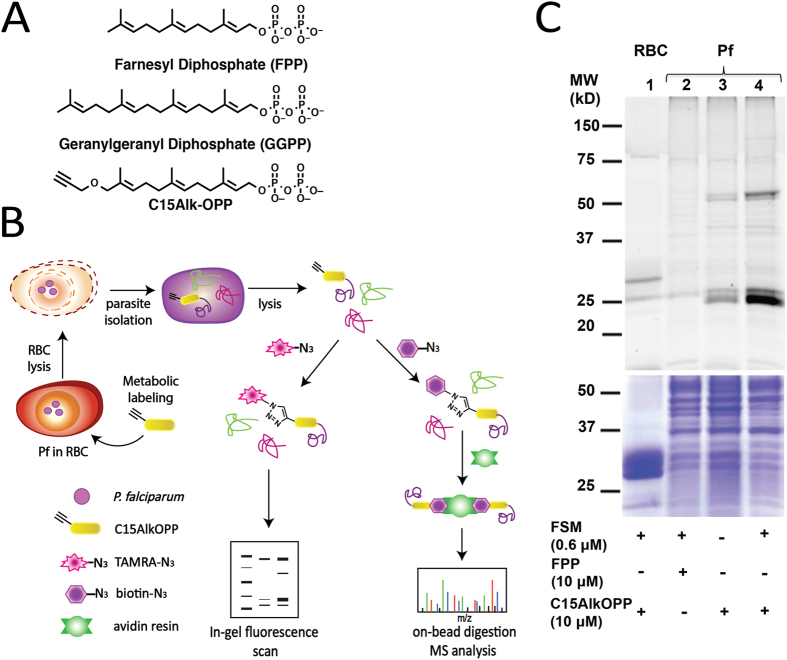
The C15AlkOPP probe allows tagging of prenylated proteins for in-gel fluorescence labeling and pulldown for proteomic analysis. (**A**) Structures of native isoprenoid substrates of prenylation, farnesyl pyrophosphate (FPP) and geranylgeranyl pyrophosphate (GGPP), and the probe analog C15AlkOPP. (**B**) Scheme for metabolic labeling using the C15AlkOPP probe followed by selective labeling or enrichment using click chemistry. In-gel fluorescence and proteomic analysis of prenylated proteins were facilitated through click reactions with fluorescent reporter TAMRA-N_3_ and affinity handle biotin-N_3_, respectively. (**C**) Labeling of prenylated proteins visualized through in-gel fluorescence (top panel) in *P. falciparum* lysates. Lane 1: C15AlkOPP + FSM in red blood cells; lane 2: FPP + FSM in Pf; lane 3: C15AlkOPP in Pf; lane 4: C15AlkOPP + FSM in Pf. Total protein loading by Coomassie blue stain is shown in purple (bottom panel).

To identify the prenylated proteins in *P. falciparum* using an alkyne-containing isoprenoid analogue, we first tested for metabolic incorporation of the compound C15AlkOPP (Fig. 1A), which was previously employed to identify prenylated proteins in mammalian cells^20^,^21^. This probe structurally resembles the native isoprenoid substrates farnesyl pyrophosphate (FPP) and geranylgeranyl pyrophosphate (GGPP) used for prenylation of proteins, and is a substrate for both mammalian FT and GGT-1^22^. Red blood cells infected with *P. falciparum* were exposed to the probe in the presence or absence of fosmidomycin (FSM), an established inhibitor of isoprenoid biosynthesis in *P. falciparum*^23^,^24^, followed by release of the intact parasites via mild detergent treatment. The free parasites were then lysed and the resulting lysates subjected to copper-catalyzed click reaction with TAMRA-N_3_, which generates a stable cycloaddition product between the alkyne-tagged prenylated proteins and TAMRA-N_3_ to allow visualization of labeled proteins (Fig. 1B). The samples were then fractionated via SDS-PAGE and subjected to in-gel fluorescence imaging (Fig. 1C, top panel).

Fluorescent protein bands were observed at approximately 25 and 50 kDa in samples obtained from parasites treated with C15AlkOPP (lane 3); a number of weaker bands, including species near 37 and 150 kDa, were also observed. A substantial enhancement of labeling ensued upon co-administration of FSM (lane 4), suggesting that depletion of the endogenous FPP pool results in increased incorporation of the analogue; similar results with C15AlkOPP have been observed in mammalian cells treated with lovastatin^20^. Replacing the probe with FPP showed only limited labeling in these regions (lane 2), indicating that the alkyne analogue is a viable tool to tag cellular prenylated proteins.

It should be noted that *P. falciparum* parasites develop within human erythrocytes. Although human prenyltransferases have not been identified in the mature erythrocyte proteome^25^, we evaluated for the possibility that human erythrocyte proteins could incorporate C15AlkOPP probe in the absence of parasite infection. Human red blood cells were thus also treated with C15AlkOPP and FSM, and did not demonstrate significant labeling (lane 1). The band observed above 25 kDa is almost certainly an artifact due to a highly abundant protein band in this region as shown in the Coomassie stained gel (Fig. 1C, bottom panel); given its size, that protein may represent a hemoglobin dimer^26^. Overall, these results indicate that the C15AlkOPP probe successfully labels prenylated proteins in the malaria parasite with minimal interference from human proteins.

### The C15AlkOPP probe is elongated by *P. falciparum* FPPS/GGPPS

**Figure 2:**
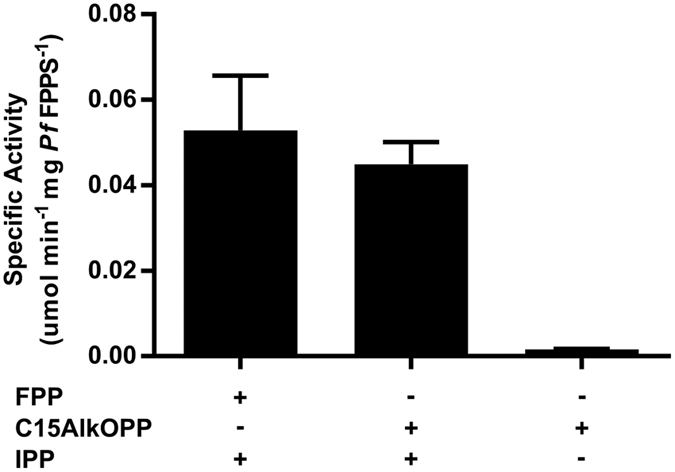
*P. falciparum* farnesyl pyrophosphate synthase (*Pf*FPPS) accepts C15AlkOPP as a substrate. Specific activity (mean ± S.E.M.) of *Pf*FPPS, measured using an absorbance-based phosphate release assay. Left to right, enzyme activity in the presence of FPP and IPP, C15AlkOPP probe and IPP, or C15AlkOPP alone. No activity was seen in absence of *Pf*FPPS (data not shown). Three replicates were performed for each condition.

As noted above, C15AlkOPP is a substrate for the mammalian FT and GGT-1 enzymes. Previously reported metabolic labeling data suggests that C15AlkOPP can also be used to label substrates of GGT-2. However, those results do not preclude elongation of C15AlkOPP to C20AlkOPP prior to prenyltransferase-catalyzed incorporation. Hence, we next sought to investigate whether C15AlkOPP can be elongated to C20AlkOPP in malaria parasites. Unlike other organisms that express a series of prenyl synthases, *P. falciparum* produces a single multi-functional enzyme (*Pf*FPPS/GGPPS, hereafter *Pf*FPPS) that is responsible for synthesis of geranyl pyrophosphate, farnesyl pyrophosphate, and geranylgeranyl pyrophosphate^27^,^28^. To evaluate whether the parasite enzyme may utilize the C15AlkOPP probe as a substrate, we assayed purified recombinant *Pf*FPPS protein. As expected, *Pf*FPPS uses IPP to elongate its natural substrate, FPP, thus generating GGPP. Similarly, we found that *Pf*FPPS also effectively uses C15AlkOPP as a substrate (Fig. 2). Thus, while it is not clear whether the elongated analogue is a substrate for the malarial GGTs, the production of that species maximizes the likelihood that our single probe strategy using C15AlkOPP will function to tag both farnesylated and geranylgeranylated proteins in the parasite.

### Bioinformatic analysis of the *P. falciparum* proteome affords a list of putative prenylated proteins

To date, only a limited number of proteins with canonical C-terminal prenylation motifs have been demonstrated to be bona fide substrates in *P. falciparum*^14^,^29^,^30^,^31^. Prior to proteomic analysis, we performed a bioinformatic investigation to create a list of all possible prenyltransferase substrates present in the *P. falciparum* proteome. FASTA sequences of proteins from the *Plasmodium falciparum* genome reference isolate 3D7 proteome (UniProt ID UP000001450) with possible C-terminal –CaaX, –CXC, and –CC prenylation motifs were analyzed using Prenylation Prediction Suite (PrePS)^32^. Out of 90 protein sequences with Cys at the 4^th^ position from the C-terminus (–CaaX), a total of 8 proteins were predicted to be prenylated (Table 1). This group contains 5 proteins whose molecular masses are close to 25, 37, 50, and 150 kDa, similar to what we observed experimentally via in-gel fluorescence labeling (Fig. 1C). Furthermore, five of these proteins contain basic residues upstream of the putatively prenylated Cys, a typical pattern observed for most prenylated proteins. In addition to these predicted proteins, we examined those that were not recognized by PrePS but met additional criteria for prenylation: basic residues upstream of Cys at the −1 to −5 positions and a hydrophobic or aromatic residue at the +2 position. Seven proteins that satisfy these additional parameters were thus identified from the proteome database (Table 2). A previous study reported 8 proteins that satisfied these criteria based on the *P. falciparum* genome, which included a DEAD/DEAH box Helicase and Methionyl-tRNA formyltransferase (along with six of the seven predicted in our analysis)^30^. However, neither of these two proteins contains a C-terminal CaaX sequence in the reference *Plasmodium falciparum* isolate 3D7 proteome database (although one of them does in another database, see Figure S1). Interestingly, the remaining protein listed in Table 2 (but not identified in the aforementioned genomic analysis), PRL protein tyrosine phosphatase, does appear to be a real prenylated protein. Recent computational docking studies^33^ suggested that it is a substrate and this has been confirmed through additional *in vitro* assays^30^.

Proteins with C-terminal –CXC and –CC motifs were also extracted from the proteome and analyzed in PrePS. As expected, Rab proteins were predicted to be substrates of geranylgeranyl transferase type II (GGT-2) with high degrees of probability (Table 3). The molecular weights of these small GTPases are consistent with the bands observed near 25 kDa via in-gel fluorescence labeling (Fig. 1C). No proteins other than those belonging to the Rab family were identified as potential substrates, suggesting that there are no known proteins with these –CXC and –CC sequences that have the specific neighboring residues upstream of Cys necessary to render them substrates for GGT-2. In aggregate, our bioinformatic analysis using PrePS suggests that the *P. falciparum* proteome contains 8 –CaaX, 3 –CXC, and 7 –CC proteins (18 total) that may be prenyltransferase substrates.

### Proteomic analysis of the prenylated proteins in *P. falciparum*

After validating the use of C15AlkOPP as a prenylation probe in *P. falciparum*, we next sought to determine the molecular identity of prenylated proteins in the parasite. To reduce cellular production of FPP, which would compete with probe incorporation, cultured asexual *P. falciparum* were treated with sub-lethal (50% of the half-maximal inhibitory concentration) concentrations FSM. Fosmidomycin-treated parasites were grown in the presence of either C15AlkOPP probe or FPP as a negative control. Following labeling, parasites were freed from host erythrocytes, and parasite protein lysates were subjected to click reaction with biotin-N3; the affinity handle conferred by biotin permits the selective enrichment and isolation of labeled prenylated proteins upon pull-down through the strong biotin-avidin interaction (Fig. 1B). Proteins were washed under stringent conditions (1% SDS and 8 M urea) followed by on-bead trypsin digestion. Equal amounts of peptides from C15AlkOPP- and FPP-treated samples, were pre-fractionated and analyzed by nano-flow liquid chromatography and tandem mass spectrometry (MS/MS), followed by database searching against *Plasmodium falciparum* (UniProt ID UP000001450) and *Homo sapiens* proteomes (UniProt ID UP000005640). After data processing, 445 proteins were identified at 99.0% minimum probability with at least 2 identified peptides from 9073 spectra, and 98.0% minimum peptide confidence within a 1% false discovery rate, for both C15AlkOPP- and FPP-treated samples.

Our labeling strategy was designed to enrich for prenyltransferase substrates labeled with C15AlkOPP and/or C20AlkOPP using a biotin pull-down approach. Proteomic studies that use such enrichment methods are sometimes complicated due to nonspecific adsorption of proteins onto the avidin-coated beads used in these experiments. To address that issue, our approach employed a quantitative comparison based on spectral counting between the samples treated with C15AlkOPP and those treated with FPP. We used the average total spectral counts for each identified protein to calculate the enrichment of proteins (fold-change) across three replicates of samples treated with C15AlkOPP probe versus FPP. The full list of spectral counts and identities are provided (Supplemental Table S1). Proteins with potential prenylation motifs (–CaaX, –CC, –CXC on their C-terminus) were extracted from that data and summarized in Table 4. A total of 15 prenylated proteins are listed, including 14 of the 18 predicted in our bioinformatics analysis. One additional protein (Q81LH7) bearing the –CaaX sequence CNFM, which was not predicted by PrePS, was also identified. Conversely, four of the –CaaX sequences predicted by PrePS were not observed (Q81583, Q81EC5, Q81E80 and Q81EK2 with –CaaX sequences CLVF, CTIM, CKQC and CNIM, respectively). All Rab proteins predicted to be prenylated by PrepPS were identified.

The list of all proteins identified in our analysis (Supplemental Table S1) is ordered based on the fold change between the C15AlkOPP- and FPP-treated samples. The top 12 proteins in that list correspond to the first 12 entries in Table 4 (9 to 33-fold change). For each of these top 12 hits, very few spectral counts were observed in the absence of probe, giving high confidence that these represent bona fide prenylated proteins. In contrast, numerous proteins were found below the 6-fold threshold. In those cases, substantial spectral counts were observed in the absence of probe; we attribute those proteins to nonspecific adsorption. However, the last 3 entries in Table 4, while manifesting low levels of spectral counts in the C15AlkOPP-treated samples, gave no spectral counts in the FPP-treated samples, suggesting that they may represent true, low abundance hits; one of those three, SNARE Ykt6.1, has been confirmed as a prenyltransferase substrate^31^. It is worth noting that the aforementioned DEAD/DEAH box Helicase and Methionyl-tRNA formyltransferase suggested by previous investigators as possible prenylated proteins based on bioinformatics analysis^30^ are not present in the list of proteins in Table S1. Finally, it should be noted that human Rab homologs were also identified and grouped into clusters with the parent proteins from *P. falciparum* (Supplementary Table S1 and Figure S2), since the data analysis was conducted using both the *H. sapiens* and *P. falciparum* databases. However, for each identified Rab, the change in spectral counts in the presence of probe was always higher for the candidate malarial proteins than their human homologs. Additionally, we found, in each case, that protein probabilities for parasite sequences were higher than those for the cognate human orthologs. Together, these characteristics indicate that the Rab proteins identified upon metabolic labeling of *P. falciparum* are indeed malarial in origin and that the human candidates are artifacts of sequence similarities.

## Discussion

Due to the ongoing spread of drug resistance, there is a pressing need for new therapies to treat malaria. Evidence strongly suggests that protein prenylation is required for asexual development of the *P. falciparum* malaria parasite; several distinct chemotypes of prenyltransferase inhibitors exhibit potent antimalarial activity^15^,^16^,^18^,^34^,^35^,^36^. Given the essential nature of protein prenylation, it follows that the functions of prenyltransferase substrates themselves are necessary for parasite replication. Thus, identification of prenylated proteins in *P. falciparum* may reveal new, essential, and highly valuable targets for antimalarial drug development. Such targeting could be accomplished either indirectly by interfering with prenylation or directly via inhibition of their cognate functions.

Here we present the first experimentally determined catalog of prenylated proteins (the “prenylome”) of blood stage *P. falciparum*. We have identified prenyltransferase substrates through the use of metabolic labeling with a novel, alkyne-containing isoprenoid analogue. We find that the *P. falciparum* farnesyl pyrophosphate synthase (FPPS) successfully elongates the probe (which is a derivative of FPP) to generate the cognate 20-carbon (GGPP derivative) probe. Therefore, we believe our *in vivo* metabolic labeling approach has likely captured the full complement of both farnesylated and geranylgeranylated proteins in *P. falciparum*, with the exception of prenylated proteins with very low levels of expression during blood-stage development that may not react in our derivatization strategy.

Eukaryotic systems possess three different protein prenyltransferases: farnesyltransferase (FT) and geranylgeranyltransferase type I (GGT-1) commonly recognize the same motif (the CaaX box) that includes the cysteine of their substrates they modify, and are thus referred to as CaaX prenyltransferases, whereas geranylgeranyltransferase type II (GGT-2, also called Rab geranylgeranyltransferase) recognizes an alternative motif^37^. Active prenyltransferases consist of two polypeptide subunits, α and β; FT and GGT-1 typically share an α subunit. As has previously been suggested^19^, we find that experimentally confirmed prenyltransferase substrates of *P. falciparum* parasites possess canonical motifs that indicate the presence of both CaaX prenyltransferases and Rab geranylgeranyltransferases. *P. falciparum* lysate has previously been shown to possess both FT and GGT-1 activity^12^. The current annotation of the *P. falciparum* genome indicates a full complement of genes encoding the candidate prenyltransferases, as follows: *Pf*FT [PF3D7_1242600 (α subunit) and PF3D7_1147500 (β subunit)], *Pf*GGT-1 [PF3D7_1242600 (α subunit; shared with *Pf*FT) and PF3D7_0602500 (β subunit)], and *Pf*GGT-2 [PF3D7_1442500 (α subunit) and PF3D7_1214300 (β subunit)].

Our work experimentally confirms that protein prenylation in *P. falciparum* reflects a more modest set of prenylated proteins than is observed in fungi or higher eukaryotes, including humans. According to PRENbase (http://mendel.imp.ac.at/PrePS/PRENbase/), a curated online database of protein prenylation across sequenced genomes, prenylated proteins in the human genome are spread into 43 clusters of paralogous proteins^38^. Biological functions of prenylated proteins are well conserved, even amongst unicellular eukaryotes, as 42 similarly defined clusters are present across fungal genomes. In stark contrast, we find robust experimental evidence for a total of only thirteen prenylated proteins in *P. falciparum*, with suggestive evidence for an additional two probable prenyltransferase substrates. While a restricted prenylome has been suggested bioinformatically for malaria parasites, our study provides important evidence that there are not unrecognized, non-canonical motifs used by the *P. falciparum* prenyltransferases.

During asexual replication, *P. falciparum* is an obligate parasite of human erythrocytes, and relies on vesicle-mediated trafficking of erythrocyte cytoplasm and hemoglobin, as well as export of essential proteins for remodeling of the erythrocyte membrane and cytoplasm. The importance of membrane trafficking to *P. falciparum* development is underscored by the restricted biological functions of the malaria prenylome. Interestingly, we find that the majority of proteins in the *P. falciparum* prenylome belong to a single cluster of paralogous proteins, the Rab family of small GTPases, classic regulators of endomembrane trafficking. These proteins likely make up the broad band at 25 kDa found in our in-gel fluorescence images upon C15OPP labeling (Fig. 1C), as well as in studies using radiolabeling of parasites with [^3^H]-geranylgeranyl pyrophosphate^39^. Unsurprisingly, of the eleven Rabs annotated in *P. falciparum*, we found all ten predicted to be geranylgeranylated by GGT-2^40^.

In addition, three more prenyltransferase substrates in *P. falciparum* (two SNARE proteins and a phosphatidylinositol 3-phosphate binding protein) are also likely to function in membrane trafficking. Notably absent from the *P. falciparum* prenylome are a number of GTPase superfamilies, including Ras and Rho, typical of other unicellular eukaryotes and metazoans. This restricted use of protein prenylation for a single biological function reflects the complement of small GTPases that has been suggested to have been present in the last common eukaryotic ancestor, prior to the dramatic expansions of paralogous GTPase gene families^41^,^42^. The limited collection of GTPases and prenylated proteins that we find in *P. falciparum* is therefore not unique to this parasite, but is shared with other Alveolates in this lineage, including several other important mammalian parasitic pathogens, such as *Cryptosporidium*, *Toxoplasma*, and *Eimeria*.

We identify only four confirmed prenylated proteins in *P. falciparum* that possess a canonical CaaX motif, which should serve for recognition and modification by either FT or GGT-1. Bioinformatic analyses are insufficient to indicate whether a given CaaX-containing protein is farnesylated or geranylgeranylated. However, experimental evidence suggests that at least one of these proteins, PF14_0359, a Hsp40 analog, is specifically farnesylated. In *P. falciparum*, metabolic labeling with [^3^H]-farnesyl pyrophosphate, but not [^3^H]-geranylgeranyl pyrophosphate, identifies a dominant band at approximately 50 kDa^39^. As the remaining proteins in the malaria prenylome are between 23–38 kDa, this finding most likely represents farnesyl modification of *Pf*Hsp40 (48 kDa)^43^.

*P. falciparum* expresses an expanded repertoire of molecular chaperones, comprising 2% of the overall genome, and including 49 Hsp40 superfamily members in total^44^. However, while other eukaryotes typically express up to five type I Hsp40s, *Pf*Hsp40 is the sole, cytosolic type I Hsp40 homolog in the malaria parasite. In other organisms, farnesylation of orthologous type I Hsp40s has been well described, and is required for the biological functions of these chaperones in mediating protein stability^45^,^46^. The cellular function of *Pf*Hsp40 in *P. falciparum* has yet to be explored, although immunofluorescence microscopy indicates that this protein is cytosolic^43^. However, data from proteomics and yeast two-hybrid studies indicate it may play a role in trafficking to the RBC membrane^47^. The extent to which farnesylation plays a role in the localization or functions of *Pf*Hsp40 remains to be explored.

Our work contributes to an increasingly complete picture of post-translational hydrophobic modifications in blood-stage *P. falciparum*. The identification of a limited set of CaaX proteins has important implications for understanding the evolution of this modification process, as well as the active work developing novel antimalarial therapies targeted to isoprenoid synthesis and prenyltransferases.

## Methods

### *P. falciparum* tissue culture

All culturing was done with *Plasmodium falciparum* genome reference strain 3D7. 3D7 was obtained from the Malaria Research and Reference Reagent Resource Center (strain MRA-102, contributed by D. J. Carucci, ATCC, Manassas, Virginia). Parasites were grown in RPMI-1640 media (Sigma-Aldrich, SKU R4130) supplemented with 27 mM sodium bicarbonate, 11 mM glucose, 5 mM HEPES, 1 mM sodium pyruvate, 0.37 mM hypoxanthine, 0.01 mM thymidine, 10 μg ml^−1^ gentamycin (Sigma-Aldrich) and 0.5% Albumax (Life Technologies) with a 2% suspension of human erythrocytes under an atmosphere of 5% CO_2_, 5% O_2_, balance N_2_ and incubated at 37 °C, as previously described^24^,^48^. For in-gel fluorescence, 40 mL of culture was used per replicate. Samples destined for mass spectroscopy analysis were derived from 200 mL of culture per replicate. For all experiments, cultures were adjusted to 4% of red blood cells infected (4% parasitemia) at experiment start. Cultures were treated with fosmidomycin (Life Technologies) to a final concentration of 600 nM (approximately half IC_50_). Pyrophosphate probes or prenyl pyrophosphates (Echelon Biosciences) were added to a final concentration of 10 μM. After compounds were added, cultures were mixed thoroughly and incubated for 24 hours.

After 24 hours, cultures were saponin lysed as previously described^24^, with modifications. In brief, cells were pelleted and washed with PBS before being lysed with 1% saponin in PBS. Saponin lyses red blood cell (RBC) membranes but not parasite cell membranes, thus freeing the parasites from the RBCs. The lysed mixture was pelleted, and the loose RBC membrane layer and supernatant removed, leaving a parasite pellet. This pellet was washed with PBS, centrifuged again, the supernatant removed, and stored at −80 °C.

RBC controls were performed with 5 mL per replicate of 2% hematocrit in supplemented RPMI media with no parasites. RBCs were pelleted without saponin since internal RBC proteins are released with lysis. This volume corresponds to the volume of saponin-freed parasites from 200 mL total culture at 4% parasitemia.

### In-gel fluorescence labeling

RBC controls and saponin-lysed pellets of *P. falciparum*, treated with or without FSM and FPP or C15AlkOPP, were suspended in 300 μL lysis buffer (10 mM PO_4_^3−^, 137 mM NaCl, 2.7 mM KCl, 2.4 μM PMSF, benzonase nuclease, protease inhibitor cocktail and 1% SDS) and sonicated 6 to 8 times for 2 seconds in 10-second intervals. Click reactions were performed on 100 μg samples of protein lysate (1 μg/μL) with 25 μM TAMRA-N_3_, 1 mM TCEP, 0.1 mM TBTA, and 1 mM CuSO_4_ at room temperature for one hour. Proteins were precipitated using a ProteoExtract precipitation kit (Calbiochem) to remove excess click chemistry reagents. Protein pellets were dissolved in 1X Laemlli loading buffer and heated at 95 °C for 5 minutes. Samples were fractionated using 12% SDS PAGE gels and imaged via in-gel fluorescence using a BioRad FX Molecular Imager with 542/568 nm excitation/emission wavelengths. Gels were stained with 1X Coomasie blue stain followed by destaining to visualize protein loading.

### Cloning and expression of *Pf*FPPS

The gene *PfFPPS* (PF3D7_1128400) was PCR amplified from *P. falciparum* 3D7 cDNA (primers *Pf*FPPS Fwd 5′-CTCACCACCACCACCACCATGCUGAGAACGAGCAGAATAACCAAGATTC-3′; *Pf*FPPS Rev 5′-ATCCTATCTTACTCACTCAAGCGCCTGTAAACAAAATGTCC-3′) and cloned into pBG1861 using ligation independent cloning as previously described^49^. The cloned *PfFPPS* sequence was verified by Sanger sequencing. The primers used add coding for a six histidine tag at the N-terminus of the gene to allow for nickel affinity purification.

Subsequently, pBG1861 was transformed into ArticExpress (DE3) RIL *E. coli* (Agilent Technologies). Cultures were grown in LB media with 100 μg/mL ampicillin at 37 °C and 200 rpm until mid-log phase, at which point they were cooled to 8 °C. Expression was induced with 0.5 mM isopropyl β-D-1-thiogalactopyranoside (IPTG), 5 μM geraniol and 5 μM farnesol overnight at 8 °C and 200 rpm. Following induction, cells were pelleted and then lysed by sonication in a solution of 25 mM Tris pH 7.5, 250 mM NaCl, 1 mM MgCl_2_, 10% v/v glycerol, 20 mM imidazole, 1 mM dithiothreitol (DTT), 1 mg/mL lysozyme, 200 μM phenylmethylsulfonyl fluoride (PMSF) 0.3 U/mL benzonase nuclease (Novagen), and EDTA-free protease inhibitor (Roche). 6-histidine tagged protein was purified from soluble lysate over Ni-NTA resin (Goldbio). The resin with bound protein was washed with 25 mM Tris pH 7.5, 250 mM NaCl, 1 mM MgCl_2_, 10% v/v glycerol, 20 mM imidazole, 1 mM dithiothreitol (DTT). Bound protein was then eluted with 25 mM Tris pH 7.5, 250 mM NaCl, 1 mM MgCl_2_, 10% v/v glycerol, 300 mM imidazole, 1 mM dithiothreitol (DTT).

Next, the elutant was further purified over a HiLoad 16/60 Superdex 200 gel filtration column (GE Healthcare) using an AKTAExplorer 100 FPLC (GE Healthcare). The FPLC buffer was 250 mM NaCl, 25 mM Tris pH 7.5, and 1 mM MgCl_2_, 10% glycerol v/v. Fractions enriched with *Pf*FPPS, as seen by a strong band at ~44 kDa on a Coomassie-stained SDS-PAGE gel, were pooled and concentrated by centrifugation using Amicon Ultra-15 centrifugal filter units (EMD Millipore). Concentrated protein was supplemented with 1 mM DTT, was flash-frozen in liquid N_2,_ and was then stored at −80 °C prior to use. Protein concentration was measured by a BCA protein assay kit (Thermo Scientific).

### Isoprenyl pyrophosphate synthase assay

Following purification, release of pyrophosphate during GGPP/C20AlkOPP synthesis from FPP/C15AlkOPP by *Pf*FPPS was monitored using the EnzChek phosphate assay kit (Life Technologies), as previously described^50^. Reactions were performed in a 50 μL volume, with final reagent concentrations as follows: 250 mM NaCl, 50 mM Tris pH 7.5, 1 mM MgCl_2_, 1 U/mL purine nucleoside phosphorylase (PNP), 0.2 mM 2-Amino-6-mercapto-7-methylpurine riboside (MESG), 0.1 U/mL yeast inorganic pyrophosphatase (New England Biolabs), and 2 μM purified *Pf*FPPS, and, where indicated, 100 μM IPP, FPP (Echelon Biosciences) and/or C15AlkOPP. All reagents save *Pf*FPPS were pre-warmed to 37 °C. Reactions were initiated by the addition of *Pf*FPPS, after which absorbance at 360 nm was recorded over a 30 min period with a BMG POLARStar plate reader preheated to 37 °C. Absorbance monitoring was performed in clear 96-well flat-bottomed plates. Enzyme reactions were linear with respect to time and enzyme concentration. Absorbance units were converted to μM phosphate using a phosphate standard curve.

### Pull-down of labeled proteins

Protein lysates (1.5 mg/mL) from parasites treated with FSM and FPP or C15AlkOPP were subjected to click reactions with 100 μM biotin-N_3_, 50 mM TCEP, 10 mM TBTA, and 50 mM CuSO_4_ for 90 minutes at room temperature. Excess reagents were removed by protein precipitation using 1 volume of chloroform, 4 volumes of CH_3_OH, and 3 volumes of PBS. Proteins were precipitated in between two immiscible phases by centrifugation at 4,500 ×  *g* for 5 minutes. The aqueous layer was discarded and 4 volumes of CH_3_OH was added, followed by centrifugation at 4,500 ×  *g* for 3 minutes to pellet the proteins. Proteins were dissolved in 1% SDS in PBS buffer (1.5 mg/mL) and incubated with 300 μL of NeutrAvidin^®^ agarose resin (Thermo Scientific) for 90 minutes. Resin samples were washed to remove unbound proteins with 3-mL volumes of 3 × 1% SDS in PBS, 1 × PBS, 3 × 8 M urea, and 3 × 50 mM NH_4_HCO_3_. Resin was suspended in 300 μL of 50 mM NH_4_HCO_3_ and combined with 5 μg trypsin (sequencing grade, Promega Corp.) for overnight digestion at 37 °C. Supernatants were collected by washing the resin with 200 μL × 4 of 50 mM NH_4_HCO_3_ and samples were lyophilized.

### Proteomic analysis

#### Sample preparation for MS/MS analysis

Lyophilized peptides were dissolved in 200 mM NH_4_COO. Aliquots from resulting peptide solutions (20 μg) were obtained to prepare 0.25 μg/μL solutions. Each sample was loaded in SDB-XC extraction disk (3 M, USA) packed in stage tips conditioned with 80% acetonitrile (ACN) and equilibrated with 200 mM NH_4_HCO_2_. Samples were washed with 200 mM NH_4_HCO_2_ and eluted into three fractions using 40 μL of 6%, 11%, and 17% ACN in H_2_O. Each sample was dissolved in 100 μL of 5% ACN and 0.1% TFA in H_2_O and loaded onto packed extraction disks in stage tips that were conditioned (80% ACN and 0.1% TFA in H_2_O) and equilibrated (5% ACN and 0.1% TFA in H_2_O). Peptides were eluted with 80% ACN with 0.1% TFA in H_2_O, lyophilized, and dissolved in 0.1% formic acid.

#### LC-MS/MS analysis of tryptic digested peptides

LC-MS/MS analyses were carried out using an RSLCnano System (Dionex, UK) and an Orbitrap Fusion Tribrid mass spectrometer (Thermo Scientific). Samples were directly loaded and eluted at a flow rate of 300 nL/min onto a reverse-phase column (75 μm i.d., 450 mm) packed with ProntoSIL C18AQ 3 μm media (Bischoff, Germany) that was prepared in-house. The peptides were eluted with buffer A (0.1% formic acid in H_2_O) and buffer B (0.1% formic acid in CH_3_CN) in the following gradient segments of buffer B: 17 mins, 0–2%; 60 mins, 2–25%; 2 mins, 25–44%; 2 mins, 44–76%; 3 mins, 76%; and 2 mins, 76–2%. The eluted peptides from the column were sprayed into a nanospray ion source on an Orbitrap Fusion Tribrid mass spectrometer set to record single microscan FTMS scan events at a resolution of 30000 over the m/z range 300–1500 Da in positive ion mode, with charge states of 2–7 included. The top 15 data-dependent CID MS/MS were triggered from the FTMS scan and introduced into the Orbitrap- Fusion ion trap. The collision energy was set to 35% and activation Q to 0.25 with the scan range and ion trap scan rate both set to normal. The automatic gain control (AGC) target values were set to optimal conditions using 500,000 for MS1 and 5,000 for MS2^51^. Dynamic exclusion was allowed once for a 90-second duration.

#### Proteomic data processing

The raw files were searched using Sequest embedded in Proteome Discoverer (version 1.4.0.288, Thermo Scientific) against the *Plasmodium falciparum* 3D7 isolate (ID UP000001450) appended with *Homo sapiens* (ID UP000005640) from UnipProtKB/SwissProt^52^. The precursor mass tolerance was set to 10 ppm and the fragment mass tolerance was set to 0.6 Da. A variable modification was set as oxidized methionine. The enzyme was set to Trypsin (Full) and up to 4 missed cleavages were allowed. A decoy search was also performed.

The resulting msf files were processed in Scaffold (version 4.4.1, Proteome Software Inc., Portland, OR) through searching with X! Tandem (version 2010.12.01.1, GPM Organization). Glu → pyro-Glu of the N-terminus, ammonia-loss of the N-terminus, Gln → pyro-Glu of the N-terminus and oxidation of methionine were specified in X! Tandem. Peptide identifications were accepted if they could be established at greater than 95% by the Scaffold Local FDR algorithm. Protein identifications were accepted if they could be established at greater than 99.0% probability and contained at least 2 identified peptides. Protein probabilities were assigned by the Protein Prophet algorithm^53^. Proteins that contained similar peptides and could not be differentiated based on MS/MS alone were grouped to satisfy the principles of parsimony. Proteins sharing significant peptide evidence were grouped into clusters. Fold changes in enrichment between probe-treated and control samples were calculated using total weighted spectra; in cases where no spectral counts were observed (zero), those were imputated to a value of 1 in the Scaffold software for subsequent analysis.

## Additional Information

**How to cite this article**: Suazo, K. F. *et al*. Global proteomic analysis of prenylated proteins in *Plasmodium falciparum* using an alkyne-modified isoprenoid analogue. *Sci. Rep.* **6**, 38615; doi: 10.1038/srep38615 (2016).

**Publisher's note:** Springer Nature remains neutral with regard to jurisdictional claims in published maps and institutional affiliations.

## Supplementary Material

Supplementary Information

## Figures and Tables

**Table 1:** Candidate prenyl transferase substrates from the *P. falciparum* 3D7 proteome, which contain canonical CaaX motifs and were identified by PrePS.

Accession No.	Name	C-term	Mol. wt. (kDa)	PrePS
Q8I346	SNARE protein (putative)	KKQCCSIM	23	FT*** GGT-1***
Q8I583	Ulp1 protease (putative)	ISQGCLVF	123	FT* GGT-1*
Q8IKN0	Uncharacterized protein	QRRMCNIM	38	FT* GGT-1**
Q8IEC5	Uncharacterized protein	KKKKCTIM	95	FT*** GGT-1***
Q8IL88	Hsp40 subfamily A (putative)	GRVACAQQ	48	FT**
C0H5D3	SNARE protein (putative)	NNQCCSLY	26	FT*
Q8IE80	Uncharacterized protein	NIAACKQC	34	FT*
Q8IEK2	Uncharacterized protein	NRLKCNIM	73	GGT-1**

PrePS was used to predict the likelihood of prenylation of the proteins identified: *low, **intermediate, ***high. Underlined residues indicate putative sites for prenylation.

**Table 2:** Candidate prenyl transferase substrates from the *P. falciparum* 3D7 proteome, which were not identified by PrePS but possess additional, promising CaaX features.

Accession No.	Name	C-term	Mol. wt. (kDa)	Basic residue at −1 to −5	Hydrophobic residue at +2	Aromatic residue at +2
Q8IIN1	Protein tyrosine phosphatase	NCLRKCHFM	25	+	+	+
Q8ILH7	Uncharacterized protein	KKRNKCNFM	51	+	+	+
Q8I3K7	Membrane skeletal protein IMC1-related	QRNLYCSYA	34	+	+	+
Q8I0W8	Deoxyribodipyrimidine photolyase	KREKKCVAS	129	+	+	−
O77306	Ser/Thr protein kinase	NKKNSCAYT	157	+	+	−
Q9NLB7	Uncharacterized protein	NYNFLCLYI	10	−	+	+
O77380	CPSF	DLENMCSFL	339	−	+	+

Underlined residues indicate putative sites for prenylation.

**Table 3:** Candidate prenyl transferase substrates from the *P. falciparum* 3D7 proteome, which possess –CXC and –CC C-terminal motifs for possible geranylgeranylation.

Accession No.	Name	C-term	Mol. wt. (kDa)	PrePS
-CXC				
Q8IL79	Copper transporter putative	ADPACCGC	27	No
Q8IM51	Secreted ookinete adhesive protein	ECSCSCSC	23	No
Q8IHR8	Rab6	NMLSKCLC	24	GGT-2**
Q7K6B0	Rab18	ESRSNCAC	23	GGT-2***
Q8I3W9	Rab1a	SPQSFCSC	24	GGT-2***
-CC				
Q8IHW0	Myosin heavy chain subunit, putative	ELNMFKCC	208	No
C6KST4	Uncharacterized protein	IKKKKMRCC	33	No
Q8II49	Conserved Plasmodium protein	TKKFFPCC	30	No
Q8IHW1	Conserved Plasmodium protein	KTKKCYCC	19	No
Q8IAL1	Uncharacterized protein	GKRFLGCC	9	No
Q7K6A8	Rab1b	KDTKKKCC	23	GGT-2***
O96193	Rab5a	TLSKKGCC	27	GGT-2***
Q8I274	Rab5c	EETKKKCC	24	GGT-2***
Q76NM4	Rab11a	TKKKNKCC	25	GGT-2***
Q8I5A9	Rab2	SRSGFSCC	24	GGT-2***
C0H516	Rab7	KMYKSRCC	24	GGT-2***
C0H5G2	Rab11b	NMNKVKCC	24	GGT-2**

PrePS was used to predict the likelihood of prenylation of the proteins identified: *low, **intermediate, ***high. Underlined residues indicate putative sites for prenylation.

**Table 4:** Prenylated proteins in *Plasmodium falciparum*, identified by C15AlkOPP labeling and proteomics, with –CaaX, -CXC, and –CC prenylation motifs.

Protein Name	Accession No^a^./Gene ID^b^	Mol. wt. (kDa)^a^	Biological Process^c^	Cellular Component^c^	Spectral Count	FPP	Fold Change	Percent coverage (%)		C-term sequence^c^	Confirmed prenylation	PrePS^d^
					C15Alk-OPP			C15Alk-OPP	FPP			
Rab6	Q8IHR8PF3D7_1144900	24	vesicular transport (putative)	trans Golgi, cytosolic^29^	33,32,33	0,0,0	33	70,70,59	0,0,0	NMLSKCLC	Yes^29^	GGT-2**
HSP40 subfamily A	Q8IL88PF3D7_1437900	48	protein folding^43^, protein translocation (putative)^54^	cytosolic, RBCmembrane^43^	39,34,37	1,2,0	28	41,35,33	2,5,0	GRVACAQQ	No	FT**
Rab7	C0H516PF3D7_0903200	24	retrograde transport^55^	endosomes^55^	25,27,25	0,0,0	26	59,66,66	0,0,0	KMYKSRCC	No	GGT-2***
Rab1b	Q7K6A8PF3D7_0512600	23	ER to Golgi transport (putative)^40^	ER, Golgi (putative)^40^	19,12,17	0,0,0	16	37,35,31	0,0,0	PDTKKKCC	No	GGT-2***
Rab1a	Q8I3W9PF3D7_0513800	24	ER to Golgi transport^40^	ER, Golgi^40^	11,10,12	0,0,0	11	17,21,22	0,0,0	SPQSFCSC	No	GGT-2***
Rab2	Q8I5A9PF3D7_1231100	24	ER to Golgi transport (putative)^40^	ER, Golgi (putative)^40^	25,21,28	2,0,0	18	58,56,57	11,0,0	SRSGFSCC	No	GGT-2***
Rab5c	Q8I274PF3D7_0106800	24	retrograde transport^14^	endosomes^14^	18,15,14	0,0,0	16	43,35,33	0,0,0	EETKKKCC	Yes^14^	GGT-2***
Rab11a	Q76NM4PF3D7_1320600	25	formation of inner membranecomplex^56^, recycling endosome transport (putative)	vesicles^56^	12,17,12	0,0,0	14	41,42,41	0,0,0	TKKKNKCC	No	GGT-2***
Rab5a	O96193PF3D7_0211200	27	retrograde transport^14^	endosomes^14^	9,9,11	0,0,0	9.7	25,29,29	0,0,0	TLSKKGCC	Yes^14^	GGT-2***
Rab18	Q7K6B0PF3D7_0807300	23	mobilization of lipid droplets (putative)^57^	Parasitopho-rous vacuole mem brane (putative)^57^	9,7,11	1,1,1	9	28,24,28	6,6,6	ESRSNCAC	No	GGT-2***
Rab11b	C0H5G2PF3D7_1340700	24	transport (putative)	membrane (putative)	8,10,9	0,0,0	9	30,25,31	0,0,0	NMNKVKCC	No	GGT-2**
SNARE Ykt6.2	C0H5D3PF3D7_1324700	26	vesicular fusion, transport (putative)^58^	membrane (putative)	5,6,7	0,0,0	6	14,18,24	0,0,0	KNNQCCSLY	No	FT*
SNARE Ykt6.1	Q8I346PF3D7_0910600	23	vesicular fusion, transport (putative)^58^	Golgi, cytosolic, vacuolar^31^	3,2,2	0,0,0	2.3	17,13,11	0,0,0	LKQCCSIM	Yes^58^	FT***GGT-1***
*Uncharac-terized*	*Q8IKN0PF3D7_1460100*	*38*	*PI3P binding, trafficking to the food vacuole*^59^	*food vacuole lumen*^59^	*0,2,1*	*0,0,0*	*1.3*	*0,8,5*	*0,0,0*	*QRRMCNIM*	*No*	FT*GGT-1**
*Uncharac-terized*	*Q8ILH7PF3D7_1428700*	*51*			*0,1,3*	*0,0,0*	*1.7*	*0,3,5*	*0,0,0*	*KRNKCNFM*	*No*	*—*

For each protein, fold-change indicates the ratio of the average total spectral counts obtained following C15AlkOPP- versus FPP-labeling, across three experimental replicates. An imputation of 1 was employed to calculate fold changes. Spectral counts and percent coverage for each replicate are shown. Two uncharacterized proteins (protein identifiers and associated data are italicized) containing predicted prenylation motifs were identified with low spectral counts. Underlined residues indicate putative sites for prenylation. ^a^Protein accession numbers including theoretical molecular weights and ^b^gene IDs were obtained from UniProt and PlasmoDB, respectively. ^C^Unless otherwise referenced from previous reports, biological processes and cellular component of identified proteins were obtained from UniProt. ^d^PrePS was used to predict the likelihood of prenylation of the proteins identified: *low, **intermediate, ***high.
