# Supplementary material for: Global proteomic analysis of prenylated proteins in Plasmodium falciparum using an alkyne-modified isoprenoid analogue
Source: Sci Rep. 2016 Dec 7;6:38615. doi: 10.1038/srep38615 (PMC5141570; doi:10.1038/srep38615)
Supplement: Supplementary Information [file srep38615-s1.pdf]

Supporting Information

Global proteomic analysis of the prenylated proteins in *Plasmodium falciparum* using an alkyne-modified isoprenoid analogue

Kiall F. Suazo,[†] Chad Schaber,[‡] Charuta C. Palsuledesai,[†] Audrey R. Odom,[‡,*] Mark Distefano[†,*]

[†]*Department of Chemistry, University of Minnesota, Minneapolis, MN 55455 USA*

[‡]*Departments of Pediatrics and of Molecular Microbiology, Washington University School of Medicine, St. Louis, MO 63110 USA*

**corresponding authors: Mark Distefano: diste001@umn.edu; Audrey Odom: odom_a@kids.wustl.edu*

Table S1. List of total proteins identified in the proteomic analysis across three replicates. Proteins with putative prenylation motifs are highlighted in blue. Proteins with similar peptides identified are grouped into clusters. Undefined values in the spectral count and percent coverage columns are assigned with 1.

No.	Identified Proteins	Accession Number	Molecular Weight	Fold Change	Spectral counts						Percent Coverage					
					C15AlkOPP			FPP			C15AlkOPP			FPP		
					1	2	3	1	2	3	1	2	3	1	2	3
1	Cluster of PfRab6, GTPase OS=Plasmodium falciparum (isolate 3D7) GN=Rab6 PE=3 SV=2 (Q8IHR8_PLAF7)	Q8IHR8_PLAF7	24 kDa	33	33	32	33	0	0	0	70.00%	69.60%	58.90%	1	0.00%	1
**1.1**	**PfRab6, GTPase OS=Plasmodium falciparum (isolate 3D7) GN=Rab6 PE=3 SV=2**	**Q8IHR8_PLAF7**	**24 kDa**	**33**	**33**	**32**	**33**	**0**	**0**	**0**	**70.00%**	**69.60%**	**58.90%**	**1**	**1**	**1**
1.2	Ras-related protein Rab-44 OS=Homo sapiens GN=RAB44 PE=1 SV=1	A0A087WXI0_HUMAN	111 kDa	1	8	0	0	0	0	0	1.08%	1	1	1	0.00%	1
1.3	Ras-related protein Rab-39A OS=Homo sapiens GN=RAB39A PE=1 SV=2	RB39A_HUMAN	25 kDa	1	0	0	0	0	0	0	1	1	1	1	1	1
1.4	Ras-related protein Rab-6A OS=Homo sapiens GN=RAB6A PE=1 SV=3	RAB6A_HUMAN	24 kDa	1	0	0	0	0	0	0	1	1	1	1	1	1
1.5	Ras-related protein Rab-6B OS=Homo sapiens GN=RAB6B PE=1 SV=1	RAB6B_HUMAN	23 kDa	1	0	0	0	0	0	0	1	1	1	1	1	1
**2**	**HSP40, subfamily A, putative OS=Plasmodium falciparum (isolate 3D7) GN=PF14_0359 PE=4 SV=1**	**Q8IL88_PLAF7**	**48 kDa**	**28**	**39**	**34**	**37**	**1**	**2**	**0**	**41.00%**	**34.70%**	**33.50%**	**1.89%**	**5.19%**	**1**
3	Cluster of PfRab7, GTPase OS=Plasmodium falciparum (isolate 3D7) GN=Rab7 PE=3 SV=1 (C0H516_PLAF7)	C0H516_PLAF7 [2]	24 kDa	26	25	27	25	0	0	0	58.70%	65.50%	65.50%	1	1	1
**3.1**	**PfRab7, GTPase OS=Plasmodium falciparum (isolate 3D7) GN=Rab7 PE=3 SV=1**	**C0H516_PLAF7**	**24 kDa**	**26**	**25**	**27**	**25**	**0**	**0**	**0**	**58.70%**	**65.50%**	**65.50%**	**1**	**1**	**1**
3.2	Ras-related protein Rab-7a OS=Homo sapiens GN=RAB7A PE=1 SV=1	C9J8S3_HUMAN	18 kDa	9.7	9	10	10	0	0	0	26.20%	26.90%	26.90%	1	1	1
4	Cluster of Rab1b, GTPase OS=Plasmodium falciparum (isolate 3D7) GN=Rab1b PE=4 SV=1 (Q7K6A8_PLAF7)	Q7K6A8_PLAF7 [2]	23 kDa	22	24	18	23	1	0	0	37.00%	35.50%	31.00%	6.79%	0.00%	1
**4.1**	**Rab1b, GTPase OS=Plasmodium falciparum (isolate 3D7) GN=Rab1b PE=4 SV=1**	**Q7K6A8_PLAF7**	**23 kDa**	**16**	**19**	**12**	**17**	**1**	**0**	**0**	**37.00%**	**35.50%**	**31.00%**	**5.50%**	**1**	**1**
**4.2**	**PfRab1a OS=Plasmodium falciparum (isolate 3D7) GN=Rab1a PE=3 SV=1**	**Q8I3W9_PLAF7**	**24 kDa**	**11**	**11**	**10**	**12**	**1**	**0**	**0**	**17.40%**	**21.30%**	**21.70%**	**5.31%**	**0.00%**	**1**
4.3	Ras-related protein Rab-15 OS=Homo sapiens GN=RAB15 PE=1 SV=1	RAB15_HUMAN	24 kDa	1	11	0	9	0	0	0	14.60%	1	10.40%	1	1	1
4.4	Ras-related protein Rab-37 OS=Homo sapiens GN=RAB37 PE=1 SV=1	A8MZI4_HUMAN (+2)	22 kDa	1	7	0	7	0	0	0	5.76%	1	5.76%	1	1	1
4.5	Ras-related protein Rab-12 OS=Homo sapiens GN=RAB12 PE=1 SV=3	RAB12_HUMAN	27 kDa	1	7	0	7	0	0	0	4.51%	1	4.51%	1	1	1
4.6	Ras-related protein Rab-35 (Fragment) OS=Homo sapiens GN=RAB35 PE=1 SV=1	F5H157_HUMAN (+1)	21 kDa	1	0	0	0	0	0	0	1	1	1	1	1	1
4.7	Ras-related protein Rab-1B OS=Homo sapiens GN=RAB1B PE=1 SV=1	A0A087WTI1_HUMAN	19 kDa	1	13	7	0	0	0	0	24.70%	18.50%	1	1	1	1
4.8	Ras-related protein Rab-1A OS=Homo sapiens GN=RAB1A PE=1 SV=3	RAB1A_HUMAN	23 kDa	1	13	7	10	0	0	0	19.50%	14.60%	14.60%	1	1	1
4.9	Ras-related protein Rab-3A OS=Homo sapiens GN=RAB3A PE=1 SV=1	RAB3A_HUMAN	25 kDa	1	7	0	0	0	0	0	5.00%	1	1	1	1	1
4.1	Ras-related protein Rab-10 OS=Homo sapiens GN=RAB10 PE=1 SV=1	RAB10_HUMAN	23 kDa	1	0	0	0	0	0	0	1	1	1	1	1	1
4.11	Ras-related protein Rab-4B (Fragment) OS=Homo sapiens GN=RAB4B PE=1 SV=1	M0R0X1_HUMAN (+1)	21 kDa	1	0	0	0	0	0	0	1	1	1	1	1	1
4.12	HCG1995540, isoform CRA_b OS=Homo sapiens GN=RAB4B PE=1 SV=1	Q6PIK3_HUMAN	14 kDa	1	0	6	0	0	0	0	1	19.80%	1	1	1	1
4.13	Ras-related protein Rab-8B (Fragment) OS=Homo sapiens GN=RAB8B PE=1 SV=1	H0YNE9_HUMAN (+1)	22 kDa	1	0	0	0	0	0	0	1	1	1	1	1	1
4.14	Ras-related protein Rab-1B OS=Homo sapiens GN=RAB1B PE=1 SV=1	RAB1B_HUMAN	22 kDa	1	0	0	0	0	0	0	1	1	1	1	1	1
4.15	Ras-related protein Rab-8A OS=Homo sapiens GN=RAB8A PE=1 SV=1	RAB8A_HUMAN	24 kDa	1	0	0	0	0	0	0	1	1	1	1	1	1
4.16	Ras-related protein Rab-15 (Fragment) OS=Homo sapiens GN=RAB15 PE=1 SV=1	J3QSF4_HUMAN	16 kDa	1	0	0	0	0	0	0	1	1	1	1	1	1
4.17	Ras-related protein Rab-30 (Fragment) OS=Homo sapiens GN=RAB30 PE=1 SV=1	E9PMJ1_HUMAN (+2)	19 kDa	1	0	0	0	0	0	0	1	1	1	1	1	1
4.18	Ras-related protein Rab-3B OS=Homo sapiens GN=RAB3B PE=1 SV=2	RAB3B_HUMAN	25 kDa	1	0	0	0	0	0	0	1	1	1	1	1	1
4.19	Ras-related protein Rab-14 OS=Homo sapiens GN=RAB14 PE=1 SV=4	RAB14_HUMAN (+1)	24 kDa	1	0	0	0	0	0	0	1	1	1	1	1	1
5	Cluster of Rab2, GTPase OS=Plasmodium falciparum (isolate 3D7) GN=Rab2 PE=3 SV=1 (Q8I5A9_PLAF7)	Q8I5A9_PLAF7	24 kDa	18	25	21	28	2	0	0	57.70%	56.30%	57.30%	10.80%	1	1
**5.1**	**Rab2, GTPase OS=Plasmodium falciparum (isolate 3D7) GN=Rab2 PE=3 SV=1**	**Q8I5A9_PLAF7**	**24 kDa**	**18**	**25**	**21**	**28**	**2**	**0**	**0**	**57.70%**	**56.30%**	**57.30%**	**10.80%**	**1**	**1**
5.2	Ras-related protein Rab-2A OS=Homo sapiens GN=RAB2A PE=1 SV=1	E9PKL7_HUMAN (+1)	21 kDa	1	0	0	0	0	0	0	1	1	1	1	1	1
5.3	Putative uncharacterized protein DKFZp313C1541 OS=Homo sapiens GN=DKFZp313C1541 PE=1 SV=1	Q5HYI5_HUMAN (+1)	23 kDa	1	0	0	0	0	0	0	1	1	1	1	1	1
5.4	Ras-related protein Rab-2A (Fragment) OS=Homo sapiens GN=RAB2A PE=1 SV=1	H0YD31_HUMAN	16 kDa	1	0	0	0	0	0	0	1	1	1	1	1	1
6	Cluster of Rab5c, GTPase OS=Plasmodium falciparum (isolate 3D7) GN=Rab5c PE=3 SV=1 (Q8I274_PLAF7)	Q8I274_PLAF7	24 kDa	16	18	15	14	0	0	0	42.50%	34.60%	32.70%	1	1	0.00%
**6.1**	**Rab5c, GTPase OS=Plasmodium falciparum (isolate 3D7) GN=Rab5c PE=3 SV=1**	**Q8I274_PLAF7**	**24 kDa**	**16**	**18**	**15**	**14**	**0**	**0**	**0**	**42.50%**	**34.60%**	**32.70%**	**1**	**1**	**1**
6.2	Ras-related protein Rab-5C OS=Homo sapiens GN=RAB5C PE=1 SV=2	RAB5C_HUMAN	23 kDa	1	6	4	4	0	0	0	5.09%	5.09%	5.09%	1	1	1
6.3	Ras-related protein Rab-5A OS=Homo sapiens GN=RAB5A PE=1 SV=2	RAB5A_HUMAN	24 kDa	1	0	0	0	0	0	0	1	1	1	1	1	0.00%
6.4	Ras-related protein Rab-5B (Fragment) OS=Homo sapiens GN=RAB5B PE=1 SV=1	F8VUA5_HUMAN (+2)	13 kDa	1	0	0	0	0	0	0	1	1	1	1	1	1
7	Cluster of Rab11a, GTPase OS=Plasmodium falciparum (isolate 3D7) GN=Rab11a PE=1 SV=1 (Q76NM4_PLAF7)	Q76NM4_PLAF7	25 kDa	14	12	17	12	0	0	0	40.70%	42.10%	41.20%	1	0.00%	1
**7.1**	**Rab11a, GTPase OS=Plasmodium falciparum (isolate 3D7) GN=Rab11a PE=1 SV=1**	**Q76NM4_PLAF7**	**25 kDa**	**14**	**12**	**17**	**12**	**0**	**0**	**0**	**40.70%**	**42.10%**	**41.20%**	**1**	**1**	**1**
7.2	Ras-related protein Rab-11A (Fragment) OS=Homo sapiens GN=RAB11A PE=3 SV=1	H3BMH2_HUMAN	18 kDa	1	0	0	0	0	0	0	1	1	1	1	0.00%	1
7.3	Ras-related protein Rab-25 OS=Homo sapiens GN=RAB25 PE=1 SV=1	A0A0C4DGX5_HUMAN (-	28 kDa	1	0	0	0	0	0	0	1	1	1	1	1	1
**8**	**Rab5a, GTPase OS=Plasmodium falciparum (isolate 3D7) GN=Rab5a PE=1 SV=1**	**O96193_PLAF7**	**27 kDa**	**9.7**	**9**	**9**	**11**	**0**	**0**	**0**	**25.10%**	**28.50%**	**29.40%**	**1**	**1**	**1**
**9**	**PfRab18, GTPase OS=Plasmodium falciparum (isolate 3D7) GN=rab18 PE=3 SV=1**	**Q7K6B0_PLAF7**	**23 kDa**	**9**	**9**	**7**	**11**	**1**	**1**	**1**	**27.90%**	**24.40%**	**27.90%**	**5.47%**	**5.47%**	**5.47%**
**10**	**Rab11b, GTPase (Fragment) OS=Plasmodium falciparum (isolate 3D7) GN=Rab11b PE=3 SV=1**	**C0H5G2_PLAF7**	**24 kDa**	**9**	**8**	**10**	**9**	**0**	**0**	**0**	**30.30%**	**25.50%**	**30.80%**	**1**	**1**	**1**
11	Karyopherin beta OS=Plasmodium falciparum (isolate 3D7) GN=PFE1195w PE=4 SV=1	Q8I3M5_PLAF7	127 kDa	8.2	11	12	10	2	1	0	11.10%	12.50%	9.35%	2.05%	1.16%	1
**12**	**SNARE protein, putative OS=Plasmodium falciparum (isolate 3D7) GN=PfYkt6.2 PE=4 SV=1**	**C0H5D3_PLAF7**	**26 kDa**	**6**	**5**	**6**	**7**	**0**	**0**	**0**	**14.50%**	**17.60%**	**24.00%**	**1**	**1**	**1**
13	Hexokinase OS=Plasmodium falciparum (isolate 3D7) GN=PFF1155w PE=3 SV=1	C6KT76_PLAF7	55 kDa	5.8	10	9	10	3	1	1	12.80%	15.80%	13.00%	5.88%	2.43%	2.43%
14	60S ribosomal protein L4, putative OS=Plasmodium falciparum (isolate 3D7) GN=PFE0350c PE=1 SV=1	Q8I431_PLAF7	46 kDa	5.4	10	9	8	3	1	1	19.20%	15.60%	16.10%	3.65%	1.95%	1.95%
15	Adenosylhomocysteinase OS=Plasmodium falciparum (isolate 3D7) GN=PFE1050w PE=1 SV=2	SAHH_PLAF7	54 kDa	5.4	11	8	8	2	1	2	9.39%	9.39%	9.39%	4.80%	2.71%	5.64%
16	Glyceraldehyde-3-phosphate dehydrogenase OS=Plasmodium falciparum (isolate 3D7) GN=GAPDH PE=3 SV=1	Q8IKK7_PLAF7	37 kDa	5.3	23	17	18	6	1	4	38.30%	35.60%	30.60%	19.00%	2.67%	10.40%
17	Eukaryotic translation initiation factor 3 subunit A OS=Plasmodium falciparum (isolate 3D7) GN=PFL0625c PE=3 SV=1	Q8I5S6_PLAF7	166 kDa	5	2	8	5	0	0	1	1.82%	5.01%	4.21%	1	0.00%	0.65%
18	Eukaryotic translation initiation factor 3 subunit L OS=Plasmodium falciparum (isolate 3D7) GN=PFF0590c PE=3 SV=1	C6KSW5_PLAF7	78 kDa	5	9	7	9	2	0	2	8.38%	10.70%	10.50%	4.27%	1	2.74%
19	Cluster of Serum albumin OS=Homo sapiens GN=ALB PE=1 SV=1 (A0A0C4DGB6_HUMAN)	A0A0C4DGB6_HUMAN	69 kDa	4.8	5	7	7	2	0	0	3.64%	2.48%	2.48%	2.48%	1	1
19.1	Serum albumin OS=Homo sapiens GN=ALB PE=1 SV=1	A0A0C4DGB6_HUMAN	69 kDa	1	5	7	7	2	0	0	3.64%	2.48%	2.48%	2.48%	1	1
19.2	Serum albumin OS=Homo sapiens GN=ALB PE=1 SV=2	ALBU_HUMAN	69 kDa	1	5	7	7	2	0	0	3.61%	2.46%	2.46%	2.46%	1	1
19.3	Serum albumin (Fragment) OS=Homo sapiens GN=ALB PE=1 SV=1	H7C013_HUMAN	23 kDa	1	1	0	0	0	0	0	3.55%	0.00%	0.00%	0.00%	1	1
20	Helicase 45 OS=Plasmodium falciparum (isolate 3D7) GN=H45 PE=3 SV=1	Q8IKF0_PLAF7	45 kDa	4.4	14	9	8	4	1	2	24.40%	19.80%	17.60%	11.10%	2.51%	4.52%
21	Isoleucine-tRNA ligase, putative OS=Plasmodium falciparum (isolate 3D7) GN=PF13_0179 PE=3 SV=1	Q8IDZ9_PLAF7	151 kDa	4.3	4	6	3	1	0	0	4.40%	4.64%	3.54%	0.71%	1	1
22	Falcilysin OS=Plasmodium falciparum (isolate 3D7) GN=flN PE=1 SV=1	Q76NL8_PLAF7	139 kDa	4.3	2	4	7	0	0	0	2.77%	3.60%	6.62%	0.00%	1	1
23	60S ribosomal protein L7-3, putative OS=Plasmodium falciparum (isolate 3D7) GN=PF14_0231 PE=1 SV=2	Q8ILL2_PLAF7	33 kDa	4.3	5	2	6	1	0	1	10.60%	6.71%	9.54%	3.89%	0.00%	3.53%
24	60S ribosomal protein L13 OS=Plasmodium falciparum (isolate 3D7) GN=PF08_0075 PE=1 SV=1	Q8IAX6_PLAF7	25 kDa	4.2	6	5	6	2	1	1	23.70%	20.50%	20.00%	6.98%	5.12%	4.19%
25	Uncharacterized protein OS=Plasmodium falciparum (isolate 3D7) GN=MAL13P1.237 PE=4 SV=1	Q8IDM3_PLAF7	42 kDa	4.2	8	6	7	2	0	2	24.20%	19.60%	24.70%	5.91%	1	5.91%
26	Methionine--tRNA ligase, putative OS=Plasmodium falciparum (isolate 3D7) GN=PF10_0340 PE=4 SV=1	Q8IJ60_PLAF7	104 kDa	4.2	15	13	18	7	0	3	13.60%	13.20%	18.00%	5.96%	1	3.82%
27	Multidrug resistance protein OS=Plasmodium falciparum (isolate 3D7) GN=PfMDR1 PE=3 SV=1	Q7K6A5_PLAF7	162 kDa	4	10	12	10	3	3	2	9.73%	8.67%	9.44%	2.89%	2.61%	1.27%
28	Myo-inositol 1-phosphate synthase, putative OS=Plasmodium falciparum (isolate 3D7) GN=PFE0585c PE=4 SV=1	Q8I3Y8_PLAF7	69 kDa	4	16	9	11	4	3	2	21.00%	13.10%	15.40%	7.95%	4.47%	3.31%
29	Thioredoxin peroxidase 1 OS=Plasmodium falciparum (isolate 3D7) GN=TPx1 PE=4 SV=1	Q8IL80_PLAF7	22 kDa	4	8	5	3	2	0	0	26.70%	16.40%	8.21%	16.40%	1	1
30	40S ribosomal protein SA OS=Plasmodium falciparum (isolate 3D7) GN=PF10_0264 PE=1 SV=1	RSSA_PLAF7	30 kDa	4	3	5	4	0	0	0	6.84%	12.20%	10.60%	0.00%	0.00%	1
31	Glutamine synthetase, putative OS=Plasmodium falciparum (isolate 3D7) GN=PFI1110w PE=3 SV=1	C0H551_PLAF7	63 kDa	4	7	3	2	1	0	0	9.21%	4.97%	4.79%	2.03%	1	1
32	Long-chain-fatty-acid--CoA ligase, putative OS=Plasmodium falciparum (isolate 3D7) GN=PFL1880w PE=4 SV=1	Q8I535_PLAF7	92 kDa	3.8	6	4	5	2	0	0	8.08%	5.18%	6.69%	2.90%	1	1
33	Knob-associated histidine-rich protein OS=Plasmodium falciparum (isolate 3D7) GN=PFB0100c PE=4 SV=1	Q9TY99_PLAF7	71 kDa	3.7	5	13	15	4	4	1	6.42%	11.60%	8.26%	7.80%	4.74%	2.91%
34	Hornerin OS=Homo sapiens GN=HRNR PE=1 SV=2	HORN_HUMAN	282 kDa	3.7	2	4	5	0	0	0	1.40%	1.47%	3.12%	0.00%	1	0.00%
35	Uncharacterized protein OS=Plasmodium falciparum (isolate 3D7) GN=MAL8P1.95 PE=4 SV=1	C0H4U4_PLAF7	36 kDa	3.7	3	6	2	1	1	0	8.57%	11.70%	4.13%	4.13%	4.13%	1
36	Proteasome 26S regulatory subunit, putative OS=Plasmodium falciparum (isolate 3D7) GN=PFB0260w PE=4 SV=3	O96153_PLAF7	108 kDa	3.7	3	5	3	1	0	0	2.59%	6.00%	2.80%	1.55%	0.00%	1
37	Asparagine-tRNA ligase, putative OS=Plasmodium falciparum (isolate 3D7) GN=PFB0525w PE=3 SV=1	O96198_PLAF7	71 kDa	3.7	6	4	1	1	1	0	6.39%	4.75%	1.48%	1.64%	1.64%	1
38	40S ribosomal protein S4 OS=Plasmodium falciparum (isolate 3D7) GN=PF11_0065 PE=1 SV=2	Q8IIU8_PLAF7	30 kDa	3.7	3	5	3	0	0	0	12.30%	7.66%	8.43%	0.00%	1	1
39	Conserved Plasmodium protein OS=Plasmodium falciparum (isolate 3D7) GN=PF11_0364 PE=4 SV=1	Q8II11_PLAF7	28 kDa	3.7	4	4	3	0	0	0	20.80%	16.30%	7.35%	1	1	1
40	Rab5b, GTPase OS=Plasmodium falciparum (isolate 3D7) GN=Rab5b PE=3 SV=1	Q76NM7_PLAF7	23 kDa	3.7	5	0	5	0	0	0	21.70%	1	20.80%	1	1	1
41	Phosphoglycerate kinase OS=Plasmodium falciparum (isolate 3D7) GN=PGK PE=1 SV=1	PGK_PLAF7	45 kDa	3.6	21	20	20	11	4	2	37.50%	35.60%	34.10%	17.80%	7.45%	2.64%
42	Elongation factor 2 OS=Plasmodium falciparum (isolate 3D7) GN=PF14_0486 PE=4 SV=1	Q8IKW5_PLAF7	94 kDa	3.5	13	13	16	7	0	4	10.90%	10.90%	15.00%	5.17%	1	5.29%
43	6-phosphofructokinase, putative OS=Plasmodium falciparum (isolate 3D7) GN=PFI0755c PE=3 SV=1	Q8I2Z8_PLAF7	159 kDa	3.5	3	9	2	2	1	0	2.12%	7.12%	1.34%	2.54%	0.56%	0.00%
44	S-adenosylmethionine synthase OS=Plasmodium falciparum (isolate 3D7) GN=PfSAMS PE=3 SV=1	Q7K6A4_PLAF7	45 kDa	3.4	12	11	11	5	4	1	28.90%	24.90%	18.70%	13.20%	9.95%	3.73%
45	Elongation factor 1-alpha OS=Plasmodium falciparum (isolate 3D7) GN=PF13_0304 PE=3 SV=1	Q8I0P6_PLAF7	49 kDa	3.3	20	15	25	12	4	2	25.10%	23.50%	31.80%	22.80%	12.00%	6.55%
46	Polyadenylate-binding protein OS=Plasmodium falciparum (isolate 3D7) GN=PFL1170w PE=3 SV=1	Q8I5H4_PLAF7	97 kDa	3.3	4	3	3	1	1	0	5.37%	4.69%	4.80%	1.26%	1.26%	1
47	p-type ATPase, putative OS=Plasmodium falciparum (isolate 3D7) GN=PFL0590c PE=3 SV=1	Q8I5T3_PLAF7	134 kDa	3.3	5	3	2	1	0	1	2.73%	3.97%	2.15%	1.41%	0.00%	0.91%
48	60S ribosomal protein L6-2, putative OS=Plasmodium falciparum (isolate 3D7) GN=PF13_0213 PE=1 SV=1	Q8IDV1_PLAF7	26 kDa	3.3	7	7	6	4	1	0	18.60%	15.40%	19.00%	15.40%	3.62%	1
49	Ran-binding protein, putative OS=Plasmodium falciparum (isolate 3D7) GN=PFI0490c PE=4 SV=1	C0H530_PLAF7	139 kDa	3.3	3	4	3	1	0	0	2.92%	3.84%	2.59%	1.09%	1	0.00%
50	Receptor for activated C kinase homolog, PfRACK OS=Plasmodium falciparum (isolate 3D7) GN=PfRACK PE=4 SV=1	Q8IBA0_PLAF7	36 kDa	3.3	4	4	2	1	0	1	15.50%	15.50%	12.40%	3.10%	1	6.50%
51	Ethanolamine kinase, putative OS=Plasmodium falciparum (isolate 3D7) GN=PF11_0257 PE=4 SV=1	Q8IIB7_PLAF7	50 kDa	3.2	4	4	5	2	1	0	8.27%	8.04%	5.91%	5.91%	2.60%	1
52	Peptidyl-prolyl cis-trans isomerase OS=Plasmodium falciparum (isolate 3D7) GN=PF11_0164 PE=3 SV=1	Q8IIK8_PLAF7	22 kDa	3.2	3	5	5	2	1	1	13.80%	26.20%	15.40%	9.23%	5.13%	6.67%
53	60S acidic ribosomal protein P0 OS=Plasmodium falciparum (isolate 3D7) GN=PF11_0313 PE=3 SV=1	Q8II61_PLAF7	35 kDa	3.2	3	5	5	2	0	0	11.10%	14.20%	11.10%	6.96%	0.00%	1
54	Uncharacterized protein OS=Plasmodium falciparum (isolate 3D7) GN=MAL13P1.308 PE=4 SV=1	C0H5J9_PLAF7	289 kDa	3	1	7	1	0	0	0	0.58%	3.13%	0.58%	1	0.00%	1
55	Bifunctional dihydrofolate reductase-thymidylate synthase OS=Plasmodium falciparum (isolate 3D7) GN=DHFR-TS PE=3 S	Q8I1R6_PLAF7	72 kDa	3	3	3	3	0	0	0	5.76%	4.77%	5.10%	1	1	1
56	Acyl-CoA synthetase, PfACS10 OS=Plasmodium falciparum (isolate 3D7) GN=PfACS10 PE=4 SV=1	Q8I3L4_PLAF7	77 kDa	3	2	4	3	0	1	0	4.31%	5.79%	4.31%	0.00%	1.63%	1
57	Eukaryotic translation initiation factor 3 subunit I OS=Plasmodium falciparum (isolate 3D7) GN=MAL7P1.81 PE=3 SV=1	Q8IBT2_PLAF7	37 kDa	3	2	4	3	0	0	1	7.03%	17.10%	10.70%	0.00%	1	5.50%
58	Cg4 protein OS=Plasmodium falciparum (isolate 3D7) GN=PF07_0033 PE=3 SV=1	Q8IC01_PLAF7	100 kDa	3	1	4	4	1	0	0	1.95%	4.35%	2.86%	1.95%	1	1
59	PfSec23 protein OS=Plasmodium falciparum (isolate 3D7) GN=Pfsec23 PE=2 SV=1	Q8IB60_PLAF7	86 kDa	3	3	4	2	1	1	0	2.90%	3.16%	1.45%	1.45%	1.45%	0.00%
60	T-complex protein beta subunit, putative OS=Plasmodium falciparum (isolate 3D7) GN=PFC0285c PE=3 SV=3	O97247_PLAF7	59 kDa	3	2	4	3	0	0	0	3.95%	8.83%	6.39%	1	1	1
61	Uncharacterized protein OS=Plasmodium falciparum (isolate 3D7) GN=PFE1600w PE=4 SV=1	Q8I3F1_PLAF7	60 kDa	3	2	4	3	0	0	0	6.29%	8.25%	9.04%	1	1	1
62	Transketolase OS=Plasmodium falciparum (isolate 3D7) GN=PfTK PE=4 SV=1	C6KSV3_PLAF7	76 kDa	3	3	3	3	0	0	0	4.76%	4.76%	4.17%	1	1	1
63	Macrophage migration inhibitory factor homologue OS=Plasmodium falciparum (isolate 3D7) GN=MIF PE=1 SV=1	Q8I5C5_PLAF7	13 kDa	3	2	3	4	1	0	1	18.10%	25.90%	25.90%	7.76%	1	10.30%

64	Cluster of Threonine--tRNA ligase, putative OS=Plasmodium falciparum (isolate 3D7) GN=PF11_0270 PE=3 SV=1 (Q8IIA4_	Q8IIA4_PLAF7	120 kDa	3	2	4	3	0	0	0	1.97%	4.15%	2.96%	1	1	1
64.1	Threonine--tRNA ligase, putative OS=Plasmodium falciparum (isolate 3D7) GN=PF11_0270 PE=3 SV=1	Q8IIA4_PLAF7	120 kDa	3	2	4	3	0	0	0	1.97%	4.15%	2.96%	1	1	1
64.2	Threonine--tRNA ligase, cytoplasmic OS=Homo sapiens GN=TARS PE=1 SV=3	SYTC_HUMAN	83 kDa	1	0	0	0	0	0	0	1	1	1	1	1	1
65	4-methyl-5(B-hydroxyethyl)-thiazol monophosphate biosynthesis enzyme OS=Plasmodium falciparum (isolate 3D7) GN=I	C6KTB1_PLAF7	20 kDa	3	2	3	4	1	0	0	4.23%	16.40%	10.10%	4.23%	1	1
66	NAD synthase, putative OS=Plasmodium falciparum (isolate 3D7) GN=PFI1310w PE=4 SV=1	Q8I2P2_PLAF7	98 kDa	3	3	3	3	0	0	0	4.77%	4.77%	3.34%	1	1	1
67	T-complex protein 1 subunit delta OS=Plasmodium falciparum (isolate 3D7) GN=MAL13P1.283 PE=3 SV=1	C0H5I7_PLAF7	58 kDa	3	3	3	3	0	0	0	7.37%	7.37%	7.37%	1	1	1
68	Rhoptry neck protein 3, putative OS=Plasmodium falciparum (isolate 3D7) GN=PFL2505c PE=4 SV=1	Q8I4R5_PLAF7	263 kDa	2.9	40	29	30	23	9	2	15.00%	13.80%	13.00%	10.50%	4.56%	1.40%
69	Cluster of Valine-tRNA ligase, putative OS=Plasmodium falciparum (isolate 3D7) GN=PF14_0589 PE=3 SV=1 (Q8IKL5_PLA	Q8IKL5_PLAF7	128 kDa	2.8	3	4	7	2	1	2	3.49%	5.50%	4.40%	2.11%	2.11%	3.03%
69.1	Valine-tRNA ligase, putative OS=Plasmodium falciparum (isolate 3D7) GN=PF14_0589 PE=3 SV=1	Q8IKL5_PLAF7	128 kDa	2.8	3	4	7	2	1	2	3.49%	5.50%	4.40%	2.11%	2.11%	3.03%
69.2	Valine--tRNA ligase OS=Homo sapiens GN=VARS PE=1 SV=4	SYVC_HUMAN	140 kDa	1	1	0	0	0	0	0	1.19%	1	1	0.00%	0.00%	1
70	T-complex protein 1 subunit gamma OS=Plasmodium falciparum (isolate 3D7) GN=PFL1425w PE=3 SV=1	Q8I5C4_PLAF7	61 kDa	2.8	4	3	4	0	0	2	7.56%	6.27%	7.75%	0.00%	1	4.43%
71	Pyridoxal 5'-phosphate synthase subunit Pdx1 OS=Plasmodium falciparum (isolate 3D7) GN=pdx1 PE=1 SV=1	PDX1_PLAF7	33 kDa	2.8	2	5	4	2	1	1	10.30%	12.30%	9.63%	7.97%	2.99%	2.99%
72	Cluster of Enolase OS=Plasmodium falciparum (isolate 3D7) GN=ENO PE=3 SV=1 (ENO_PLAF7)	ENO_PLAF7	49 kDa	2.7	21	19	23	12	8	3	24.20%	24.20%	24.20%	20.00%	10.80%	5.61%
72.1	Enolase OS=Plasmodium falciparum (isolate 3D7) GN=ENO PE=3 SV=1	ENO_PLAF7	49 kDa	2.7	21	19	23	12	8	3	24.20%	24.20%	24.20%	20.00%	10.80%	5.61%
72.2	Alpha-enolase OS=Homo sapiens GN=ENO1 PE=1 SV=2	ENOA_HUMAN	47 kDa	1	0	2	2	0	0	0	1	4.15%	4.15%	1	1	1
72.3	Beta-enolase OS=Homo sapiens GN=ENO3 PE=1 SV=5	ENOB_HUMAN	47 kDa	1	0	0	0	0	0	0	1	1	1	1	1	1
72.4	Enolase OS=Homo sapiens GN=ENO2 PE=1 SV=1	F5H0C8_HUMAN	35 kDa	1	0	0	0	0	0	0	1	1	1	1	1	1
73	Cluster of Heat shock protein 86 OS=Plasmodium falciparum (isolate 3D7) GN=PF07_0029 PE=1 SV=1 (Q8IC05_PLAF7)	Q8IC05_PLAF7	86 kDa	2.7	20	18	21	14	4	4	20.80%	15.70%	19.70%	16.50%	5.77%	6.71%
73.1	Heat shock protein 86 OS=Plasmodium falciparum (isolate 3D7) GN=PF07_0029 PE=1 SV=1	Q8IC05_PLAF7	86 kDa	2.7	20	18	21	14	4	4	20.80%	15.70%	19.70%	16.50%	5.77%	6.71%
73.2	Heat shock protein HSP 90-beta OS=Homo sapiens GN=HSP90AB1 PE=1 SV=4	HS90B_HUMAN	83 kDa	1	6	6	8	6	0	0	5.39%	5.39%	5.39%	5.39%	1	1
73.3	Heat shock protein HSP 90-alpha OS=Homo sapiens GN=HSP90AA1 PE=1 SV=5	HS90A_HUMAN	85 kDa	1	5	5	7	5	0	0	3.69%	3.69%	3.69%	3.69%	1	1
74	Carbamoyl phosphate synthetase OS=Plasmodium falciparum (isolate 3D7) GN=cpsSII PE=4 SV=1	Q8IEN3_PLAF7	273 kDa	2.7	2	4	2	1	1	1	1.14%	1.68%	0.84%	0.46%	0.42%	0.51%
75	Peptidase, putative OS=Plasmodium falciparum (isolate 3D7) GN=PF14_0517 PE=3 SV=1	Q8IKT5_PLAF7	89 kDa	2.7	9	8	7	5	3	0	5.63%	7.98%	8.90%	5.37%	4.71%	0.00%
76	Adenosine deaminase, putative OS=Plasmodium falciparum (isolate 3D7) GN=PF10_0289 PE=4 SV=1	Q8IJA9_PLAF7	42 kDa	2.7	10	6	8	7	1	1	19.90%	12.80%	15.30%	11.20%	3.27%	4.90%
77	Exportin 1, putative OS=Plasmodium falciparum (isolate 3D7) GN=PFC0135c PE=4 SV=2	O77312_PLAF7	148 kDa	2.7	3	3	2	0	0	0	2.87%	1.83%	1.91%	0.00%	1	1
78	Uncharacterized protein OS=Plasmodium falciparum (isolate 3D7) GN=PFC0435w PE=4 SV=1	O77361_PLAF7	154 kDa	2.7	3	4	1	0	0	0	1.62%	3.01%	1.16%	0.00%	0.00%	1
79	Histidine--tRNA ligase, putative OS=Plasmodium falciparum (isolate 3D7) GN=PF14_0428 PE=3 SV=1	Q8IL22_PLAF7	134 kDa	2.7	2	5	0	0	0	0	1.50%	3.27%	1	0.00%	1	1
80	DNAJ-like molecular chaperone protein, putative OS=Plasmodium falciparum (isolate 3D7) GN=PFI0935w PE=4 SV=1	Q8I2W2_PLAF7	43 kDa	2.7	4	3	1	1	1	0	11.10%	8.11%	4.86%	3.24%	3.24%	1
81	Uncharacterized protein OS=Plasmodium falciparum (isolate 3D7) GN=PF11_0191 PE=4 SV=1	Q8III3_PLAF7	69 kDa	2.7	2	3	3	0	0	0	4.88%	6.57%	6.73%	1	1	1
82	DEAD box helicase, UAP56 OS=Plasmodium falciparum (isolate 3D7) GN=UAP56 PE=4 SV=1	Q9TY94_PLAF7	52 kDa	2.7	2	3	3	0	0	1	4.60%	5.91%	5.47%	1	1	2.41%
83	40S ribosomal protein S19, putative OS=Plasmodium falciparum (isolate 3D7) GN=PFD1055w PE=1 SV=1	Q8IFP2_PLAF7	20 kDa	2.7	3	4	1	1	0	0	11.20%	13.50%	5.29%	5.29%	1	1
84	M1-family aminopeptidase OS=Plasmodium falciparum (isolate 3D7) GN=MAL13P1.56 PE=1 SV=1	Q8IEK1_PLAF7	126 kDa	2.6	9	11	9	5	2	4	8.29%	7.93%	6.54%	4.24%	1.75%	4.61%
85	Heat shock protein 70 (HSP70) homologue OS=Plasmodium falciparum (isolate 3D7) GN=PFI0875w PE=3 SV=1	Q8I2X4_PLAF7	72 kDa	2.6	29	29	26	21	4	7	33.10%	29.90%	26.80%	27.10%	6.90%	11.80%
86	Cluster of 14-3-3 protein, putative OS=Plasmodium falciparum (isolate 3D7) GN=MAL8P1.69 PE=3 SV=1 (C0H4V6_PLAF7)	C0H4V6_PLAF7	30 kDa	2.6	12	11	11	9	3	1	23.30%	27.50%	23.30%	27.10%	13.00%	6.11%
86.1	14-3-3 protein, putative OS=Plasmodium falciparum (isolate 3D7) GN=MAL8P1.69 PE=3 SV=1	C0H4V6_PLAF7	30 kDa	2.6	12	11	11	9	3	1	23.30%	27.50%	23.30%	27.10%	13.00%	6.11%
86.2	14-3-3 protein sigma OS=Homo sapiens GN=SFN PE=1 SV=1	1433S_HUMAN	28 kDa	1	0	0	0	1	0	0	1	1	1	4.03%	0.00%	1
86.3	14-3-3 protein beta/alpha OS=Homo sapiens GN=YWHAB PE=1 SV=3	1433B_HUMAN	28 kDa	1	0	0	0	0	0	0	1	1	1	1	0.00%	1
86.4	14-3-3 protein theta OS=Homo sapiens GN=YWHAQ PE=1 SV=1	1433T_HUMAN	28 kDa	1	0	0	0	1	0	0	1	1	1	4.08%	1	1
86.5	14-3-3 protein zeta/delta OS=Homo sapiens GN=YWHAZ PE=1 SV=1	1433Z_HUMAN (+1)	28 kDa	1	0	0	0	0	0	0	1	1	1	1	1	1
86.6	14-3-3 protein epsilon OS=Homo sapiens GN=YWHAE PE=1 SV=1	1433E_HUMAN	29 kDa	1	0	0	0	0	0	0	1	1	1	1	1	1
86.7	14-3-3 protein gamma OS=Homo sapiens GN=YWHAG PE=1 SV=2	1433G_HUMAN	28 kDa	1	0	0	0	0	0	0	1	1	1	1	1	1
86.8	14-3-3 protein eta OS=Homo sapiens GN=YWHAH PE=1 SV=4	1433F_HUMAN	28 kDa	1	0	0	0	0	0	0	1	1	1	1	1	1
87	Cell division cycle protein 48 homologue, putative OS=Plasmodium falciparum (isolate 3D7) GN=PFF0940c PE=3 SV=1	C6KT34_PLAF7	92 kDa	2.6	1	7	5	3	1	0	1.45%	9.30%	7.00%	4.59%	1.33%	1
88	Cytoadherence linked asexual protein 9(CLAG9) OS=Plasmodium falciparum (isolate 3D7) GN=CLAG9 PE=4 SV=1	Q8I2G2_PLAF7	160 kDa	2.5	18	21	11	13	6	1	9.85%	9.40%	5.07%	5.45%	3.81%	0.60%
89	Glutamate--tRNA ligase, putative OS=Plasmodium falciparum (isolate 3D7) GN=PF13_0257 PE=3 SV=1	Q8IDK7_PLAF7	101 kDa	2.5	9	8	8	5	3	2	10.80%	9.04%	9.04%	6.84%	4.17%	1.39%
90	Elongation factor 1-gamma, putative OS=Plasmodium falciparum (isolate 3D7) GN=PF13_0214 PE=4 SV=2	Q8IDV0_PLAF7	48 kDa	2.5	4	2	4	2	0	1	5.11%	5.11%	8.03%	5.11%	1	2.43%
91	60S ribosomal protein L12, putative OS=Plasmodium falciparum (isolate 3D7) GN=PFE0850c PE=3 SV=2	Q8I3T8_PLAF7	18 kDa	2.5	4	2	4	2	0	1	27.90%	17.00%	22.40%	5.45%	1	7.88%
92	Merozoite surface protein 1 OS=Plasmodium falciparum (isolate 3D7) GN=MSP1 PE=4 SV=1	Q8I0U8_PLAF7	196 kDa	2.4	21	11	19	18	0	2	12.30%	6.98%	12.60%	10.00%	0.00%	1.28%
93	Cluster of Selenium-binding protein 1 OS=Homo sapiens GN=SELENBP1 PE=1 SV=2 (SBP1_HUMAN)	SBP1_HUMAN	52 kDa	2.4	3	5	4	3	1	0	6.57%	9.75%	9.75%	6.57%	5.88%	1
93.1	Selenium-binding protein 1 OS=Homo sapiens GN=SELENBP1 PE=1 SV=2	SBP1_HUMAN	52 kDa	2.4	3	5	4	3	1	0	6.57%	9.75%	9.75%	6.57%	3.81%	1
93.2	Selenium-binding protein 1 (Fragment) OS=Homo sapiens GN=SELENBP1 PE=1 SV=7	H0Y532_HUMAN	34 kDa	1	2	0	2	0	0	0	5.88%	1	5.88%	1	1	1
94	Calcium-transporting ATPase, putative OS=Plasmodium falciparum (isolate 3D7) GN=PFA_0310c PE=3 SV=1	Q76NN8_PLAF7	139 kDa	2.3	1	3	3	1	0	1	0.81%	2.61%	3.42%	0.81%	0.00%	1.06%
95	ADP/ATP transporter on adenylate translocase OS=Plasmodium falciparum (isolate 3D7) GN=PF10_0366 PE=3 SV=1	Q8IJ34_PLAF7	34 kDa	2.3	4	1	2	1	0	1	7.97%	4.32%	4.32%	4.32%	0.00%	4.32%
96	Eukaryotic translation initiation factor 3 subunit C OS=Plasmodium falciparum (isolate 3D7) GN=PFL0310c PE=3 SV=1	Q8I5Y3_PLAF7	116 kDa	2.3	4	2	1	1	0	0	4.57%	2.13%	1.12%	1.12%	1	1
97	Sortilin, putative OS=Plasmodium falciparum (isolate 3D7) GN=PF14_0493 PE=4 SV=1	Q8IKV8_PLAF7	102 kDa	2.3	2	2	3	0	1	0	2.57%	3.13%	4.47%	1	1.79%	1
98	Uncharacterized protein OS=Plasmodium falciparum (isolate 3D7) GN=PFL2120w PE=4 SV=1	Q8I4Y9_PLAF7	155 kDa	2.3	3	2	2	0	0	0	2.47%	1.23%	1.47%	0.00%	0.00%	1
99	Suprabasin OS=Homo sapiens GN=SBSN PE=1 SV=2	SBSN_HUMAN	61 kDa	2.3	0	3	3	0	0	0	0.00%	8.47%	9.15%	0.00%	1	1
100	6-phosphogluconate dehydrogenase, decarboxylating OS=Plasmodium falciparum (isolate 3D7) GN=PF14_0520 PE=3 SV	Q8IKT2_PLAF7	53 kDa	2.3	3	2	2	1	0	0	5.56%	5.56%	4.70%	2.99%	1	1
101	Casein kinase 1, PfCK1 OS=Plasmodium falciparum (isolate 3D7) GN=PfCK1 PE=3 SV=1	C6S3F7_PLAF7 (+1)	38 kDa	2.3	3	1	3	0	0	0	6.79%	4.01%	9.26%	1	1	1
102	Eukaryotic translation initiation factor 3 subunit M OS=Plasmodium falciparum (isolate 3D7) GN=PFD0880w PE=3 SV=1	Q8I1Q5_PLAF7	51 kDa	2.3	4	2	0	0	0	0	10.50%	4.66%	0.00%	1	1	1
**103**	**SNARE protein, putative OS=Plasmodium falciparum (isolate 3D7) GN=PfYkt6.1 PE=4 SV=1**	**Q8I346_PLAF7**	**23 kDa**	**2.3**	**3**	**2**	**2**	**0**	**0**	**0**	**16.60%**	**12.60%**	**11.10%**	**1**	**1**	**1**
104	Cluster of Eukaryotic translation initiation factor 2 gamma subunit, putative OS=Plasmodium falciparum (isolate 3D7) GN	Q8ILY9_PLAF7	51 kDa	2.3	4	1	2	1	0	0	8.82%	2.80%	4.09%	2.80%	1	1
104.1	Eukaryotic translation initiation factor 2 gamma subunit, putative OS=Plasmodium falciparum (isolate 3D7) GN=PF14_	Q8ILY9_PLAF7	51 kDa	2.3	4	1	2	1	0	0	8.82%	2.80%	4.09%	2.80%	1	1
104.2	Putative eukaryotic translation initiation factor 2 subunit 3-like protein OS=Homo sapiens GN=EIF2S3L PE=5 SV=2	IF2GL_HUMAN (+1)	51 kDa	1	0	0	0	0	0	0	1	1	1	1	1	1
105	Uncharacterized protein OS=Plasmodium falciparum (isolate 3D7) GN=PFD1110w PE=4 SV=1	Q8IFN1_PLAF7	43 kDa	2.3	3	2	2	0	0	0	11.00%	2.96%	7.26%	1	1	1
106	Obg-like ATPase 1 OS=Plasmodium falciparum (isolate 3D7) GN=MAL7P1.122 PE=3 SV=1	Q8IBM9_PLAF7	45 kDa	2.3	3	2	2	0	0	1	9.41%	5.85%	5.85%	1	1	2.80%
107	60S ribosomal protein L26, putative OS=Plasmodium falciparum (isolate 3D7) GN=PFC0535w PE=1 SV=1	O77364_PLAF7	15 kDa	2.3	3	3	1	1	0	0	8.73%	8.73%	7.94%	8.73%	1	1
108	Proteasome subunit alpha type OS=Plasmodium falciparum (isolate 3D7) GN=MAL13P1.270 PE=3 SV=1	Q8IDG2_PLAF7	27 kDa	2.3	3	2	2	1	0	0	12.40%	9.13%	9.13%	4.56%	1	1
109	Importin subunit alpha OS=Plasmodium falciparum (isolate 3D7) GN=PF08_0087 PE=3 SV=1	Q8IAW0_PLAF7	61 kDa	2.3	2	2	3	0	0	0	2.20%	4.59%	4.59%	1	1	1
110	L-lactate dehydrogenase OS=Plasmodium falciparum (isolate 3D7) GN=PfLDH PE=1 SV=1	Q76NM3_PLAF7	34 kDa	2.3	9	6	8	7	2	1	23.10%	16.10%	19.90%	16.50%	3.48%	3.48%
111	Fructose-bisphosphate aldolase OS=Plasmodium falciparum (isolate 3D7) GN=PF14_0425 PE=1 SV=1	ALF_PLAF7	40 kDa	2.3	12	15	12	8	7	2	29.30%	30.90%	26.60%	21.10%	19.20%	7.59%
112	60S ribosomal protein L27, putative OS=Plasmodium falciparum (isolate 3D7) GN=PF14_0579 PE=1 SV=2	Q8IKM5_PLAF7	17 kDa	2.3	5	6	5	4	2	1	15.80%	15.80%	15.80%	15.10%	15.10%	8.22%
113	40S ribosomal protein S15A, putative OS=Plasmodium falciparum (isolate 3D7) GN=PFC0735w PE=1 SV=1	O77395_PLAF7	15 kDa	2.2	3	3	3	1	1	2	13.10%	16.90%	16.90%	6.15%	6.92%	13.10%
114	Phosphoribosylpyrophosphate synthetase OS=Plasmodium falciparum (isolate 3D7) GN=PF13_0143 PE=4 SV=1	Q8IE67_PLAF7	49 kDa	2.2	1	5	3	1	2	0	2.97%	9.38%	6.86%	2.29%	2.29%	1
115	Cluster of 60S ribosomal protein L27a, putative OS=Plasmodium falciparum (isolate 3D7) GN=PFF0885w PE=1 SV=1 (C6K	C6KT23_PLAF7	17 kDa	2.2	3	8	9	6	2	1	23.60%	29.10%	29.10%	29.10%	10.80%	10.80%
115.1	60S ribosomal protein L27a, putative OS=Plasmodium falciparum (isolate 3D7) GN=PFF0885w PE=1 SV=1	C6KT23_PLAF7	17 kDa	2.2	3	8	9	6	2	1	23.60%	29.10%	29.10%	29.10%	10.80%	10.80%
115.2	60S ribosomal protein L27a OS=Homo sapiens GN=RPL27A PE=1 SV=2	RL27A_HUMAN	17 kDa	1	0	0	0	0	0	0	1	1	1	1	1	1
116	Transcription factor with AP2 domain(S), putative OS=Plasmodium falciparum (isolate 3D7) GN=ApiAP2 PE=1 SV=1	Q8IKH2_PLAF7	92 kDa	2.2	4	3	4	3	1	1	2.83%	2.83%	2.83%	2.83%	0.98%	0.98%
117	Cluster of 40S ribosomal protein S8 OS=Plasmodium falciparum (isolate 3D7) GN=PF14_0083 PE=1 SV=1 (Q8IM10_PLAF7	Q8IM10_PLAF7	25 kDa	2.2	3	4	4	3	1	0	11.90%	17.40%	12.40%	12.40%	7.98%	1
117.1	40S ribosomal protein S8 OS=Plasmodium falciparum (isolate 3D7) GN=PF14_0083 PE=1 SV=1	Q8IM10_PLAF7	25 kDa	2.2	3	4	4	3	1	0	11.90%	17.40%	12.40%	12.40%	6.88%	1
117.2	40S ribosomal protein S8 OS=Homo sapiens GN=RPS8 PE=1 SV=2	RS8_HUMAN	24 kDa	1	0	0	0	0	0	0	1	1	1	1	1	1
117.3	40S ribosomal protein S8 OS=Homo sapiens GN=RPS8 PE=1 SV=1	Q5JR95_HUMAN	22 kDa	1	0	0	0	0	0	0	1	1	1	1	1	1
118	Catalase OS=Homo sapiens GN=CAT PE=1 SV=3	CATA_HUMAN	60 kDa	2.2	3	2	6	2	2	0	6.64%	5.31%	10.60%	3.61%	3.98%	1
119	Early transcribed membrane protein 5, ETRAMP5 OS=Plasmodium falciparum (isolate 3D7) GN=ETRAMP5 PE=4 SV=1	Q8I3F3_PLAF7	19 kDa	2.2	1	6	4	1	3	1	7.18%	27.60%	20.40%	7.18%	20.40%	7.18%
120	Erythrocyte band 7 integral membrane protein OS=Homo sapiens GN=STOM PE=1 SV=3	STOM_HUMAN	32 kDa	2.2	11	13	13	11	4	2	29.50%	35.10%	35.80%	28.10%	14.90%	10.80%
121	Cluster of 60S ribosomal protein L40/UBI, putative OS=Plasmodium falciparum (isolate 3D7) GN=PF13_0346 PE=1 SV=1 (	Q8ID50_PLAF7	15 kDa	2.2	3	7	3	4	0	0	19.50%	28.90%	28.90%	28.90%	1	1
121.1	60S ribosomal protein L40/UBI, putative OS=Plasmodium falciparum (isolate 3D7) GN=PF13_0346 PE=1 SV=1	Q8ID50_PLAF7	15 kDa	1.8	2	4	3	3	0	0	19.50%	28.90%	28.90%	28.90%	1	1
121.2	Ubiquitin-40S ribosomal protein S27a OS=Homo sapiens GN=RPS27A PE=1 SV=2	RS27A_HUMAN	18 kDa	1	2	4	1	2	0	0	16.00%	16.00%	5.77%	16.00%	1	1
121.3	Ubiquitin-60S ribosomal protein L40 (Fragment) OS=Homo sapiens GN=UBA52 PE=1 SV=1	M0R1M6_HUMAN	13 kDa	1	0	1	1	1	0	0	1	7.89%	7.89%	7.89%	1	1
121.4	Ubiquitin-60S ribosomal protein L40 OS=Homo sapiens GN=UBA52 PE=1 SV=2	RL40_HUMAN	15 kDa	1	0	4	0	2	0	0	1	19.50%	1	19.50%	1	1
122	60S ribosomal protein L8, putative OS=Plasmodium falciparum (isolate 3D7) GN=PFE0845c PE=1 SV=1	Q8I3T9_PLAF7	28 kDa	2.2	2	5	6	3	2	0	9.23%	15.00%	15.00%	10.80%	7.31%	1
123	Ribosomal protein L15 OS=Plasmodium falciparum (isolate 3D7) GN=PFD0770c PE=1 SV=1	C0H4A6_PLAF7	24 kDa	2.1	5	4	6	5	0	0	23.40%	19.50%	18.50%	23.40%	1	1
124	Cluster of Tubulin beta chain OS=Plasmodium falciparum (isolate 3D7) GN=PF10_0084 PE=3 SV=1 (TBB_PLAF7)	TBB_PLAF7	50 kDa	2.1	6	6	3	4	2	1	11.00%	11.00%	4.94%	7.64%	2.70%	2.25%
124.1	Tubulin beta chain OS=Plasmodium falciparum (isolate 3D7) GN=PF10_0084 PE=3 SV=1	TBB_PLAF7	50 kDa	2.1	6	6	3	4	2	1	11.00%	11.00%	4.94%	7.64%	2.70%	2.25%
124.2	Tubulin beta-4B chain OS=Homo sapiens GN=TUBB4B PE=1 SV=1	TBB4B_HUMAN	50 kDa	1	1	2	0	0	0	0	2.70%	2.70%	1	1	1	0.00%
125	Phosphoethanolamine N-methyltransferase OS=Plasmodium falciparum (isolate 3D7) GN=PfPMT PE=4 SV=1	Q8IDQ9_PLAF7	31 kDa	2.1	13	11	12	9	3	5	31.20%	30.80%	31.20%	21.80%	9.77%	9.77%
126	QF122 antigen OS=Plasmodium falciparum (isolate 3D7) GN=PF10_0115 PE=4 SV=1	Q8IJS7_PLAF7	132 kDa	2.1	9	6	4	3	3	3	6.50%	4.13%	4.13%	3.07%	2.90%	2.99%
127	RhopH3 OS=Plasmodium falciparum (isolate 3D7) GN=PFI0265c PE=4 SV=1	Q8I395_PLAF7	105 kDa	2.1	9	4	6	6	2	1	10.10%	6.13%	6.24%	9.25%	2.34%	1.67%
128	Pyruvate kinase OS=Plasmodium falciparum (isolate 3D7) GN=PFF1300w PE=1 SV=1	C6KTA4_PLAF7	56 kDa	2	12	15	17	12	6	4	13.30%	25.80%	23.50%	15.70%	9.00%	6.85%
129	40S ribosomal protein S3a OS=Plasmodium falciparum (isolate 3D7) GN=MAL3P7.35 PE=1 SV=1	RS3A_PLAF7	30 kDa	2	7	5	2	3	3	1	13.40%	12.20%	4.96%	4.20%	8.78%	4.58%
130	Thioredoxin-related protein, putative OS=Plasmodium falciparum (isolate 3D7) GN=PF13_0272 PE=4 SV=1	Q8IDH5_PLAF7	24 kDa	2	4	4	2	3	1	1	10.60%	16.80%	10.60%	8.65%	3.85%	7.21%
131	40S ribosomal protein S13, putative OS=Plasmodium falciparum (isolate 3D7) GN=PF13_0316 PE=1 SV=1	Q8IDB0_PLAF7	17 kDa	2	5	2	3	3	1	1	23.20%	8.61%	16.60%	14.60%	8.61%	8.61%
132	Histone H2B OS=Plasmodium falciparum (isolate 3D7) GN=PF11_0062 PE=3 SV=1	Q8IIV1_PLAF7	13 kDa	2	4	2	4	3	1	0	24.80%	16.20%	24.80%	17.10%	7.69%	1
133	Cluster of Ribosomal protein L3, putative OS=Plasmodium falciparum (isolate 3D7) GN=PF10_0272 PE=1 SV=1 (Q8IJC6_P	Q8IJC6_PLAF7	44 kDa	2	3	2	0	1	1	0	4.40%	5.70%	0.00%	2.07%	2.33%	1
133.1	Ribosomal protein L3, putative OS=Plasmodium falciparum (isolate 3D7) GN=PF10_0272 PE=1 SV=1	Q8IJC6_PLAF7	44 kDa	2	3	2	0	1	1	0	4.40%	5.70%	1	2.07%	2.33%	1
133.2	60S ribosomal protein L3 OS=Homo sapiens GN=RPL3 PE=1 SV=2	RL3_HUMAN	46 kDa	1	0	0	0	0	0	0	1	1	0.00%	1	1	1
134	Uncharacterized protein OS=Plasmodium falciparum (isolate 3D7) GN=PF14_0301 PE=4 SV=1	Q8ILE3_PLAF7	33 kDa	2	3	2	3	2	1	0	7.96%	7.96%	11.40%	7.61%	3.46%	1
135	Lysine--tRNA ligase OS=Plasmodium falciparum (isolate 3D7) GN=PF13_0262 PE=1 SV=1	Q8IDJ8_PLAF7	68 kDa	2	2	3	1	0	0	0	5.49%	5.49%	3.09%	0.00%	1	0.00%
136	Chaperonin, cpn60 OS=Plasmodium falciparum (isolate 3D7) GN=PFL1545c PE=3 SV=2	Q8I0V3_PLAF7	81 kDa	2	2	2	2	0	0	0	3.76%	3.90%	3.48%	1	0.00%	0.00%
137	Cluster of Ribosomal protein S18, putative OS=Plasmodium falciparum (isolate 3D7) GN=PF11_0272 PE=1 SV=1 (Q8IIA2_	Q8IIA2_PLAF7	18 kDa	2	2	5	3	3	0	1	10.90%	29.50%	19.90%	10.90%	1	8.97%
137.1	Ribosomal protein S18, putative OS=Plasmodium falciparum (isolate 3D7) GN=PF11_0272 PE=1 SV=1	Q8IIA2_PLAF7	18 kDa	2	2	5	3	3	0	1	10.90%	29.50%	19.90%	10.90%	1	8.97%
137.2	40S ribosomal protein S18 OS=Homo sapiens GN=RPS18 PE=1 SV=3	RS18_HUMAN	18 kDa	1	0	0	0	0	0	0	1	1	1	1	1	1
138	Glycerol-3-phosphate dehydrogenase OS=Plasmodium falciparum (isolate 3D7) GN=PFC0275w PE=3 SV=1	O97245_PLAF7	75 kDa	2	3	2	1	1	0	0	5.51%	3.68%	1.84%	1.84%	1	1

139	Glucose-6-phosphate isomerase OS=Plasmodium falciparum (isolate 3D7) GN=PF14_0341 PE=1 SV=1	Q8ILA4_PLAF7	67 kDa	2	1	4	1	0	0	0	2.07%	5.53%	2.07%	0.00%	1	1
140	40S ribosomal protein S2, putative OS=Plasmodium falciparum (isolate 3D7) GN=PF14_0448 PE=1 SV=1	Q8IL02_PLAF7	30 kDa	2	3	0	2	1	0	0	10.30%	0.00%	5.88%	2.57%	1	0.00%
141	Annexin A7 OS=Homo sapiens GN=ANXA7 PE=1 SV=3	ANXA7_HUMAN	53 kDa	2	2	4	2	1	2	0	5.12%	8.61%	6.76%	3.48%	3.48%	1
142	Proteosome subunit, putative OS=Plasmodium falciparum (isolate 3D7) GN=PF14_0025 PE=4 SV=1	Q8IM66_PLAF7	78 kDa	2	2	2	2	0	1	0	3.75%	3.75%	3.75%	0.00%	1.80%	1
143	GTP cyclohydrolase I OS=Plasmodium falciparum (isolate 3D7) GN=PFL1155w PE=4 SV=1	Q8I5H7_PLAF7	46 kDa	2	2	3	1	1	0	0	6.17%	6.17%	2.83%	2.83%	0.00%	1
144	Desmoglein-1 OS=Homo sapiens GN=DSG1 PE=1 SV=2	DSG1_HUMAN	114 kDa	2	0	3	4	2	1	0	0.00%	2.57%	6.29%	2.57%	1.81%	1
145	Tudor staphylococcal nuclease OS=Plasmodium falciparum (isolate 3D7) GN=TSN PE=4 SV=1	Q8II01_PLAF7	129 kDa	2	0	3	2	1	1	0	1	3.28%	1.91%	1.46%	1.46%	1
146	Eukaryotic translation initiation factor 2, beta, putative OS=Plasmodium falciparum (isolate 3D7) GN=PF10_0103 PE=4 S\	Q8IJT9_PLAF7	25 kDa	2	2	2	2	1	0	0	5.86%	10.80%	10.80%	5.86%	1	1
147	Protein disulfide isomerase related protein OS=Plasmodium falciparum (isolate 3D7) GN=PF11_0352 PE=4 SV=1	Q8II23_PLAF7	49 kDa	2	1	2	3	1	0	1	2.60%	5.44%	7.80%	2.84%	1	2.60%
148	Ubiquitin-activating enzyme e1, putative OS=Plasmodium falciparum (isolate 3D7) GN=PFL1245w PE=4 SV=1	Q8I5F9_PLAF7	132 kDa	2	0	2	3	0	0	0	0.00%	2.28%	3.33%	1	1	1
149	Cysteinyl-tRNA synthetase, putative OS=Plasmodium falciparum (isolate 3D7) GN=PF10_0149 PE=3 SV=2	Q8IJP3_PLAF7	80 kDa	2	2	2	2	0	0	0	2.81%	3.25%	2.81%	0.00%	1	1
150	Multidrug resistance protein 2 (Heavy metal transport family) OS=Plasmodium falciparum (isolate 3D7) GN=PfMDR2 PE=	Q8IKZ6_PLAF7	119 kDa	2	3	2	0	1	0	0	2.05%	2.73%	1	1.46%	1	1
151	T-complex protein 1, putative OS=Plasmodium falciparum (isolate 3D7) GN=PFB0635w PE=3 SV=3	O96220_PLAF7	61 kDa	2	3	2	0	1	0	0	5.90%	4.43%	1	2.21%	1	1
152	Eukaryotic translation initiation factor 3 subunit 5, putative OS=Plasmodium falciparum (isolate 3D7) GN=PFI0895c PE=4	Q8I2X0_PLAF7	37 kDa	2	2	2	2	0	1	0	6.88%	6.88%	6.88%	1	3.12%	1
153	T-complex protein 1 epsilon subunit, putative OS=Plasmodium falciparum (isolate 3D7) GN=PFC0900w PE=3 SV=1	O97282_PLAF7	59 kDa	2	3	1	2	1	0	0	4.11%	2.24%	3.74%	2.24%	1	1
154	Band 3 anion transport protein OS=Homo sapiens GN=SLC4A1 PE=1 SV=3	B3AT_HUMAN	102 kDa	1.9	46	35	34	39	15	5	22.70%	21.60%	18.80%	24.10%	17.90%	6.15%
155	Cluster of Actin-1 OS=Plasmodium falciparum (isolate 3D7) GN=PFL2215w PE=3 SV=1 (ACT1_PLAF7)	ACT1_PLAF7 [4]	42 kDa	1.9	26	19	29	19	10	9	46.50%	21.20%	37.40%	22.90%	20.80%	12.50%
155.1	Actin-1 OS=Plasmodium falciparum (isolate 3D7) GN=PFL2215w PE=3 SV=1	ACT1_PLAF7	42 kDa	3.2	9	5	12	5	2	1	21.80%	14.90%	24.70%	11.20%	7.18%	4.26%
155.2	Actin-2 OS=Plasmodium falciparum (isolate 3D7) GN=PF14_0124 PE=3 SV=1	ACT2_PLAF7	43 kDa	2	3	1	2	0	0	0	9.04%	4.79%	9.04%	1	1	1
155.3	Actin, cytoplasmic 2 OS=Homo sapiens GN=ACTG1 PE=1 SV=1	ACTG_HUMAN	42 kDa	1.6	15	9	14	13	6	5	29.90%	20.80%	30.70%	22.90%	20.80%	12.50%
155.4	Putative beta-actin-like protein 3 OS=Homo sapiens GN=POTEKP PE=5 SV=1	ACTBM_HUMAN	42 kDa	1.2	4	3	4	3	3	3	7.73%	7.73%	7.73%	7.73%	11.70%	7.73%
155.5	POTE ankyrin domain family member E OS=Homo sapiens GN=POTEE PE=1 SV=3	POTEE_HUMAN	121 kDa	1	6	4	5	4	4	4	4.47%	3.63%	3.63%	3.63%	5.02%	3.72%
155.6	POTE ankyrin domain family member F OS=Homo sapiens GN=POTEF PE=4 SV=1	A0A0D9SFL1_HUMAN	44 kDa	1	0	0	0	0	1	1	0.00%	0.00%	1	0.00%	3.81%	2.79%
155.7	POTE ankyrin domain family member C OS=Homo sapiens GN=POTEC PE=1 SV=1	A0A087WXQ7_HUMAN	43 kDa	1	0	0	0	0	0	0	1	1	1	1	1	1
155.8	POTE ankyrin domain family member B3 OS=Homo sapiens GN=POTEB3 PE=4 SV=1	A0A0G2JMU2_HUMAN (	66 kDa	1	0	0	0	0	0	1	1	0.00%	1	0.00%	1	1.89%
155.9	Beta-actin-like protein 2 OS=Homo sapiens GN=ACTBL2 PE=1 SV=2	ACTBL_HUMAN	42 kDa	1	7	2	5	4	1	1	9.31%	7.18%	7.18%	6.38%	4.26%	4.26%
155.1	POTE ankyrin domain family member I OS=Homo sapiens GN=POTEI PE=3 SV=1	POTEI_HUMAN	121 kDa	1	4	3	2	3	2	2	2.98%	2.14%	2.14%	2.14%	2.14%	1.21%
155.11	POTE ankyrin domain family member B2 OS=Homo sapiens GN=POTEB2 PE=3 SV=1	POTB2_HUMAN	62 kDa	1	0	0	0	0	0	1	1	1	1	0.00%	1	2.02%
155.12	Actin, alpha cardiac muscle 1 OS=Homo sapiens GN=ACTC1 PE=1 SV=1	ACTC_HUMAN	42 kDa	1	13	7	9	8	3	3	21.50%	14.10%	14.10%	16.20%	9.81%	8.49%
155.13	Actin, gamma-enteric smooth muscle OS=Homo sapiens GN=ACTG2 PE=1 SV=1	ACTH_HUMAN	42 kDa	1	13	7	9	8	3	3	21.50%	14.10%	14.10%	16.20%	9.84%	8.51%
155.14	Actin, cytoplasmic 1 (Fragment) OS=Homo sapiens GN=ACTB PE=1 SV=1	C9JUM1_HUMAN	11 kDa	1	7	2	4	0	0	0	46.50%	21.20%	37.40%	1	1	1
155.15	Actin, cytoplasmic 2 (Fragment) OS=Homo sapiens GN=ACTG1 PE=1 SV=7	I3L4N8_HUMAN	27 kDa	1	7	5	7	8	3	2	25.20%	15.70%	22.30%	19.00%	15.70%	7.44%
155.16	Actin, cytoplasmic 2 OS=Homo sapiens GN=ACTG1 PE=1 SV=1	J3KT65_HUMAN	22 kDa	1	8	0	0	0	0	2	32.30%	1	1	1	1	9.09%
155.17	Actin, alpha skeletal muscle OS=Homo sapiens GN=ACTA1 PE=1 SV=3	A6NL76_HUMAN	28 kDa	1	9	0	0	0	2	2	22.40%	1	1	1	8.27%	6.30%
155.18	POTE ankyrin domain family member I OS=Homo sapiens GN=POTEI PE=4 SV=1	A0A0D9SFE8_HUMAN	42 kDa	1	0	0	0	0	0	0	1	1	1	0.00%	1	0.00%
155.19	POTE ankyrin domain family member D OS=Homo sapiens GN=POTED PE=2 SV=2	POTED_HUMAN	66 kDa	1	0	0	0	0	0	1	1	1	1	1	1	1.88%
156	Spectrin alpha chain, erythrocytic 1 OS=Homo sapiens GN=SPTA1 PE=1 SV=1	A0A087WZE4_HUMAN (-	281 kDa	1.9	95	92	99	93	38	16	26.00%	26.00%	26.20%	22.30%	12.50%	6.67%
157	Cluster of Cytoadherence linked asexual protein OS=Plasmodium falciparum (isolate 3D7) GN=MAL7P1.229 PE=4 SV=1 (C	C0H4J7_PLAF7 [3]	166 kDa	1.9	15	10	10	9	3	6	8.68%	7.48%	7.34%	6.99%	1.84%	5.01%
157.1	Cytoadherence linked asexual protein 3.1 OS=Plasmodium falciparum (isolate 3D7) GN=RhopH1(3.1) PE=4 SV=2	O77310_PLAF7	167 kDa	1.8	14	9	10	9	3	6	8.68%	7.48%	7.34%	6.99%	1.83%	5.01%
157.2	Cytoadherence linked asexual protein OS=Plasmodium falciparum (isolate 3D7) GN=MAL7P1.229 PE=4 SV=1	C0H4J7_PLAF7	166 kDa	1.7	2	2	0	1	0	0	1.51%	1.51%	0.00%	0.72%	1	1
157.3	Cytoadherence linked asexual protein 3.2 OS=Plasmodium falciparum (isolate 3D7) GN=RhopH1(3.2) PE=4 SV=3	O77309_PLAF7	167 kDa	1.4	8	6	9	8	3	5	5.65%	4.45%	6.14%	5.79%	1.84%	3.81%
157.4	Cytoadherence linked asexual protein 2 OS=Plasmodium falciparum (isolate 3D7) GN=PFB0935w PE=4 SV=2	O96279_PLAF7	171 kDa	1	0	0	0	1	0	0	0.00%	0.00%	1	0.69%	1	1
158	Ornithine aminotransferase OS=Plasmodium falciparum (isolate 3D7) GN=OAT PE=1 SV=1	OAT_PLAF7	46 kDa	1.9	21	15	18	13	10	5	17.40%	13.80%	17.60%	13.80%	8.94%	6.28%
159	Rhoptry-associated protein 1, RAP1 OS=Plasmodium falciparum (isolate 3D7) GN=PF14_0102 PE=4 SV=1	Q8ILZ1_PLAF7	90 kDa	1.9	9	8	8	5	7	0	11.60%	11.50%	11.80%	5.50%	8.44%	1
160	Erythrocyte membrane protein band 4.2 OS=Homo sapiens GN=EPB42 PE=1 SV=3	EPB42_HUMAN	77 kDa	1.9	13	11	12	12	6	1	12.70%	13.50%	15.10%	10.70%	8.10%	1.88%
161	60S ribosomal protein L21e, putative OS=Plasmodium falciparum (isolate 3D7) GN=PF14_0240 PE=1 SV=1	Q8ILK3_PLAF7	19 kDa	1.9	5	7	5	6	1	2	18.00%	24.80%	11.20%	24.20%	4.97%	12.40%
162	Nucleic acid binding protein, putative OS=Plasmodium falciparum (isolate 3D7) GN=MAL13P1.233 PE=4 SV=1	Q8IDN4_PLAF7	25 kDa	1.9	3	4	6	2	3	2	20.90%	17.50%	19.00%	5.69%	9.00%	11.40%
163	60S ribosomal protein L18a OS=Plasmodium falciparum (isolate 3D7) GN=PF13_0224 PE=1 SV=1	Q8IDS6_PLAF7	22 kDa	1.9	3	6	4	5	1	0	16.80%	16.80%	16.80%	22.30%	4.35%	1
164	40S ribosomal protein S11, putative OS=Plasmodium falciparum (isolate 3D7) GN=PFC0775w PE=1 SV=2	O77381_PLAF7	19 kDa	1.9	5	3	5	3	3	0	18.00%	11.80%	12.40%	18.00%	18.00%	1
165	Desmoplakin OS=Homo sapiens GN=DSP PE=1 SV=3	DESP_HUMAN	332 kDa	1.8	0	4	6	3	2	0	0.00%	1.36%	2.44%	0.70%	0.70%	1
166	Protein disulfide-isomerase OS=Plasmodium falciparum (isolate 3D7) GN=PfPDI-8 PE=3 SV=1	C0H4Y6_PLAF7	56 kDa	1.8	9	7	6	7	4	0	17.80%	14.30%	15.70%	17.00%	6.42%	0.00%
167	Uncharacterized protein OS=Plasmodium falciparum (isolate 3D7) GN=PF08_0091 PE=4 SV=1	Q8IAV1_PLAF7	144 kDa	1.8	7	2	2	4	1	1	7.02%	1.98%	1.98%	3.97%	0.66%	0.91%
168	Serine hydroxymethyltransferase OS=Plasmodium falciparum (isolate 3D7) GN=PFL1720w PE=1 SV=1	Q8I566_PLAF7	50 kDa	1.8	6	4	1	2	0	3	8.82%	8.82%	2.49%	4.98%	1	4.30%
169	Aquaporin-1 OS=Homo sapiens GN=AQP1 PE=1 SV=3	AQP1_HUMAN (+1)	29 kDa	1.8	4	4	3	3	2	0	13.00%	13.00%	13.00%	13.00%	13.00%	1
170	Elongation factor 1-beta OS=Plasmodium falciparum (isolate 3D7) GN=PfEF-1beta PE=4 SV=1	Q8I320_PLAF7	32 kDa	1.8	4	4	3	1	4	0	13.00%	16.30%	13.00%	8.33%	13.00%	1
171	GTP-binding nuclear protein ran/tc4 OS=Plasmodium falciparum (isolate 3D7) GN=PF11_0183 PE=4 SV=1	Q7KQK6_PLAF7	25 kDa	1.8	7	8	5	5	4	2	21.50%	22.90%	26.60%	22.90%	15.90%	10.70%
172	Endoplasmin homolog, putative OS=Plasmodium falciparum (isolate 3D7) GN=PFL1070c PE=1 SV=1	Q8I0V4_PLAF7	95 kDa	1.8	11	15	12	13	6	2	14.00%	15.10%	14.60%	11.90%	5.12%	2.92%
173	V-type H(+)-translocating pyrophosphatase, putative OS=Plasmodium falciparum (isolate 3D7) GN=PF14_0541 PE=3 SV=:	Q8IKR1_PLAF7	76 kDa	1.8	7	6	5	5	4	1	5.44%	5.58%	3.91%	5.58%	4.60%	1.26%
174	Phosphoglycerate mutase, putative OS=Plasmodium falciparum (isolate 3D7) GN=PF11_0208 PE=1 SV=1	Q8IIG6_PLAF7	29 kDa	1.8	3	3	3	2	0	2	18.00%	9.20%	9.20%	13.20%	1	8.80%
175	Hexose transporter, PfHT1 OS=Plasmodium falciparum (isolate 3D7) GN=HT1 PE=3 SV=1	Q7KWJ5_PLAF7	56 kDa	1.8	3	3	3	2	1	2	5.95%	4.96%	4.96%	4.96%	2.98%	5.95%
176	Spectrin beta chain, non-erythrocytic 2 OS=Homo sapiens GN=SPTBN2 PE=1 SV=3	SPTN2_HUMAN	271 kDa	1.8	10	13	9	6	11	0	1.05%	1.42%	0.71%	0.71%	3.39%	0.00%
177	High molecular weight rhoptry protein-2 OS=Plasmodium falciparum (isolate 3D7) GN=RhopH2 PE=4 SV=1	C0H571_PLAF7	163 kDa	1.8	46	28	26	38	13	6	22.90%	16.30%	17.40%	17.60%	9.00%	4.35%
178	Cluster of V-type proton ATPase catalytic subunit A OS=Plasmodium falciparum (isolate 3D7) GN=vapA PE=3 SV=1 (VATA	VATA_PLAF7	69 kDa	1.7	2	3	2	2	0	0	4.75%	6.55%	3.76%	3.76%	1	1
178.1	V-type proton ATPase catalytic subunit A OS=Plasmodium falciparum (isolate 3D7) GN=vapA PE=3 SV=1	VATA_PLAF7	69 kDa	1.7	2	3	2	2	0	0	4.75%	6.55%	3.76%	3.76%	1	1
178.2	V-type proton ATPase catalytic subunit A OS=Homo sapiens GN=ATP6V1A PE=1 SV=2	VATA_HUMAN	68 kDa	1	1	1	0	0	0	0	2.76%	2.76%	0.00%	0.00%	1	1
179	M17 leucyl aminopeptidase OS=Plasmodium falciparum (isolate 3D7) GN=LAP PE=1 SV=1	Q8IL11_PLAF7	68 kDa	1.7	2	2	3	2	1	0	5.79%	3.80%	5.45%	3.31%	1.98%	1
180	Nucleoside transporter 1 OS=Plasmodium falciparum (isolate 3D7) GN=nt1 PE=4 SV=1	Q8IDM6_PLAF7	48 kDa	1.7	1	4	2	2	1	0	4.27%	6.64%	6.40%	6.16%	1.90%	1
181	Uncharacterized protein OS=Plasmodium falciparum (isolate 3D7) GN=PF13_0219 PE=4 SV=1	Q8IDT3_PLAF7	114 kDa	1.7	3	2	2	0	0	2	3.35%	2.51%	2.51%	0.00%	1	2.51%
182	PIESP2 erythrocyte surface protein OS=Plasmodium falciparum (isolate 3D7) GN=PFE0060w PE=4 SV=1	Q8I488_PLAF7	49 kDa	1.7	3	1	3	2	0	0	6.37%	3.92%	8.58%	5.39%	0.00%	0.00%
183	Spermidine synthase OS=Plasmodium falciparum (isolate 3D7) GN=PF11_0301 PE=1 SV=1	Q8II73_PLAF7	37 kDa	1.7	3	2	2	2	0	0	7.79%	8.10%	4.36%	6.85%	1	1
184	60S ribosomal protein L23a, putative OS=Plasmodium falciparum (isolate 3D7) GN=PF13_0132 PE=1 SV=1	Q8IE82_PLAF7	22 kDa	1.7	3	2	2	1	2	1	13.20%	6.84%	13.20%	6.84%	6.84%	6.84%
185	Uncharacterized protein OS=Plasmodium falciparum (isolate 3D7) GN=PF10_0325 PE=1 SV=1	Q8IJ74_PLAF7	33 kDa	1.7	2	3	2	2	1	0	11.80%	15.60%	8.68%	3.82%	3.82%	1
186	Glyceraldehyde-3-phosphate dehydrogenase OS=Homo sapiens GN=GAPDH PE=1 SV=3	G3P_HUMAN	36 kDa	1.7	3	1	3	2	0	1	6.87%	4.48%	6.87%	6.87%	1	4.48%
187	40S ribosomal protein S16, putative OS=Plasmodium falciparum (isolate 3D7) GN=PF08_0076 PE=1 SV=1	Q8IAX5_PLAF7	16 kDa	1.7	2	4	1	2	1	0	16.00%	27.10%	8.33%	16.00%	5.56%	1
188	Plasmepsin II OS=Plasmodium falciparum (isolate 3D7) GN=PF14_0077 PE=3 SV=1	Q8I6V3_PLAF7	51 kDa	1.7	3	2	2	2	0	1	6.62%	5.52%	5.52%	5.52%	1	2.65%
189	Conserved Plasmodium membrane protein OS=Plasmodium falciparum (isolate 3D7) GN=PFL1825w PE=4 SV=1	Q8I546_PLAF7	24 kDa	1.7	9	7	10	9	5	0	10.50%	10.50%	10.50%	16.70%	12.40%	0.00%
190	Cluster of Beta-adducin OS=Homo sapiens GN=ADD2 PE=1 SV=3 (ADDB_HUMAN)	ADDB_HUMAN	81 kDa	1.7	9	9	8	11	3	0	13.00%	13.00%	13.00%	13.40%	3.72%	1
190.1	Beta-adducin OS=Homo sapiens GN=ADD2 PE=1 SV=3	ADDB_HUMAN	81 kDa	1.7	9	9	8	11	3	0	9.78%	9.78%	8.54%	13.40%	3.72%	1
190.2	Beta-adducin (Fragment) OS=Homo sapiens GN=ADD2 PE=1 SV=3	C9JTM0_HUMAN	37 kDa	1	0	0	0	0	0	0	1	1	1	1	1	1
191	Cluster of Heat shock 70 kDa protein OS=Plasmodium falciparum (isolate 3D7) GN=PF08_0054 PE=3 SV=1 (Q8IB24_PLAF	Q8IB24_PLAF7 [3]	74 kDa	1.7	28	21	23	24	13	5	23.80%	27.50%	21.70%	24.40%	14.30%	7.98%
191.1	Heat shock-related 70 kDa protein 2 OS=Homo sapiens GN=HSPA2 PE=1 SV=1	HSP72_HUMAN	70 kDa	2.1	6	3	6	4	2	1	6.26%	4.54%	8.14%	6.73%	3.76%	2.03%
191.2	Heat shock 70 kDa protein OS=Plasmodium falciparum (isolate 3D7) GN=PF08_0054 PE=3 SV=1	Q8IB24_PLAF7	74 kDa	1.8	25	18	20	18	12	5	22.00%	27.50%	21.70%	20.40%	14.20%	7.98%
191.3	Heat shock cognate 71 kDa protein (Fragment) OS=Homo sapiens GN=HSPA8 PE=1 SV=7	E9PK54_HUMAN	20 kDa	1.2	4	2	0	3	2	0	13.00%	14.10%	1	21.70%	13.00%	1
191.4	Heat shock cognate 71 kDa protein OS=Homo sapiens GN=HSPA8 PE=1 SV=1	E9PKE3_HUMAN (+1)	69 kDa	1	6	3	5	6	2	0	6.38%	4.63%	4.63%	11.50%	3.83%	1
191.5	Heat shock 70 kDa protein 6 OS=Homo sapiens GN=HSPA6 PE=1 SV=2	HSP76_HUMAN	71 kDa	1	0	0	0	3	4	0	1	1	1	4.20%	6.22%	1
191.6	Heat shock 70 kDa protein 1B OS=Homo sapiens GN=HSPA1B PE=1 SV=1	A0A0G2JIW1_HUMAN (+	70 kDa	1	0	0	0	4	0	0	1	1	1	6.54%	1	1
191.7	Heat shock 70 kDa protein 1-like OS=Homo sapiens GN=HSPA1L PE=1 SV=2	HS71L_HUMAN (+1)	70 kDa	1	0	0	0	0	0	0	1	1	1	1	1	1
191.8	Heat shock cognate 71 kDa protein (Fragment) OS=Homo sapiens GN=HSPA8 PE=1 SV=1	E9PN89_HUMAN	35 kDa	1	0	0	0	0	0	0	1	1	1	1	1	1
191.9	Heat shock cognate 71 kDa protein (Fragment) OS=Homo sapiens GN=HSPA8 PE=1 SV=1	E9PI65_HUMAN	18 kDa	1	0	0	0	4	2	0	1	1	1	24.40%	14.30%	1
192	Filaggrin-2 OS=Homo sapiens GN=FLG2 PE=1 SV=1	FILA2_HUMAN	248 kDa	1.7	1	2	2	1	1	0	0.96%	1.25%	2.26%	0.96%	0.71%	1
193	Rhoptry-associated protein 2, RAP2 OS=Plasmodium falciparum (isolate 3D7) GN=RAP2 PE=4 SV=1	Q8I484_PLAF7	47 kDa	1.7	4	2	4	3	2	0	9.05%	5.78%	9.05%	6.53%	5.28%	1
194	Casein kinase II, alpha subunit, putative OS=Plasmodium falciparum (isolate 3D7) GN=PF11_0096 PE=4 SV=1	Q8IIR9_PLAF7	40 kDa	1.7	1	3	1	1	1	0	3.28%	6.87%	3.28%	3.28%	2.69%	1
195	Uncharacterized protein OS=Plasmodium falciparum (isolate 3D7) GN=PF14_0657 PE=4 SV=1	Q8IKE8_PLAF7	97 kDa	1.7	1	3	1	1	0	0	1.35%	4.43%	1.35%	1.35%	1	0.00%
196	Proliferation-associated protein 2g4, putative OS=Plasmodium falciparum (isolate 3D7) GN=PF14_0261 PE=4 SV=1	Q8ILI2_PLAF7	43 kDa	1.7	1	3	1	1	1	0	2.92%	7.69%	2.92%	2.65%	2.65%	1
197	STI1-like protein OS=Plasmodium falciparum (isolate 3D7) GN=PF14_0324 PE=3 SV=1	STI1L_PLAF7	66 kDa	1.7	2	2	1	1	1	1	4.08%	3.90%	2.30%	1.77%	1.77%	1.77%
198	Dermcidin OS=Homo sapiens GN=DCD PE=1 SV=2	DCD_HUMAN	11 kDa	1.7	2	3	5	3	2	0	20.00%	22.70%	22.70%	12.70%	12.70%	1
199	Uncharacterized protein OS=Plasmodium falciparum (isolate 3D7) GN=PFD1170c PE=4 SV=1	Q8IFM0_PLAF7	36 kDa	1.7	1	1	3	0	0	0	3.88%	2.27%	9.06%	0.00%	1	1
200	DNAJ protein, putative OS=Plasmodium falciparum (isolate 3D7) GN=RESA PE=4 SV=1	Q8I0U6_PLAF7	127 kDa	1.7	2	2	1	1	0	1	2.30%	2.12%	1.11%	1.11%	1	1.11%
201	DNA-directed RNA polymerase OS=Plasmodium falciparum (isolate 3D7) GN=PFB0715w PE=3 SV=3	O96236_PLAF7	152 kDa	1.7	2	2	0	0	0	0	1.12%	2.01%	1	0.00%	0.00%	1
202	Clathrin heavy chain OS=Plasmodium falciparum (isolate 3D7) GN=PFL0930w PE=3 SV=1	Q8I5L6_PLAF7	233 kDa	1.7	3	1	0	0	0	0	1.70%	0.50%	1	1	0.00%	1
203	Conserved Plasmodium protein OS=Plasmodium falciparum (isolate 3D7) GN=PF10_0126 PE=4 SV=1	Q8IJR6_PLAF7	44 kDa	1.7	2	1	2	1	0	0	6.05%	2.37%	6.05%	2.37%	1	1
204	26S proteasome subunit, putative OS=Plasmodium falciparum (isolate 3D7) GN=PF14_0632 PE=4 SV=1	Q8IKH3_PLAF7	133 kDa	1.7	3	0	0	0	0	0	2.73%	1	0.00%	0.00%	0.00%	1
205	Proteasome subunit beta type OS=Plasmodium falciparum (isolate 3D7) GN=PF13_0156 PE=3 SV=1	Q8I6T3_PLAF7	30 kDa	1.7	1	2	2	0	0	0	4.44%	8.15%	4.44%	0.00%	1	1
206	Vacuolar ATP synthase subunit b OS=Plasmodium falciparum (isolate 3D7) GN=PFD0305c PE=3 SV=1	Q6ZMA8_PLAF7	56 kDa	1.7	2	2	1	1	0	0	5.47%	4.05%	3.04%	1.62%	0.00%	1
207	Endoplasmic reticulum-resident calcium binding protein OS=Plasmodium falciparum (isolate 3D7) GN=PF11_0098 PE=4 S	Q8IIR7_PLAF7	39 kDa	1.7	1	3	1	1	0	1	4.08%	12.50%	4.08%	4.08%	1	4.08%
208	Stearoyl-CoA Delta 9 desaturase, putative OS=Plasmodium falciparum (isolate 3D7) GN=PFE0555w PE=4 SV=1	Q8I0W9_PLAF7	114 kDa	1.7	1	2	2	1	0	0	1.16%	2.11%	2.11%	0.95%	1	1
209	Rhomboid protease ROM4 OS=Plasmodium falciparum (isolate 3D7) GN=ROM4 PE=3 SV=1	Q8I433_PLAF7	87 kDa	1.7	2	2	0	1	1	0	3.82%	3.82%	1	1.45%	1.45%	1
210	Triose phosphate transporter OS=Plasmodium falciparum (isolate 3D7) GN=PfiTPT PE=4 SV=1	Q8I0U9_PLAF7	60 kDa	1.7	1	2	2	0	0	0	2.48%	4.39%	2.48%	1	1	0.00%
**211**	**Uncharacterized protein OS=Plasmodium falciparum (isolate 3D7) GN=PF14_0266 PE=3 SV=1**	**Q8ILH7_PLAF7**	**51 kDa**	**1.7**	**0**	**1**	**3**	**0**	**0**	**0**	**1**	**2.54%**	**5.31%**	**1**	**1**	**1**
212	Probable cathepsin C OS=Plasmodium falciparum (isolate 3D7) GN=PF11_0174 PE=1 SV=1	CATC_PLAF7	80 kDa	1.7	0	1	3	0	0	0	0.00%	2.57%	4.29%	1	1	1
213	Beta3 proteasome subunit, putative OS=Plasmodium falciparum (isolate 3D7) GN=PFA_0400c PE=4 SV=1	Q8I261_PLAF7	23 kDa	1.7	3	0	1	0	0	0	11.30%	1	5.88%	0.00%	1	1
214	Splicing factor, putative OS=Plasmodium falciparum (isolate 3D7) GN=PFE0865c PE=4 SV=2	Q8I3T5_PLAF7	35 kDa	1.7	0	3	1	0	0	0	1	11.10%	4.36%	1	0.00%	1
215	Beta subunit of coatomer complex, putative OS=Plasmodium falciparum (isolate 3D7) GN=PFI0290c PE=4 SV=1	Q8I390_PLAF7	118 kDa	1.7	3	0	0	0	0	0	2.08%	1	1	1	0.00%	1

216	Uncharacterized protein OS=Plasmodium falciparum (isolate 3D7) GN=PF14_0567 PE=4 SV=1	Q8IKN7_PLAF7	40 kDa	1.7	2	0	2	1	1	0	7.35%	1	7.35%	3.53%	2.35%	1
217	Small ubiquitin-related modifier, putative OS=Plasmodium falciparum (isolate 3D7) GN=PfSUMO PE=4 SV=1	Q8I444_PLAF7	11 kDa	1.7	0	2	2	0	0	0	1	19.00%	19.00%	1	1	1
218	Plasmepsin IV OS=Plasmodium falciparum (isolate 3D7) GN=PF14_0075 PE=1 SV=1	Q8IM16_PLAF7	51 kDa	1.7	2	2	1	1	0	0	4.45%	4.68%	4.68%	4.68%	1	1
219	Cluster of Spectrin beta chain, erythrocytic OS=Homo sapiens GN=SPTB PE=1 SV=5 (SPTB1_HUMAN)	SPTB1_HUMAN	246 kDa	1.6	147	##	170	163	80	40	36.40%	37.70%	40.50%	39.20%	25.30%	15.30%
219.1	Spectrin beta chain, erythrocytic OS=Homo sapiens GN=SPTB PE=1 SV=5	SPTB1_HUMAN	246 kDa	1.6	147	##	170	163	80	40	36.40%	37.70%	40.50%	39.20%	25.30%	15.30%
219.2	Spectrin beta chain, non-erythrocytic 1 OS=Homo sapiens GN=SPTBN1 PE=1 SV=1	A0A087WUZ3_HUMAN	275 kDa	1	11	16	13	7	11	1	1.48%	1.86%	1.14%	1.14%	1.52%	0.42%
219.3	Spectrin beta chain, erythrocytic (Fragment) OS=Homo sapiens GN=SPTB PE=1 SV=1	H0YJE6_HUMAN	117 kDa	1	42	41	54	61	24	16	25.50%	22.90%	33.30%	30.00%	17.40%	13.70%
220	Mature parasite-infected erythrocyte surface antigen (MESA) or PfEMP2 OS=Plasmodium falciparum (isolate 3D7) GN=M	Q8I492_PLAF7	168 kDa	1.6	11	15	8	12	8	0	7.53%	9.97%	5.93%	9.14%	7.67%	1
221	DnaJ/SEC63 protein, putative OS=Plasmodium falciparum (isolate 3D7) GN=PF13_0102 PE=4 SV=1	Q8IEC8_PLAF7	76 kDa	1.6	3	2	3	3	0	0	5.22%	4.61%	6.30%	6.14%	1	1
222	60S ribosomal protein L17, putative OS=Plasmodium falciparum (isolate 3D7) GN=PF13_0268 PE=1 SV=1	Q8IDI5_PLAF7	23 kDa	1.6	2	4	2	3	1	0	3.94%	8.37%	3.94%	3.94%	3.94%	1
223	Uncharacterized protein OS=Plasmodium falciparum (isolate 3D7) GN=PF10_0028 PE=4 SV=1	Q8IK12_PLAF7	30 kDa	1.6	2	4	2	3	0	0	7.66%	13.30%	13.30%	13.30%	1	1
224	Solute carrier family 2, facilitated glucose transporter member 1 OS=Homo sapiens GN=SLC2A1 PE=1 SV=2	GTR1_HUMAN	54 kDa	1.6	7	7	8	8	4	2	7.32%	8.74%	10.40%	10.40%	8.54%	4.67%
225	Small GTP-binding protein sar1 OS=Plasmodium falciparum (isolate 3D7) GN=sar1 PE=3 SV=1	Q8I1S0_PLAF7	22 kDa	1.6	5	5	4	4	3	2	28.10%	32.30%	28.10%	22.40%	6.77%	12.50%
226	Heat shock protein 101, putative OS=Plasmodium falciparum (isolate 3D7) GN=PF11_0175 PE=1 SV=1	Q8IIJ8_PLAF7	103 kDa	1.5	7	8	5	6	6	0	9.38%	10.20%	6.95%	7.84%	7.28%	0.00%
227	Uncharacterized protein OS=Plasmodium falciparum (isolate 3D7) GN=PF10_0208 PE=4 SV=1	Q8IJI9_PLAF7	74 kDa	1.5	5	4	3	6	0	0	7.50%	6.70%	5.26%	8.93%	0.00%	1
228	Cluster of Sodium/potassium-transporting ATPase subunit alpha-3 OS=Homo sapiens GN=ATP1A3 PE=1 SV=1 (A0A0A0M	A0A0A0MT26_HUMAN	133 kDa	1.5	0	4	0	0	0	2	1	3.43%	0.00%	0.00%	0.00%	1.47%
228.1	Sodium/potassium-transporting ATPase subunit alpha-3 OS=Homo sapiens GN=ATP1A3 PE=1 SV=1	A0A0A0MT26_HUMAN	133 kDa	1.5	0	4	0	0	0	2	1	3.43%	0.00%	0.00%	0.00%	1.22%
228.2	Sodium/potassium-transporting ATPase subunit alpha-2 OS=Homo sapiens GN=ATP1A2 PE=1 SV=1	AT1A2_HUMAN (+1)	112 kDa	1	0	3	0	0	0	2	1	1.47%	0.00%	1	1	1.47%
228.3	Potassium-transporting ATPase alpha chain 1 OS=Homo sapiens GN=ATP4A PE=2 SV=5	ATP4A_HUMAN	114 kDa	1	0	0	0	0	0	0	1	1	1	1	0.00%	1
228.4	Sodium/potassium-transporting ATPase subunit alpha-1 OS=Homo sapiens GN=ATP1A1 PE=1 SV=1	AT1A1_HUMAN	113 kDa	1	0	3	0	0	0	2	1	1.47%	1	0.00%	1	1.47%
229	Cluster of Flotillin-1 OS=Homo sapiens GN=FLOT1 PE=1 SV=3 (FLOT1_HUMAN)	FLOT1_HUMAN	47 kDa	1.5	3	1	2	2	0	0	4.92%	2.58%	6.09%	7.56%	0.00%	1
229.1	Flotillin-1 OS=Homo sapiens GN=FLOT1 PE=1 SV=3	FLOT1_HUMAN	47 kDa	1.5	3	1	2	2	0	0	4.92%	2.58%	6.09%	6.56%	0.00%	1
229.2	Flotillin-1 (Fragment) OS=Homo sapiens GN=FLOT1 PE=1 SV=1	A2AB09_HUMAN	27 kDa	1	0	0	0	0	0	0	1	1	1	1	1	1
230	Uncharacterized protein OS=Plasmodium falciparum (isolate 3D7) GN=PFD0080c PE=4 SV=1	Q8I207_PLAF7	60 kDa	1.5	2	2	2	0	2	0	3.75%	3.75%	3.75%	0.00%	1.79%	1
231	3-oxo-5-alpha-steroid 4-dehydrogenase, putative OS=Plasmodium falciparum (isolate 3D7) GN=PF11_0370 PE=4 SV=2	Q8II05_PLAF7	35 kDa	1.5	3	2	1	2	0	0	7.09%	2.70%	2.70%	7.09%	1	1
232	Cluster of Hemoglobin subunit beta OS=Homo sapiens GN=HBB PE=1 SV=2 (HBB_HUMAN)	HBB_HUMAN [2]	16 kDa	1.5	27	27	22	24	16	11	61.90%	61.90%	61.20%	68.00%	59.20%	38.10%
232.1	Hemoglobin subunit delta OS=Homo sapiens GN=HBD PE=1 SV=2	HBD_HUMAN	16 kDa	1.7	15	17	13	15	9	2	49.00%	40.80%	48.30%	55.10%	38.80%	17.00%
232.2	Hemoglobin subunit beta OS=Homo sapiens GN=HBB PE=1 SV=2	HBB_HUMAN	16 kDa	1.5	23	25	18	21	14	10	61.90%	61.90%	61.20%	68.00%	59.20%	38.10%
232.3	Hemoglobin subunit gamma-2 OS=Homo sapiens GN=HBG2 PE=1 SV=1	E9PBW4_HUMAN (+1)	15 kDa	1	0	0	0	0	0	0	1	1	1	1	1	1
233	Cluster of Ankyrin-1 OS=Homo sapiens GN=ANK1 PE=1 SV=3 (ANK1_HUMAN)	ANK1_HUMAN [2]	206 kDa	1.5	94	76	75	101	41	23	25.70%	24.90%	23.40%	27.40%	14.90%	10.50%
233.1	Ankyrin-1 OS=Homo sapiens GN=ANK1 PE=1 SV=3	ANK1_HUMAN	206 kDa	1.5	94	76	75	101	39	22	25.70%	24.90%	23.40%	27.40%	14.90%	10.50%
233.2	Ankyrin-3 OS=Homo sapiens GN=ANK3 PE=1 SV=3	ANK3_HUMAN	480 kDa	1.1	7	3	6	7	5	3	0.46%	0.46%	1.07%	0.75%	1.28%	0.80%
233.3	Ankyrin-2 OS=Homo sapiens GN=ANK2 PE=1 SV=4	ANK2_HUMAN (+1)	434 kDa	1	7	3	4	6	2	0	0.51%	0.51%	0.51%	0.51%	0.48%	1
233.4	Ankyrin-3 (Fragment) OS=Homo sapiens GN=ANK3 PE=1 SV=1	A0A087WTF3_HUMAN	180 kDa	1	0	0	0	0	0	0	1	1	1	1	1	1
233.5	Ankyrin-2 (Fragment) OS=Homo sapiens GN=ANK2 PE=1 SV=1	D6RHE1_HUMAN	176 kDa	1	0	0	0	0	0	0	1	1	1	1	1	1
233.6	Ankyrin-3 (Fragment) OS=Homo sapiens GN=ANK3 PE=1 SV=1	A0A087WZ65_HUMAN	109 kDa	1	0	0	0	0	0	0	1	1	1	1	1	1
233.7	Ankyrin-2 (Fragment) OS=Homo sapiens GN=ANK2 PE=1 SV=1	E9PHW9_HUMAN	189 kDa	1	0	0	0	0	0	0	1	1	1	1	1	1
234	Hemoglobin subunit alpha OS=Homo sapiens GN=HBA1 PE=1 SV=2	HBA_HUMAN	15 kDa	1.5	15	15	11	14	9	5	48.60%	36.60%	36.60%	31.00%	23.20%	31.00%
235	60S ribosomal protein L13, putative OS=Plasmodium falciparum (isolate 3D7) GN=PF10_0043 PE=1 SV=1	Q8IJZ7_PLAF7	24 kDa	1.4	5	3	5	4	3	2	15.30%	10.90%	18.80%	15.30%	5.94%	10.90%
236	Protein 4.1 OS=Homo sapiens GN=EPB41 PE=1 SV=4	41_HUMAN	97 kDa	1.4	19	16	20	23	12	4	19.60%	15.50%	15.50%	19.90%	12.30%	6.37%
237	Carbonic anhydrase 1 OS=Homo sapiens GN=CA1 PE=1 SV=2	CAH1_HUMAN	29 kDa	1.4	7	3	4	8	0	1	28.70%	10.30%	19.20%	31.80%	1	6.13%
238	Uncharacterized protein OS=Plasmodium falciparum (isolate 3D7) GN=PFE1605w PE=4 SV=1	Q8I3F0_PLAF7	61 kDa	1.4	3	2	2	3	0	0	6.63%	3.98%	3.79%	4.92%	0.00%	1
239	Sec61 alpha subunit, PfSec61 OS=Plasmodium falciparum (isolate 3D7) GN=Sec61 PE=3 SV=1	Q8IDN6_PLAF7	52 kDa	1.4	2	3	2	3	0	0	2.54%	6.78%	4.45%	4.87%	1	1
240	Uncharacterized protein OS=Plasmodium falciparum (isolate 3D7) GN=PFI1590c PE=4 SV=1	Q8I2I8_PLAF7	159 kDa	1.4	6	2	3	3	0	4	5.22%	2.01%	3.58%	2.76%	1	2.68%
241	Cluster of Pre-mRNA splicing factor, putative OS=Plasmodium falciparum (isolate 3D7) GN=PFD0265w PE=4 SV=1 (Q8I1X	Q8I1X5_PLAF7	366 kDa	1.3	2	1	0	0	1	1	0.79%	0.29%	0.00%	0.00%	0.32%	0.70%
241.1	Pre-mRNA splicing factor, putative OS=Plasmodium falciparum (isolate 3D7) GN=PFD0265w PE=4 SV=1	Q8I1X5_PLAF7	366 kDa	1.3	2	1	0	0	1	1	0.57%	0.29%	0.00%	0.00%	0.32%	0.70%
241.2	Pre-mRNA-processing-splicing factor 8 (Fragment) OS=Homo sapiens GN=PRPF8 PE=1 SV=7	I3L0J9_HUMAN	120 kDa	1	1	0	0	0	0	0	0.79%	1	0.00%	1	1	0.00%
241.3	Pre-mRNA-processing-splicing factor 8 OS=Homo sapiens GN=PRPF8 PE=1 SV=2	PRP8_HUMAN	274 kDa	1	0	0	0	0	0	0	1	1	1	1	1	1
242	Uncharacterized protein OS=Plasmodium falciparum (isolate 3D7) GN=PF14_0649 PE=4 SV=1	Q8IKF6_PLAF7	296 kDa	1.3	0	2	0	0	0	0	0.00%	1.09%	0.00%	0.00%	0.00%	0.00%
243	DEAD/DEAH box helicase, putative OS=Plasmodium falciparum (isolate 3D7) GN=PFI0165c PE=4 SV=1	Q8I3B4_PLAF7	301 kDa	1.3	1	2	0	0	0	0	0.36%	0.83%	0.00%	1	0.00%	1
244	Asparagine-rich antigen OS=Plasmodium falciparum (isolate 3D7) GN=PF11_0111 PE=4 SV=1	Q8IIQ7_PLAF7	174 kDa	1.3	1	0	2	0	0	0	0.74%	0.00%	1.48%	0.00%	0.00%	1
245	Cluster of 55 kDa erythrocyte membrane protein OS=Homo sapiens GN=MPP1 PE=1 SV=2 (EM55_HUMAN)	EM55_HUMAN	52 kDa	1.3	2	2	4	4	0	0	4.51%	5.15%	7.08%	10.90%	1	1
245.1	55 kDa erythrocyte membrane protein OS=Homo sapiens GN=MPP1 PE=1 SV=2	EM55_HUMAN	52 kDa	1.3	2	2	4	4	0	0	4.51%	5.15%	7.08%	10.90%	1	1
245.2	55 kDa erythrocyte membrane protein (Fragment) OS=Homo sapiens GN=MPP1 PE=1 SV=1	A8MTH1_HUMAN	27 kDa	1	0	0	0	0	0	0	1	1	1	1	1	1
246	Protein dopey homolog PFC0245c OS=Plasmodium falciparum (isolate 3D7) GN=PFC0245c PE=2 SV=1	DOP1_PLAF7	468 kDa	1.3	2	0	0	0	0	0	0.53%	0.00%	0.00%	0.00%	0.00%	1
247	Acyl-CoA synthetase, PfACS12 OS=Plasmodium falciparum (isolate 3D7) GN=PfACS12 PE=4 SV=1	C6KT35_PLAF7	164 kDa	1.3	2	1	1	0	1	0	1.51%	0.79%	0.79%	1	0.79%	1
248	U5 small nuclear ribonucleoprotein-specific protein, putative OS=Plasmodium falciparum (isolate 3D7) GN=PFD1060w PE	Q8IFP1_PLAF7	338 kDa	1.3	2	1	0	0	0	0	0.84%	0.38%	0.00%	0.00%	0.00%	1
249	Uncharacterized protein OS=Plasmodium falciparum (isolate 3D7) GN=MAL8P1.73 PE=4 SV=1	C0H4V4_PLAF7	134 kDa	1.3	2	0	0	0	0	0	1.64%	1	0.00%	0.00%	0.00%	0.00%
250	Uncharacterized protein OS=Plasmodium falciparum (isolate 3D7) GN=PF08_0137 PE=4 SV=1	Q8IAK9_PLAF7	147 kDa	1.3	0	1	2	0	0	0	0.00%	0.90%	1.72%	0.00%	0.00%	1
251	Uncharacterized protein OS=Plasmodium falciparum (isolate 3D7) GN=PFL0340w PE=4 SV=1	Q8I5X7_PLAF7	86 kDa	1.3	1	2	1	0	0	1	1.24%	3.30%	1.24%	0.00%	1	1.93%
252	60S ribosomal protein L35Ae, putative OS=Plasmodium falciparum (isolate 3D7) GN=PF11_0438 PE=1 SV=1	Q8IHT9_PLAF7	16 kDa	1.3	2	1	1	1	0	0	15.00%	8.57%	8.57%	8.57%	1	1
253	Uncharacterized protein OS=Plasmodium falciparum (isolate 3D7) GN=PF14_0546 PE=4 SV=1	Q8IKQ7_PLAF7	76 kDa	1.3	2	0	0	0	0	0	2.66%	0.00%	0.00%	1	1	1
254	Serine-repeat antigen protein OS=Plasmodium falciparum (isolate 3D7) GN=SERA PE=1 SV=1	SERA_PLAF7	112 kDa	1.3	1	2	1	1	0	0	1.10%	2.91%	1.10%	1.10%	1	1
255	Uncharacterized protein OS=Plasmodium falciparum (isolate 3D7) GN=MAL8P1.53 PE=4 SV=1	Q8IB44_PLAF7	61 kDa	1.3	1	2	1	0	0	0	1.75%	3.89%	2.14%	0.00%	1	1
256	Leucyl tRNA synthase OS=Plasmodium falciparum (isolate 3D7) GN=PFF1095w PE=3 SV=1	C6KT64_PLAF7	170 kDa	1.3	1	2	0	0	0	0	0.83%	2.07%	1	0.00%	1	1
257	Myosin-A OS=Plasmodium falciparum (isolate 3D7) GN=PF13_0233 PE=1 SV=1	MYOA_PLAF7	92 kDa	1.3	2	0	0	1	1	0	3.42%	0.00%	1	1.71%	1.71%	1
258	Uncharacterized protein OS=Plasmodium falciparum (isolate 3D7) GN=PF08_0035 PE=4 SV=1	Q8IB63_PLAF7	138 kDa	1.3	2	0	0	1	1	0	2.30%	0.00%	1	0.68%	2.81%	1
259	Aspartate carbamoyltransferase OS=Plasmodium falciparum (isolate 3D7) GN=atcasE PE=3 SV=1	Q8IDP8_PLAF7	43 kDa	1.3	2	1	0	0	0	1	5.87%	2.93%	1	1	1	2.93%
260	Proliferating cell nuclear antigen OS=Plasmodium falciparum (isolate 3D7) GN=PCNA PE=3 SV=1	PCNA_PLAF7	31 kDa	1.3	1	2	0	1	0	0	4.74%	9.85%	1	4.01%	1	0.00%
261	WD-repeat protein, putative OS=Plasmodium falciparum (isolate 3D7) GN=PF08_0130 PE=4 SV=1	Q8IAM3_PLAF7	129 kDa	1.3	0	2	1	0	0	1	1	2.94%	0.98%	0.00%	1	0.98%
262	tRNA binding protein, putative OS=Plasmodium falciparum (isolate 3D7) GN=PF14_0401 PE=4 SV=1	Q8IL48_PLAF7	46 kDa	1.3	1	2	1	0	0	0	2.49%	4.73%	2.49%	1	1	1
263	Uncharacterized protein OS=Plasmodium falciparum (isolate 3D7) GN=MAL7P1.202 PE=4 SV=1	C0H4L1_PLAF7	146 kDa	1.3	2	1	0	0	0	0	1.95%	0.98%	0.00%	0.00%	1	1
264	Ribonucleotide reductase small subunit, putative OS=Plasmodium falciparum (isolate 3D7) GN=PF10_0154 PE=4 SV=2	Q8IJN8_PLAF7	40 kDa	1.3	1	2	1	0	0	0	2.69%	5.97%	2.69%	0.00%	1	1
265	Nucleosome assembly protein 1, putative OS=Plasmodium falciparum (isolate 3D7) GN=PFL0185c PE=1 SV=2	Q8I608_PLAF7	40 kDa	1.3	1	2	1	1	0	0	3.17%	6.05%	3.17%	3.17%	1	1
266	Cluster of 40S ribosomal protein S26e, putative OS=Plasmodium falciparum (isolate 3D7) GN=PFB0830w PE=1 SV=1 (O96	O96258_PLAF7	13 kDa	1.3	1	2	1	0	0	0	11.20%	17.80%	11.20%	1	1	1
266.1	40S ribosomal protein S26e, putative OS=Plasmodium falciparum (isolate 3D7) GN=PFB0830w PE=1 SV=1	O96258_PLAF7	13 kDa	1.3	1	2	1	0	0	0	11.20%	17.80%	11.20%	1	1	1
266.2	Aminoacyl tRNA synthase complex-interacting multifunctional protein 2 OS=Homo sapiens GN=AIMP2 PE=1 SV=2	AIMP2_HUMAN-DECOY (	?	1	0	0	0	0	0	0	1	1	1	1	1	1
267	ATP synthase (C/AC39) subunit, putative OS=Plasmodium falciparum (isolate 3D7) GN=PF14_0615 PE=4 SV=1	Q8IKJ0_PLAF7	45 kDa	1.3	2	1	0	0	0	0	5.24%	2.36%	1	1	1	1
268	Bifunctional glucose-6-phosphate 1-dehydrogenase/6-phosphogluconolactonase OS=Plasmodium falciparum (isolate 3D	GLUPH_PLAF7	107 kDa	1.3	2	1	0	0	0	0	2.42%	1.21%	1	1	1	1
269	60S ribosomal protein L14, putative OS=Plasmodium falciparum (isolate 3D7) GN=PF14_0296 PE=1 SV=1	Q8ILE8_PLAF7	19 kDa	1.3	2	1	1	1	0	1	13.90%	7.88%	7.88%	7.88%	1	7.88%
270	Uncharacterized protein OS=Plasmodium falciparum (isolate 3D7) GN=PF14_0092 PE=4 SV=2	Q8IM01_PLAF7	31 kDa	1.3	2	1	1	0	0	0	10.70%	6.49%	6.49%	0.00%	1	1
271	Ethanolaminephosphotransferase, putative OS=Plasmodium falciparum (isolate 3D7) GN=PFF1375c PE=3 SV=1	C6KTB9_PLAF7	45 kDa	1.3	1	2	1	1	0	0	2.05%	3.84%	2.05%	2.05%	1	1
272	Conserved protein OS=Plasmodium falciparum (isolate 3D7) GN=PF11_0287 PE=4 SV=2	Q8II87_PLAF7	35 kDa	1.3	2	1	1	0	0	0	8.08%	3.37%	4.04%	1	1	1
273	60S ribosomal protein L23, putative OS=Plasmodium falciparum (isolate 3D7) GN=PF13_0171 PE=1 SV=1	Q8IE09_PLAF7	15 kDa	1.3	2	1	1	1	1	0	18.00%	10.80%	10.80%	7.19%	7.19%	1
274	Glutamate dehydrogenase OS=Plasmodium falciparum (isolate 3D7) GN=PF14_0164 PE=3 SV=1	Q8ILT0_PLAF7	53 kDa	1.3	2	1	1	0	0	0	4.89%	2.34%	2.34%	1	1	1
275	Uncharacterized protein OS=Plasmodium falciparum (isolate 3D7) GN=MAL13P1.50 PE=4 SV=1	Q8IEL0_PLAF7	24 kDa	1.3	1	2	1	1	1	0	8.29%	12.20%	8.29%	8.29%	8.29%	1
276	Oxidoreductase, short-chain dehydrogenase family, putative OS=Plasmodium falciparum (isolate 3D7) GN=PFF1265w PE	C6KT97_PLAF7	45 kDa	1.3	1	2	0	0	0	0	2.61%	5.22%	1	1	1	1
277	RNA binding protein, putative OS=Plasmodium falciparum (isolate 3D7) GN=PFI1175c PE=4 SV=1	Q8I2R8_PLAF7	23 kDa	1.3	1	2	1	0	0	0	5.45%	14.40%	5.45%	1	1	1
278	40S ribosomal protein S12 OS=Plasmodium falciparum (isolate 3D7) GN=RPS12 PE=1 SV=1	RS12_PLAF7	15 kDa	1.3	1	2	1	1	0	0	7.80%	16.30%	8.51%	7.80%	1	1
**279**	**Uncharacterized protein OS=Plasmodium falciparum (isolate 3D7) GN=PF14_0574 PE=4 SV=1**	**Q8IKN0_PLAF7**	**38 kDa**	**1.3**	**0**	**2**	**1**	**0**	**0**	**0**	**1**	**8.31%**	**4.62%**	**1**	**1**	**1**
280	Chromatin assembly factor 1 protein WD40 domain, putative OS=Plasmodium falciparum (isolate 3D7) GN=PFA_0520c P	Q8I238_PLAF7	51 kDa	1.3	2	0	1	0	0	0	6.73%	1	2.69%	1	1	1
281	cGMP-dependent protein kinase OS=Plasmodium falciparum (isolate 3D7) GN=PKG PE=1 SV=1	Q8I719_PLAF7	98 kDa	1.3	2	0	0	0	0	0	2.58%	0.00%	1	1	1	1
282	Doublecortin domain-containing protein 2 OS=Homo sapiens GN=DCDC2 PE=1 SV=2	DCDC2_HUMAN-DECOY	?	1.3	0	1	2	0	1	0	1	0.00%	0.00%	1	0.00%	1
283	Golgi-associated plant pathogenesis-related protein 1 OS=Homo sapiens GN=GLIPR2 PE=1 SV=3	GAPR1_HUMAN (+1)	17 kDa	1.3	2	1	0	1	1	0	16.90%	8.44%	1	8.44%	8.44%	1
284	Hemolysin, putative OS=Plasmodium falciparum (isolate 3D7) GN=PF14_0528 PE=4 SV=2	Q8IKS4_PLAF7	33 kDa	1.3	1	2	0	0	0	0	3.90%	6.74%	1	1	1	1
285	Desmocollin-1 OS=Homo sapiens GN=DSC1 PE=1 SV=2	DSC1_HUMAN	100 kDa	1.3	0	0	2	1	1	0	1	1	3.24%	1.12%	1.57%	1
286	Inosine-5'-monophosphate dehydrogenase OS=Plasmodium falciparum (isolate 3D7) GN=PFI1020c PE=3 SV=1	Q8I2U5_PLAF7	56 kDa	1.3	1	2	1	0	0	1	2.94%	5.10%	2.94%	1	1	2.16%
287	Fatty acid-binding protein, epidermal OS=Homo sapiens GN=FABP5 PE=1 SV=3	FABP5_HUMAN	15 kDa	1.3	0	0	2	0	0	0	1	0.00%	13.30%	1	1	1
288	Hydroxyethylthiazole kinase, putative OS=Plasmodium falciparum (isolate 3D7) GN=PFL1920c PE=4 SV=1	Q8I527_PLAF7	34 kDa	1.3	0	2	1	0	0	1	1	10.30%	3.64%	1	1	6.62%
289	Proteasome subunit beta type OS=Plasmodium falciparum (isolate 3D7) GN=PF14_0676 PE=3 SV=2	Q8IKC9_PLAF7	23 kDa	1.3	1	2	0	0	0	0	4.10%	12.30%	1	1	1	1
290	Cluster of Clathrin heavy chain 1 OS=Homo sapiens GN=CLTC PE=1 SV=5 (CLH1_HUMAN)	CLH1_HUMAN	192 kDa	1.3	0	2	0	0	0	0	1	17.60%	1	1	0.00%	1
290.1	Clathrin heavy chain 1 OS=Homo sapiens GN=CLTC PE=1 SV=5	CLH1_HUMAN	192 kDa	1.3	0	2	0	0	0	0	1	2.09%	1	1	0.00%	1
290.2	Clathrin heavy chain 1 (Fragment) OS=Homo sapiens GN=CLTC PE=1 SV=1	J3KRF5_HUMAN	16 kDa	1	0	1	0	0	0	0	1	17.60%	1	1	1	1
291	ATP-binding cassette sub-family B member 6, mitochondrial OS=Homo sapiens GN=ABCB6 PE=1 SV=1	ABCB6_HUMAN (+1)	94 kDa	1.3	2	0	0	0	0	0	3.09%	1	1	1	1	1
292	Proteasome subunit alpha type 2, putative OS=Plasmodium falciparum (isolate 3D7) GN=PFF0420c PE=4 SV=1	C6KST3_PLAF7	27 kDa	1.3	0	1	2	0	0	1	1	4.68%	8.94%	1	1	5.53%
293	Uncharacterized protein OS=Plasmodium falciparum (isolate 3D7) GN=MAL13P1.61 PE=4 SV=1	Q8IEJ0_PLAF7	28 kDa	1.3	1	2	0	0	0	0	4.49%	13.90%	1	1	1	1
294	Uncharacterized protein OS=Plasmodium falciparum (isolate 3D7) GN=PF14_0714 PE=4 SV=1	Q8IK92_PLAF7	46 kDa	1.3	0	2	1	0	0	0	1	5.58%	3.05%	0.00%	1	1
295	Calmodulin-like protein 5 OS=Homo sapiens GN=CALML5 PE=1 SV=2	CALL5_HUMAN	16 kDa	1.3	0	2	0	0	0	0	1	13.00%	1	1	1	1
296	Proteasome component C8, putative OS=Plasmodium falciparum (isolate 3D7) GN=PFC0745c PE=4 SV=1	O77396_PLAF7	29 kDa	1.3	0	1	2	0	0	0	1	3.17%	11.10%	1	1	1
297	60S ribosomal protein L10, putative OS=Plasmodium falciparum (isolate 3D7) GN=PF14_0141 PE=1 SV=1	Q8ILV2_PLAF7	25 kDa	1.3	1	2	0	0	0	0	3.65%	8.68%	1	1	1	1
298	GTP-binding nuclear protein Ran OS=Homo sapiens GN=RAN PE=1 SV=3	B5MDF5_HUMAN (+3)	26 kDa	1.3	2	0	0	0	0	0	7.73%	1	1	1	1	1
299	Keratin, type I cytoskeletal 9 OS=Homo sapiens GN=KRT9 PE=1 SV=3	K1C9_HUMAN	62 kDa	1.3	23	30	43	36	32	5	31.80%	36.40%	52.80%	51.40%	44.30%	13.80%
300	60S ribosomal protein L34-A, putative OS=Plasmodium falciparum (isolate 3D7) GN=PF07_0043 PE=1 SV=1	Q8IBY4_PLAF7	17 kDa	1.3	4	0	4	2	4	0	11.30%	0.00%	16.00%	6.00%	11.30%	0.00%

301	Cluster of Keratin, type II cytoskeletal 1 OS=Homo sapiens GN=KRT1 PE=1 SV=6 (K2C1_HUMAN)	K2C1_HUMAN [7]	66 kDa	1.3	75	82	114	117	74	20	41.80%	41.00%	43.50%	42.40%	37.70%	14.10%
301.1	Keratin, type II cytoskeletal 1 OS=Homo sapiens GN=KRT1 PE=1 SV=6	K2C1_HUMAN	66 kDa	1.4	42	44	59	62	35	10	41.80%	41.00%	43.50%	42.40%	37.70%	13.00%
301.2	Keratin, type II cytoskeletal 6B OS=Homo sapiens GN=KRT6B PE=1 SV=5	K2C6B_HUMAN	60 kDa	1.3	11	10	15	16	9	3	17.00%	14.90%	22.30%	22.50%	13.50%	5.14%
301.3	Keratin, type II cytoskeletal 2 epidermal OS=Homo sapiens GN=KRT2 PE=1 SV=2	K22E_HUMAN	65 kDa	1.2	18	24	33	33	21	8	29.10%	30.40%	29.70%	40.10%	26.90%	14.10%
301.4	Keratin, type II cytoskeletal 6A OS=Homo sapiens GN=KRT6A PE=1 SV=3	K2C6A_HUMAN	60 kDa	1.2	12	10	16	21	9	3	20.00%	16.10%	24.10%	27.30%	15.20%	6.38%
301.5	Keratin, type II cytoskeletal 5 OS=Homo sapiens GN=KRT5 PE=1 SV=3	K2C5_HUMAN	62 kDa	1.1	10	9	18	15	15	3	15.80%	17.50%	21.70%	19.50%	22.40%	7.97%
301.6	Keratin, type II cytoskeletal 79 OS=Homo sapiens GN=KRT79 PE=1 SV=2	K2C79_HUMAN	58 kDa	1.1	2	3	5	5	3	0	3.36%	3.93%	3.93%	5.42%	6.92%	1
301.7	Keratin, type II cuticular Hb4 OS=Homo sapiens GN=KRT84 PE=2 SV=2	KRT84_HUMAN	65 kDa	1	1	1	0	2	0	0	1.50%	1.50%	1	1.50%	1	0.00%
301.8	Keratin, type II cytoskeletal 75 OS=Homo sapiens GN=KRT75 PE=1 SV=2	K2C75_HUMAN	60 kDa	1	0	0	0	5	5	0	1	1	1	5.26%	7.08%	1
301.9	Keratin, type II cytoskeletal 72 OS=Homo sapiens GN=KRT72 PE=1 SV=2	K2C72_HUMAN	56 kDa	1	0	0	0	2	0	0	1	1	1	1.76%	1	0.00%
301.1	Keratin, type II cytoskeletal 3 OS=Homo sapiens GN=KRT3 PE=1 SV=3	K2C3_HUMAN	64 kDa	1	0	3	0	5	0	0	1	3.34%	1	5.25%	1	1
301.11	Keratin, type II cytoskeletal 1b OS=Homo sapiens GN=KRT77 PE=2 SV=3	K2C1B_HUMAN	62 kDa	1	0	0	0	0	0	0	1	1	1	1	1	1
301.12	Keratin, type II cytoskeletal 7 OS=Homo sapiens GN=KRT7 PE=1 SV=5	K2C7_HUMAN	51 kDa	1	0	0	0	0	0	0	1	1	1	1	1	1
301.13	Keratin, type II cytoskeletal 8 OS=Homo sapiens GN=KRT8 PE=1 SV=7	K2C8_HUMAN	54 kDa	1	2	1	0	0	0	0	3.93%	1.86%	1	1	1	1
301.14	Keratin, type II cytoskeletal 71 OS=Homo sapiens GN=KRT71 PE=1 SV=3	K2C71_HUMAN	57 kDa	1	0	1	0	0	0	0	1	1.72%	1	1	1	1
301.15	Keratin, type II cytoskeletal 73 OS=Homo sapiens GN=KRT73 PE=1 SV=1	K2C73_HUMAN	59 kDa	1	0	0	0	0	2	0	1	1	1	1	3.89%	1
301.16	Keratin, type II cytoskeletal 5 (Fragment) OS=Homo sapiens GN=KRT5 PE=1 SV=7	F8W0C6_HUMAN	21 kDa	1	0	0	8	0	5	0	1	1	27.90%	1	22.30%	1
301.17	Keratin, type II cytoskeletal 4 OS=Homo sapiens GN=KRT4 PE=1 SV=4	K2C4_HUMAN	57 kDa	1	0	0	0	0	1	0	1	1	1	1	2.25%	1
301.18	Keratin, type II cytoskeletal 74 OS=Homo sapiens GN=KRT74 PE=1 SV=1	F8W1S1_HUMAN (+1)	59 kDa	1	0	0	2	0	0	0	1	1	1.66%	1	1	1
301.19	Keratin, type II cytoskeletal 8 (Fragment) OS=Homo sapiens GN=KRT8 PE=1 SV=1	F8W1U3_HUMAN	33 kDa	1	0	0	0	0	0	0	1	1	1	1	1	1
301.2	Keratin, type II cytoskeletal 2 oral OS=Homo sapiens GN=KRT76 PE=1 SV=2	K22O_HUMAN	66 kDa	0.8	2	3	0	6	0	0	2.82%	3.29%	1	7.21%	1	1
302	Cluster of Keratin, type I cytoskeletal 10 OS=Homo sapiens GN=KRT10 PE=1 SV=6 (K1C10_HUMAN)	K1C10_HUMAN [6]	59 kDa	1.3	38	54	63	60	50	11	37.00%	38.90%	37.00%	35.30%	31.50%	13.60%
302.1	Keratin, type I cytoskeletal 16 OS=Homo sapiens GN=KRT16 PE=1 SV=4	K1C16_HUMAN	51 kDa	1.6	15	21	23	22	14	1	29.00%	38.90%	36.60%	35.30%	24.50%	2.33%
302.2	Keratin, type I cytoskeletal 14 OS=Homo sapiens GN=KRT14 PE=1 SV=4	K1C14_HUMAN	52 kDa	1.3	12	16	17	16	15	4	24.80%	34.30%	30.90%	24.80%	22.50%	13.60%
302.3	Keratin, type I cytoskeletal 12 OS=Homo sapiens GN=KRT12 PE=1 SV=1	K1C12_HUMAN	54 kDa	1.2	3	0	1	2	1	0	5.87%	1	1.82%	1.82%	1.82%	1
302.4	Keratin, type I cytoskeletal 10 OS=Homo sapiens GN=KRT10 PE=1 SV=6	K1C10_HUMAN	59 kDa	1.2	17	27	30	30	24	7	22.10%	30.30%	26.40%	25.00%	25.30%	13.50%
302.5	Keratin, type I cytoskeletal 17 OS=Homo sapiens GN=KRT17 PE=1 SV=2	K1C17_HUMAN	48 kDa	1.1	8	9	9	9	12	2	14.60%	14.40%	15.50%	14.80%	20.60%	4.86%
302.6	Keratin, type I cuticular Ha5 OS=Homo sapiens GN=KRT35 PE=2 SV=5	KRT35_HUMAN	50 kDa	1	0	0	0	0	0	0	0.00%	1	0.00%	1	0.00%	0.00%
302.7	Keratin, type I cytoskeletal 28 OS=Homo sapiens GN=KRT28 PE=1 SV=2	K1C28_HUMAN	51 kDa	1	0	0	4	0	2	0	1	1	4.09%	1	2.16%	1
302.8	Keratin, type I cytoskeletal 20 OS=Homo sapiens GN=KRT20 PE=1 SV=1	K1C20_HUMAN	48 kDa	1	0	1	0	0	0	0	1	2.12%	1	1	1	1
302.9	Keratin, type I cytoskeletal 18 OS=Homo sapiens GN=KRT18 PE=1 SV=1	F8VZY9_HUMAN (+1)	44 kDa	1	0	0	0	0	0	0	1	1	1	1	0.00%	1
302.1	Keratin, type I cytoskeletal 15 OS=Homo sapiens GN=KRT15 PE=1 SV=3	K1C15_HUMAN	49 kDa	1	0	0	0	0	0	1	1	1	1	1	1	2.41%
302.11	Keratin, type I cytoskeletal 13 OS=Homo sapiens GN=KRT13 PE=1 SV=4	K1C13_HUMAN (+1)	50 kDa	1	0	0	0	0	0	0	1	1	1	1	1	1
302.12	Keratin, type I cuticular Ha2 OS=Homo sapiens GN=KRT32 PE=2 SV=3	K1H2_HUMAN	50 kDa	1	0	0	0	0	0	0	1	1	1	1	1	1
302.13	Keratin-like protein KRT222 OS=Homo sapiens GN=KRT222 PE=2 SV=1	KT222_HUMAN	34 kDa	1	0	0	0	0	0	0	1	1	1	1	1	1
302.14	Keratin, type I cytoskeletal 27 OS=Homo sapiens GN=KRT27 PE=1 SV=2	K1C27_HUMAN	50 kDa	1	0	0	3	0	3	0	1	1	3.92%	1	4.36%	1
302.15	Keratin, type I cytoskeletal 16 (Fragment) OS=Homo sapiens GN=KRT16 PE=1 SV=1	K7ENW6_HUMAN	19 kDa	1	0	0	0	0	0	0	1	1	1	1	1	1
302.16	Keratin, type I cytoskeletal 19 OS=Homo sapiens GN=KRT19 PE=1 SV=4	K1C19_HUMAN	44 kDa	1	0	0	0	0	0	0	1	1	1	1	1	1
302.17	Keratin, type I cuticular Ha3-II OS=Homo sapiens GN=KRT33B PE=1 SV=3	KT33B_HUMAN	46 kDa	1	0	0	0	0	0	0	1	1	1	1	1	1
302.18	Keratin, type I cuticular Ha7 OS=Homo sapiens GN=KRT37 PE=3 SV=3	KRT37_HUMAN (+1)	50 kDa	1	0	0	0	0	0	0	1	1	1	1	1	1
302.19	Keratin, type I cytoskeletal 24 OS=Homo sapiens GN=KRT24 PE=1 SV=1	K1C24_HUMAN	55 kDa	0.4	0	0	0	3	3	0	1	1	1	5.52%	2.10%	1
303	60S ribosomal protein L5, putative OS=Plasmodium falciparum (isolate 3D7) GN=PF14_0230 PE=1 SV=1	Q8ILL3_PLAF7	34 kDa	1.2	2	1	2	1	1	2	2.72%	3.06%	10.50%	3.06%	3.06%	6.12%
304	DNAJ protein, putative OS=Plasmodium falciparum (isolate 3D7) GN=PF08_0032 PE=4 SV=1	Q8IB72_PLAF7	77 kDa	1.2	2	1	2	2	0	0	3.66%	1.98%	1.53%	3.66%	0.00%	1
305	60S ribosomal protein L19, putative OS=Plasmodium falciparum (isolate 3D7) GN=PFF0700c PE=1 SV=1	C6KSY6_PLAF7	22 kDa	1.2	3	1	1	1	2	1	18.70%	9.34%	4.95%	4.40%	9.34%	4.95%
306	Uncharacterized protein OS=Plasmodium falciparum (isolate 3D7) GN=PF14_0186 PE=4 SV=2	Q8ILQ8_PLAF7	80 kDa	1.2	2	2	1	1	2	0	3.58%	3.58%	1.94%	2.09%	3.58%	1
307	Merozoite capping protein 1 OS=Plasmodium falciparum (isolate 3D7) GN=PF10_0268 PE=4 SV=1	Q8IJD0_PLAF7	44 kDa	1.2	2	4	5	6	2	1	4.83%	8.91%	15.30%	15.00%	6.36%	4.07%
308	60S ribosomal protein L11a, putative OS=Plasmodium falciparum (isolate 3D7) GN=PF07_0079 PE=1 SV=1	Q8IBQ6_PLAF7	20 kDa	1.2	2	2	2	2	2	1	12.10%	12.10%	8.09%	8.09%	8.09%	8.09%
309	Exported protein 2 OS=Plasmodium falciparum (isolate 3D7) GN=EXP-2 PE=4 SV=1	Q8IKC8_PLAF7	33 kDa	1.2	2	3	2	3	2	1	9.06%	12.20%	9.06%	6.97%	3.83%	3.83%
310	DNA/RNA-binding protein Alba, putative OS=Plasmodium falciparum (isolate 3D7) GN=PF08_0074 PE=4 SV=1	Q8IAX8_PLAF7	27 kDa	1.2	5	5	5	7	4	2	16.90%	12.10%	12.50%	17.30%	12.90%	8.06%
311	Cluster of Alpha-adducin OS=Homo sapiens GN=ADD1 PE=1 SV=2 (ADDA_HUMAN)	ADDA_HUMAN [3]	81 kDa	1.1	3	2	3	3	3	0	4.34%	3.75%	8.25%	10.80%	10.20%	1
311.1	Alpha-adducin OS=Homo sapiens GN=ADD1 PE=1 SV=2	ADDA_HUMAN (+2)	81 kDa	1.1	3	2	3	3	3	0	4.34%	2.04%	4.48%	5.83%	5.56%	1
311.2	Alpha-adducin OS=Homo sapiens GN=ADD1 PE=1 SV=1	A0A0A0MSR2_HUMAN	44 kDa	1	0	2	3	3	3	0	1	3.75%	8.25%	10.80%	10.20%	1
312	40S ribosomal protein S3, putative OS=Plasmodium falciparum (isolate 3D7) GN=PF14_0627 PE=1 SV=1	Q8IKH8_PLAF7	25 kDa	1.1	2	4	3	4	3	1	11.80%	21.30%	15.40%	21.30%	10.40%	5.88%
313	Uncharacterized protein OS=Plasmodium falciparum (isolate 3D7) GN=PF10_0047 PE=4 SV=1	Q8IJZ3_PLAF7	106 kDa	1	1	2	0	2	1	0	1.14%	2.27%	0.00%	2.39%	1.25%	0.00%
314	Formate-nitrate transporter, putative OS=Plasmodium falciparum (isolate 3D7) GN=PFC0725c PE=4 SV=2	O77389_PLAF7	34 kDa	1	1	1	2	2	0	0	3.88%	3.56%	3.88%	6.15%	0.00%	1
315	60S ribosomal protein L44 OS=Plasmodium falciparum (isolate 3D7) GN=RPL44 PE=1 SV=3	RL44_PLAF7	12 kDa	1	1	1	2	2	1	1	10.60%	10.60%	10.60%	18.30%	10.60%	10.60%
316	Hypoxanthine phosphoribosyltransferase OS=Plasmodium falciparum (isolate 3D7) GN=PF10_0121 PE=4 SV=1	Q8IJS1_PLAF7	26 kDa	1	2	3	0	4	0	0	7.36%	15.60%	1	24.20%	1	1
317	Uncharacterized protein OS=Plasmodium falciparum (isolate 3D7) GN=PFL1270w PE=4 SV=1	Q8I5F4_PLAF7	33 kDa	1	2	1	2	2	2	0	8.81%	3.05%	7.80%	6.78%	4.41%	1
318	Protein phosphatase, putative OS=Plasmodium falciparum (isolate 3D7) GN=PF11_0281 PE=4 SV=2	Q8II93_PLAF7	34 kDa	1	0	2	1	2	1	0	0.00%	10.50%	4.88%	3.14%	4.88%	1
319	Uncharacterized protein OS=Plasmodium falciparum (isolate 3D7) GN=PFE0050w PE=4 SV=1	Q8I490_PLAF7	31 kDa	1	3	1	1	3	0	0	9.62%	5.77%	3.85%	9.62%	1	1
320	Peptidyl-prolyl cis-trans isomerase OS=Plasmodium falciparum (isolate 3D7) GN=PfCyP19 PE=3 SV=1	Q76NN7_PLAF7	19 kDa	1	1	0	2	2	1	1	5.26%	1	11.70%	11.10%	5.85%	5.85%
321	Proteasome regulatory component, putative OS=Plasmodium falciparum (isolate 3D7) GN=MAL13P1.190 PE=4 SV=1	Q8IDV2_PLAF7	59 kDa	1	0	2	0	1	0	2	0.00%	4.77%	0.00%	2.19%	1	5.17%
322	Metabolite/drug transporter, putative OS=Plasmodium falciparum (isolate 3D7) GN=PFE0785c PE=4 SV=1	Q8I3V1_PLAF7	52 kDa	1	1	2	1	2	1	0	1.75%	4.17%	1.75%	4.17%	1.75%	1
323	Phosphatidylserine synthase I, putative OS=Plasmodium falciparum (isolate 3D7) GN=MAL13P1.335 PE=4 SV=1	C0H5K9_PLAF7	42 kDa	1	2	1	1	0	2	1	10.90%	2.80%	2.80%	0.00%	2.52%	2.52%
324	Thioredoxin OS=Plasmodium falciparum (isolate 3D7) GN=PF14_0545 PE=1 SV=1	THIO_PLAF7	12 kDa	1	1	2	1	1	1	2	9.62%	21.20%	11.50%	11.50%	11.50%	11.50%
325	Cluster of ADP-ribosylation factor 5 OS=Homo sapiens GN=ARF5 PE=1 SV=2 (ARF5_HUMAN)	ARF5_HUMAN	21 kDa	1	2	0	0	2	1	0	18.60%	1	1	18.60%	6.67%	1
325.1	ADP-ribosylation factor 5 OS=Homo sapiens GN=ARF5 PE=1 SV=2	ARF5_HUMAN (+1)	21 kDa	1	1	0	0	2	1	0	6.11%	1	1	6.11%	6.67%	1
325.2	ADP-ribosylation factor 1 OS=Plasmodium falciparum (isolate 3D7) GN=ARF1 PE=1 SV=1	ARF1_PLAF7	21 kDa	1	2	0	0	0	0	0	13.80%	1	1	1	1	1
325.3	Uncharacterized protein OS=Homo sapiens PE=3 SV=1	F5H423_HUMAN	23 kDa	1	2	0	0	0	0	0	11.90%	1	1	1	1	1
325.4	ADP-ribosylation factor 4 OS=Homo sapiens GN=ARF4 PE=1 SV=1	F8WDB3_HUMAN	7 kDa	1	0	0	0	2	0	0	1	1	1	18.60%	1	1
326	Superoxide dismutase [Fe] OS=Plasmodium falciparum (isolate 3D7) GN=SODB PE=1 SV=1	SODF_PLAF7	23 kDa	1	1	2	1	1	1	2	7.07%	13.10%	7.07%	6.06%	6.06%	6.06%
327	Uncharacterized protein OS=Plasmodium falciparum (isolate 3D7) GN=PFI0605c PE=4 SV=1	Q8I328_PLAF7	53 kDa	1	2	0	0	2	0	0	4.71%	1	1	4.71%	1	1
328	Histone H4 OS=Plasmodium falciparum (isolate 3D7) GN=PF11_0061 PE=3 SV=1	Q8IIV2_PLAF7	11 kDa	0.9	5	4	5	7	5	4	31.10%	31.10%	19.40%	42.70%	19.40%	21.40%
329	Uncharacterized protein OS=Plasmodium falciparum (isolate 3D7) GN=PF11_0302 PE=4 SV=1	Q8II72_PLAF7	52 kDa	0.9	1	2	3	4	2	0	2.43%	5.31%	7.08%	7.08%	2.43%	1
330	40S ribosomal protein S25, putative OS=Plasmodium falciparum (isolate 3D7) GN=PF14_0205 PE=1 SV=2	Q8ILN8_PLAF7	12 kDa	0.9	1	3	2	2	3	2	13.30%	21.90%	21.90%	17.10%	20.00%	8.57%
331	Hsp60 OS=Plasmodium falciparum (isolate 3D7) GN=PF10_0153 PE=3 SV=1	Q8IJN9_PLAF7	63 kDa	0.8	1	2	2	4	1	0	2.07%	4.14%	4.14%	8.45%	2.24%	1
332	Cluster of Dematin OS=Homo sapiens GN=DMTN PE=1 SV=3 (DEMA_HUMAN)	DEMA_HUMAN	46 kDa	0.8	3	3	2	6	2	2	15.60%	11.90%	6.67%	22.90%	10.60%	6.42%
332.1	Dematin (Fragment) OS=Homo sapiens GN=DMTN PE=1 SV=2	E5RFK4_HUMAN (+1)	25 kDa	1	0	0	0	0	0	0	1	1	1	1	1	1
332.2	Dematin OS=Homo sapiens GN=DMTN PE=1 SV=3	DEMA_HUMAN	46 kDa	0.8	3	3	2	6	2	2	11.60%	9.63%	6.67%	15.10%	5.68%	3.46%
333	Uncharacterized protein OS=Plasmodium falciparum (isolate 3D7) GN=MAL8P1.206 PE=4 SV=1	C0H4Z7_PLAF7	36 kDa	0.8	1	2	0	2	2	0	3.64%	3.64%	0.00%	6.62%	6.62%	1
334	Blood group Rh(CE) polypeptide OS=Homo sapiens GN=RHCE PE=4 SV=1	E7EQ47_HUMAN (+3)	29 kDa	0.8	2	1	1	3	1	0	3.38%	3.38%	3.38%	6.39%	3.38%	1
335	Putative chloroquine resistance transporter OS=Plasmodium falciparum (isolate 3D7) GN=CG10 PE=3 SV=1	CRT_PLAF7	49 kDa	0.8	0	2	1	2	2	0	1	6.37%	3.07%	5.19%	4.95%	1
336	Triosephosphate isomerase OS=Plasmodium falciparum (isolate 3D7) GN=TPI PE=3 SV=1	TPIS_PLAF7	28 kDa	0.8	4	3	4	8	5	1	14.90%	14.50%	10.90%	14.90%	9.68%	6.05%
337	Peptidase, putative OS=Plasmodium falciparum (isolate 3D7) GN=MAL13P1.19 PE=4 SV=1	Q8IES7_PLAF7-DECOY	?	0.8	0	0	0	1	2	0	0.00%	0.00%	0.00%	0.00%	0.00%	1
338	Uncharacterized protein OS=Plasmodium falciparum (isolate 3D7) GN=MAL4P1.163 PE=4 SV=1	C0H4B0_PLAF7-DECOY	?	0.8	0	0	0	0	2	0	0.00%	0.00%	0.00%	0.00%	0.00%	0.00%
339	Uncharacterized protein OS=Plasmodium falciparum (isolate 3D7) GN=MAL13P1.380 PE=4 SV=1	C0H5G6_PLAF7	906 kDa	0.8	0	0	0	1	2	0	0.00%	0.00%	0.00%	0.15%	0.53%	1
340	Uncharacterized protein OS=Plasmodium falciparum (isolate 3D7) GN=PFF0445w PE=4 SV=1	C6KST7_PLAF7	721 kDa	0.8	1	1	1	1	2	0	0.18%	0.18%	0.18%	0.18%	0.46%	0.00%
341	Uncharacterized protein OS=Plasmodium falciparum (isolate 3D7) GN=PF14_0419 PE=4 SV=2	Q8IL30_PLAF7	865 kDa	0.8	1	0	0	1	2	0	0.42%	0.00%	0.00%	0.18%	0.48%	0.00%
342	Conserved Plasmodium membrane protein OS=Plasmodium falciparum (isolate 3D7) GN=PF10_0211 PE=4 SV=2	Q8IJI6_PLAF7-DECOY	?	0.8	0	0	0	0	2	0	0.00%	0.00%	0.00%	0.00%	0.00%	0.00%
343	Erythrocyte membrane protein 1, PfEMP1 OS=Plasmodium falciparum (isolate 3D7) GN=VAR PE=4 SV=1	Q8IEU9_PLAF7	386 kDa	0.8	1	0	0	0	2	0	0.42%	0.00%	0.00%	0.00%	0.81%	0.00%
344	Protein kinase, putative OS=Plasmodium falciparum (isolate 3D7) GN=PFC0485w PE=4 SV=3	O77365_PLAF7	296 kDa	0.8	0	0	0	0	0	2	0.00%	0.00%	0.00%	0.00%	0.00%	1.31%
345	Uncharacterized protein OS=Plasmodium falciparum (isolate 3D7) GN=PFE0320w PE=4 SV=1	C0H4C9_PLAF7-DECOY	?	0.8	0	0	0	0	2	0	0.00%	0.00%	0.00%	0.00%	0.00%	0.00%
346	Dynein heavy chain-like protein PF11_0240 OS=Plasmodium falciparum (isolate 3D7) GN=PF11_0240 PE=3 SV=1	DYHC2_PLAF7-DECOY	?	0.8	0	0	0	0	2	0	0.00%	0.00%	0.00%	0.00%	0.00%	0.00%
347	Uncharacterized protein OS=Plasmodium falciparum (isolate 3D7) GN=PF13_0148 PE=4 SV=1	C0H5D9_PLAF7	575 kDa	0.8	0	0	0	0	2	1	0.00%	0.00%	0.00%	0.00%	0.92%	0.63%
348	Uncharacterized protein PF07_0086 OS=Plasmodium falciparum (isolate 3D7) GN=PF07_0086 PE=3 SV=1	YPF16_PLAF7	417 kDa	0.8	0	0	0	0	2	0	0.00%	0.00%	0.00%	0.00%	1.14%	0.00%
349	Cluster of Protein piccolo OS=Homo sapiens GN=PCLO PE=1 SV=4 (PCLO_HUMAN-DECOY)	PCLO_HUMAN-DECOY	?	0.8	0	1	0	2	0	0	0.00%	0.00%	0.00%	0.00%	0.00%	0.00%
349.1	Protein piccolo OS=Homo sapiens GN=PCLO PE=1 SV=1	E9PE96_HUMAN-DECOY	?	1	0	0	0	0	0	0	1	1	1	1	1	1
349.2	Protein piccolo OS=Homo sapiens GN=PCLO PE=1 SV=4	PCLO_HUMAN-DECOY	?	0.8	0	1	0	2	0	0	0.00%	0.00%	1	0.00%	0.00%	0.00%
350	Erythrocyte membrane protein 1, PfEMP1 OS=Plasmodium falciparum (isolate 3D7) GN=VAR PE=4 SV=1	Q8IAK2_PLAF7-DECOY	?	0.8	0	0	1	0	2	0	0.00%	0.00%	0.00%	0.00%	0.00%	1
351	Erythrocyte membrane protein 1, PfEMP1 OS=Plasmodium falciparum (isolate 3D7) GN=VAR PE=4 SV=1	Q8I218_PLAF7	398 kDa	0.8	0	0	0	0	2	0	0.00%	0.00%	0.00%	0.00%	0.84%	1
352	Low-density lipoprotein receptor-related protein 1B OS=Homo sapiens GN=LRP1B PE=1 SV=1	A0A087WUR9_HUMAN (	495 kDa	0.8	0	0	0	0	2	0	0.00%	0.00%	0.00%	0.00%	1.07%	1
353	Conserved Plasmodium protein OS=Plasmodium falciparum (isolate 3D7) GN=PF10_0190 PE=4 SV=2	Q8IJK5_PLAF7-DECOY	?	0.8	0	0	0	0	2	0	0.00%	0.00%	0.00%	0.00%	0.00%	0.00%
354	E3 ubiquitin-protein ligase MYCBP2 OS=Homo sapiens GN=MYCBP2 PE=1 SV=3	MYCB2_HUMAN	510 kDa	0.8	0	0	0	0	2	0	0.00%	0.00%	0.00%	0.00%	1.08%	0.00%
355	Nipped-B-like protein OS=Homo sapiens GN=NIPBL PE=1 SV=2	NIPBL_HUMAN-DECOY	?	0.8	1	0	0	0	2	0	0.00%	1	1	0.00%	0.00%	1
356	Uncharacterized protein OS=Plasmodium falciparum (isolate 3D7) GN=MAL13P1.266 PE=4 SV=1	C0H5I2_PLAF7	190 kDa	0.8	0	0	0	2	0	0	0.00%	1	0.00%	2.01%	0.00%	0.00%
357	Uncharacterized protein OS=Plasmodium falciparum (isolate 3D7) GN=MAL13P1.124 PE=4 SV=1	C0H5D0_PLAF7-DECOY	?	0.8	0	0	0	0	2	0	1	0.00%	0.00%	0.00%	0.00%	1
358	Conserved Plasmodium protein OS=Plasmodium falciparum (isolate 3D7) GN=PFB0190c PE=4 SV=1	Q9TY98_PLAF7-DECOY	?	0.8	0	0	0	0	2	0	1	0.00%	0.00%	0.00%	0.00%	1
359	Uncharacterized protein OS=Plasmodium falciparum (isolate 3D7) GN=MAL13P1.151 PE=4 SV=1	Q8IE41_PLAF7	261 kDa	0.8	0	0	0	0	2	0	0.00%	0.00%	0.00%	0.00%	2.53%	0.00%
360	Dynein heavy chain, putative OS=Plasmodium falciparum (isolate 3D7) GN=PFL0115w PE=4 SV=1	Q8I622_PLAF7	692 kDa	0.8	0	0	0	0	2	0	1	0.00%	0.00%	0.00%	0.40%	1
361	Prolow-density lipoprotein receptor-related protein 1 OS=Homo sapiens GN=LRP1 PE=1 SV=2	LRP1_HUMAN	505 kDa	0.8	0	0	0	0	2	0	0.00%	0.00%	0.00%	1	1.34%	0.00%
362	Erythrocyte membrane protein 1, PfEMP1 OS=Plasmodium falciparum (isolate 3D7) GN=VAR PE=4 SV=1	Q8ID12_PLAF7-DECOY	?	0.8	0	0	0	0	2	0	0.00%	0.00%	0.00%	0.00%	0.00%	0.00%
363	Protein kinase, putative OS=Plasmodium falciparum (isolate 3D7) GN=PfTKL-5 PE=4 SV=1	C6KT74_PLAF7-DECOY	?	0.8	0	0	0	0	2	0	0.00%	1	0.00%	0.00%	0.00%	0.00%
364	Lipid/sterol:H+ symporter OS=Plasmodium falciparum (isolate 3D7) GN=PFA_0375c PE=4 SV=1	Q8I266_PLAF7	170 kDa	0.8	0	0	1	2	0	0	0.00%	1	0.68%	1.63%	0.00%	1

365	Uncharacterized protein OS=Plasmodium falciparum (isolate 3D7) GN=PFE1485w PE=4 SV=1	Q8I3H0_PLAF7	226 kDa	0.8	0	0	0	0	2	0	0.00%	0.00%	0.00%	0.00%	1.05%	1
366	Uncharacterized protein MAL13P1.304 OS=Plasmodium falciparum (isolate 3D7) GN=MAL13P1.304 PE=4 SV=1	YPF12_PLAF7	210 kDa	0.8	0	0	0	0	2	0	0.00%	0.00%	1	1	1.45%	1
367	Piwi-like protein 1 OS=Homo sapiens GN=PIWIL1 PE=1 SV=1	PIWL1_HUMAN-DECOY	?	0.8	0	0	0	1	2	0	1	1	1	0.00%	0.00%	1
368	Uncharacterized protein MAL13P1.304 OS=Plasmodium falciparum (isolate 3D7) GN=MAL13P1.304 PE=4 SV=1	YPF12_PLAF7-DECOY	?	0.8	0	0	0	0	2	0	0.00%	0.00%	1	0.00%	0.00%	1
369	PR domain zinc finger protein 15 OS=Homo sapiens GN=PRDM15 PE=1 SV=4	PRD15_HUMAN	169 kDa	0.8	0	0	0	0	2	0	1	1	0.00%	1	2.19%	0.00%
370	Glideosome-associated protein 50 OS=Plasmodium falciparum (isolate 3D7) GN=GAP50 PE=1 SV=1	Q8I2X3_PLAF7	45 kDa	0.8	1	1	1	2	1	1	2.78%	2.78%	2.78%	5.81%	2.78%	3.03%
371	DNA repair endonuclease, putative OS=Plasmodium falciparum (isolate 3D7) GN=MAL13P1.346 PE=4 SV=1	Q8ID22_PLAF7-DECOY	?	0.8	0	0	0	0	2	0	0.00%	1	1	0.00%	0.00%	1
372	Cluster of Fibrillarin, putative OS=Plasmodium falciparum (isolate 3D7) GN=PF14_0068 PE=3 SV=1 (Q8IM23_PLAF7)	Q8IM23_PLAF7	34 kDa	0.8	0	0	0	0	2	0	0.00%	0.00%	0.00%	1	15.20%	1
372.1	Fibrillarin, putative OS=Plasmodium falciparum (isolate 3D7) GN=PF14_0068 PE=3 SV=1	Q8IM23_PLAF7	34 kDa	1	0	0	0	0	1	0	0.00%	1	0.00%	1	3.77%	1
372.2	rRNA 2'-O-methyltransferase fibrillarin (Fragment) OS=Homo sapiens GN=FBL PE=1 SV=1	M0R1H0_HUMAN	18 kDa	1	0	0	0	0	2	0	1	1	1	1	15.20%	1
372.3	rRNA 2'-O-methyltransferase fibrillarin (Fragment) OS=Homo sapiens GN=FBL PE=1 SV=1	M0R2Q4_HUMAN	29 kDa	1	0	0	0	0	2	0	1	1	1	1	9.89%	1
373	Phosphatase, putative OS=Plasmodium falciparum (isolate 3D7) GN=PF14_0614 PE=4 SV=2	Q8IKJ1_PLAF7	170 kDa	0.8	0	0	0	2	0	0	1	0.00%	1	1.53%	0.00%	0.00%
374	Coiled-coil domain-containing protein 158 OS=Homo sapiens GN=CCDC158 PE=1 SV=2	CD158_HUMAN	127 kDa	0.8	0	0	0	0	2	0	1	1	0.00%	1	2.79%	1
375	Uncharacterized protein OS=Plasmodium falciparum (isolate 3D7) GN=PF14_0652 PE=4 SV=1	Q8IKF3_PLAF7-DECOY	?	0.8	0	0	0	0	2	0	0.00%	1	1	0.00%	0.00%	1
376	Protein ELYS OS=Homo sapiens GN=AHCTF1 PE=1 SV=3	ELYS_HUMAN-DECOY	?	0.8	0	0	0	1	2	0	1	1	1	0.00%	0.00%	1
377	Uncharacterized protein OS=Plasmodium falciparum (isolate 3D7) GN=PF14_0703 PE=4 SV=1	Q8IKA3_PLAF7	104 kDa	0.8	0	0	0	0	2	0	1	0.00%	0.00%	1	3.41%	1
378	Uncharacterized protein OS=Plasmodium falciparum (isolate 3D7) GN=PF11_0384 PE=4 SV=1	Q8IHZ2_PLAF7	82 kDa	0.8	1	1	1	2	0	0	1.60%	1.60%	1.60%	2.76%	1	1
379	Adenomatous polyposis coli protein 2 OS=Homo sapiens GN=APC2 PE=1 SV=1	APC2_HUMAN	244 kDa	0.8	0	0	0	2	1	0	1	1	1	1.48%	0.56%	1
380	Collagen alpha-5(VI) chain OS=Homo sapiens GN=COL6A5 PE=1 SV=1	CO6A5_HUMAN	290 kDa	0.8	0	0	0	0	2	0	1	1	1	1	1.15%	1
381	Protein FAM83H OS=Homo sapiens GN=FAM83H PE=1 SV=3	FA83H_HUMAN	127 kDa	0.8	0	0	0	0	2	0	1	1	1	1	2.46%	0.00%
382	Pappalysin-1 OS=Homo sapiens GN=PAPPA PE=1 SV=3	PAPP1_HUMAN-DECOY	?	0.8	0	0	0	0	2	1	0.00%	1	1	0.00%	0.00%	0.00%
383	40S ribosomal protein S14, putative OS=Plasmodium falciparum (isolate 3D7) GN=PFE0810c PE=1 SV=1	Q8I3U6_PLAF7	16 kDa	0.8	1	1	0	1	2	0	9.93%	9.93%	1	9.93%	18.50%	1
384	Ribosomal protein S20e, putative OS=Plasmodium falciparum (isolate 3D7) GN=PF10_0038 PE=1 SV=1	Q8IK02_PLAF7	14 kDa	0.8	1	1	1	1	2	0	7.63%	7.63%	7.63%	7.63%	17.80%	1
385	Metallothionein-1L OS=Homo sapiens GN=MT1L PE=2 SV=1	MT1L_HUMAN-DECOY	?	0.8	0	0	1	0	2	0	1	1	0.00%	1	0.00%	1
386	Tubulin--tyrosine ligase-like protein 12 OS=Homo sapiens GN=TTLL12 PE=1 SV=2	TTL12_HUMAN	74 kDa	0.8	0	0	0	2	1	0	1	0.00%	0.00%	3.73%	1.86%	1
387	A-kinase anchor protein 12 OS=Homo sapiens GN=AKAP12 PE=1 SV=4	AKA12_HUMAN	191 kDa	0.8	0	0	0	0	2	0	1	1	1	0.00%	2.75%	1
388	Structure specific recognition protein OS=Plasmodium falciparum (isolate 3D7) GN=PF14_0393 PE=4 SV=1	Q8IL56_PLAF7	59 kDa	0.8	0	0	0	2	0	0	1	1	0.00%	4.55%	0.00%	1
389	Aquaglyceroporin OS=Plasmodium falciparum (isolate 3D7) GN=PF11_0338 PE=3 SV=1	Q8II36_PLAF7	28 kDa	0.8	1	0	1	2	0	0	3.88%	1	3.88%	6.98%	1	1
390	Hamartin OS=Homo sapiens GN=TSC1 PE=1 SV=2	TSC1_HUMAN	130 kDa	0.8	0	0	0	0	2	0	1	1	1	1	3.09%	1
391	Uncharacterized protein OS=Plasmodium falciparum (isolate 3D7) GN=PFL1870c PE=4 SV=1	Q8I537_PLAF7	46 kDa	0.8	1	0	1	2	0	0	3.56%	1	3.56%	6.87%	1	1
392	1-deoxy-D-xylulose 5-phosphate synthase OS=Plasmodium falciparum (isolate 3D7) GN=MAL13P1.186 PE=4 SV=1	Q8IDW0_PLAF7	140 kDa	0.8	0	0	0	0	2	0	1	1	1	1	1.99%	1
393	ARHGAP12 protein OS=Homo sapiens GN=ARHGAP12 PE=1 SV=1	Q1RLN5_HUMAN (+1)	91 kDa	0.8	0	0	0	0	2	0	1	0.00%	1	1	3.63%	1
394	Membrane associated histidine-rich protein, MAHRP-1 OS=Plasmodium falciparum (isolate 3D7) GN=MAHRP1 PE=4 SV=[illegible]	C0H5L9_PLAF7	29 kDa	0.8	0	1	1	2	0	0	1	4.02%	4.02%	10.80%	1	1
395	Keratin, type II cytoskeletal 80 OS=Homo sapiens GN=KRT80 PE=1 SV=2	K2C80_HUMAN	51 kDa	0.8	0	0	0	2	1	0	1	1	1	4.42%	2.21%	0.00%
396	Kinesin-associated protein 3 OS=Homo sapiens GN=KIFAP3 PE=1 SV=2	KIFA3_HUMAN-DECOY	?	0.8	0	0	0	2	0	0	1	1	1	0.00%	1	1
397	Contactin-4 OS=Homo sapiens GN=CNTN4 PE=1 SV=1	CNTN4_HUMAN-DECOY	?	0.8	0	0	0	2	0	0	1	1	1	0.00%	1	1
398	Uncharacterized protein OS=Plasmodium falciparum (isolate 3D7) GN=PF14_0105 PE=4 SV=1	Q8ILY8_PLAF7	40 kDa	0.8	1	1	0	2	0	0	2.99%	2.99%	1	5.99%	1	1
399	phospholipase A2 inhibitor and Ly6/PLAUR domain-containing protein OS=Homo sapiens GN=PINLYP PE=2 SV=3	PINLY_HUMAN-DECOY	?	0.8	0	0	0	0	2	0	1	1	1	1	0.00%	1
400	WAP four-disulfide core domain protein 8 OS=Homo sapiens GN=WFDC8 PE=2 SV=2	WFDC8_HUMAN	28 kDa	0.8	0	0	0	0	2	0	1	1	1	0.00%	13.70%	1
401	Uncharacterized protein OS=Plasmodium falciparum (isolate 3D7) GN=PF10_0063 PE=4 SV=1	Q8IJX8_PLAF7	12 kDa	0.8	0	0	0	2	0	0	1	1	1	19.60%	1	1
402	D(1A) dopamine receptor OS=Homo sapiens GN=DRD1 PE=1 SV=1	DRD1_HUMAN-DECOY	?	0.8	0	0	0	0	2	0	1	1	1	1	0.00%	1
403	Conserved Plasmodium protein OS=Plasmodium falciparum (isolate 3D7) GN=PF10_0104 PE=4 SV=1	Q8IJT8_PLAF7	26 kDa	0.8	0	0	1	2	0	0	1	1	6.73%	12.10%	1	1
404	Flotillin-2 OS=Homo sapiens GN=FLOT2 PE=4 SV=1	J3QKZ4_HUMAN-DECOY	?	0.8	0	0	0	0	2	0	1	1	1	1	0.00%	1
405	Keratin, type I cytoskeletal 26 OS=Homo sapiens GN=KRT26 PE=1 SV=2	K1C26_HUMAN	52 kDa	0.8	0	0	0	2	0	0	1	1	1	4.49%	1	1
406	Flavin reductase (NADPH) OS=Homo sapiens GN=BLVRB PE=1 SV=3	BLVRB_HUMAN	22 kDa	0.7	3	0	1	5	1	0	11.70%	1	6.80%	19.90%	6.80%	1
407	Nucleosome assembly protein OS=Plasmodium falciparum (isolate 3D7) GN=PFI0930c PE=3 SV=1	Q8I2W3_PLAF7	32 kDa	0.7	2	1	1	2	3	0	7.06%	3.72%	3.35%	7.43%	10.80%	1
408	Hydrolase, putative OS=Plasmodium falciparum (isolate 3D7) GN=PF13_0032 PE=4 SV=1	Q8IEQ3_PLAF7	57 kDa	0.7	1	2	1	4	1	0	2.93%	5.02%	2.09%	7.32%	2.09%	1
409	Ubiquitin carboxyl-terminal hydrolase, putative OS=Plasmodium falciparum (isolate 3D7) GN=MAL7P1.147 PE=4 SV=1	Q8IBJ1_PLAF7-DECOY	?	0.6	0	0	0	0	3	0	0.00%	0.00%	0.00%	0.00%	0.00%	1
410	Transcription factor TFIIIB component B'' homolog OS=Homo sapiens GN=BDP1 PE=1 SV=1	A0A0G2JNU3_HUMAN-D	?	0.6	0	0	0	3	0	0	0.00%	1	0.00%	0.00%	0.00%	0.00%
411	AF4/FMR2 family member 1 OS=Homo sapiens GN=AFF1 PE=1 SV=1	AFF1_HUMAN	131 kDa	0.6	0	0	0	0	3	0	1	1	1	0.00%	2.73%	1
412	REX2 protein OS=Plasmodium falciparum (isolate 3D7) GN=REX2 PE=4 SV=1	C0H592_PLAF7	11 kDa	0.6	1	1	0	2	2	0	13.80%	13.80%	1	25.50%	13.80%	1
413	Cluster of Keratin-associated protein 4-8 OS=Homo sapiens GN=KRTAP4-8 PE=2 SV=4 (KRA48_HUMAN-DECOY)	KRA48_HUMAN-DECOY [	?	0.6	0	0	0	0	3	0	1	1	1	1	0.00%	0.00%
413.1	Keratin-associated protein 4-8 OS=Homo sapiens GN=KRTAP4-8 PE=2 SV=4	KRA48_HUMAN-DECOY	?	0.8	0	0	0	0	2	0	1	1	1	1	0.00%	0.00%
413.2	Keratin-associated protein 4-4 OS=Homo sapiens GN=KRTAP4-4 PE=2 SV=1	KRA44_HUMAN-DECOY	?	0.8	0	0	0	0	2	0	1	1	1	1	0.00%	1
414	Heat shock protein hsp70 homologue OS=Plasmodium falciparum (isolate 3D7) GN=PF11_0351 PE=3 SV=1	Q8II24_PLAF7	73 kDa	0.6	2	1	0	3	1	3	3.02%	1.51%	1	5.13%	1.81%	3.92%
415	Cluster of Ras and Rab interactor 3 OS=Homo sapiens GN=RIN3 PE=1 SV=1 (A0A087WWY9_HUMAN-DECOY)	A0A087WWY9_HUMAN-	?	0.6	1	0	2	2	3	2	0.00%	1	0.00%	0.00%	0.00%	0.00%
415.1	Ras and Rab interactor 3 OS=Homo sapiens GN=RIN3 PE=1 SV=1	G3V2I7_HUMAN-DECOY	?	1	1	0	0	0	0	1	0.00%	1	1	1	1	0.00%
415.2	Ras and Rab interactor 3 OS=Homo sapiens GN=RIN3 PE=1 SV=1	A0A087WWY9_HUMAN-	?	0.6	1	0	2	2	3	2	0.00%	1	0.00%	0.00%	0.00%	0.00%
416	Cluster of Nebulin OS=Homo sapiens GN=NEB PE=1 SV=1 (A0A087X1N7_HUMAN)	A0A087X1N7_HUMAN	991 kDa	0.5	0	0	0	0	4	0	0.00%	0.00%	0.00%	0.00%	0.62%	0.00%
416.1	Nebulin OS=Homo sapiens GN=NEB PE=1 SV=5	NEBU_HUMAN	773 kDa	1	0	0	0	0	3	0	0.00%	1	1	0.00%	0.60%	0.00%
416.2	Nebulin OS=Homo sapiens GN=NEB PE=1 SV=1	A0A087X1N7_HUMAN	991 kDa	0.5	0	0	0	0	4	0	0.00%	0.00%	0.00%	0.00%	0.62%	0.00%
417	Cysteine repeat modular protein 4, putative OS=Plasmodium falciparum (isolate 3D7) GN=PCRMP 4 PE=4 SV=2	Q8IK84_PLAF7-DECOY	?	0.5	0	0	0	0	4	0	0.00%	0.00%	0.00%	0.00%	0.00%	0.00%
418	Cluster of Peroxiredoxin-2 OS=Homo sapiens GN=PRDX2 PE=1 SV=5 (PRDX2_HUMAN)	PRDX2_HUMAN	22 kDa	0.5	1	1	1	3	2	0	5.05%	4.04%	4.04%	14.60%	9.60%	1
418.1	Peroxiredoxin-1 OS=Homo sapiens GN=PRDX1 PE=1 SV=1	A0A0A0MRQ5_HUMAN (	11 kDa	1	0	0	0	0	0	0	1	1	1	1	1	1
418.2	Peroxiredoxin-2 OS=Homo sapiens GN=PRDX2 PE=1 SV=5	PRDX2_HUMAN	22 kDa	0.5	1	1	1	3	2	0	5.05%	4.04%	4.04%	14.60%	9.60%	1
419	Erythrocyte membrane protein 1, PfEMP1 OS=Plasmodium falciparum (isolate 3D7) GN=VAR PE=4 SV=1	Q8I5L5_PLAF7-DECOY	?	0.5	0	0	0	0	4	0	1	1	0.00%	0.00%	0.00%	1
420	Tropomodulin-1 OS=Homo sapiens GN=TMOD1 PE=1 SV=1	TMOD1_HUMAN	41 kDa	0.5	1	2	0	6	0	0	2.79%	2.79%	1	13.60%	1	1
421	Biliverdin reductase A OS=Homo sapiens GN=BLVRA PE=1 SV=2	BIEA_HUMAN	33 kDa	0.5	0	0	0	4	1	1	1	1	1	12.50%	4.73%	3.04%
422	Uncharacterized protein OS=Plasmodium falciparum (isolate 3D7) GN=PF11_0179 PE=4 SV=1	Q8IIJ4_PLAF7	15 kDa	0.4	1	1	1	5	1	0	7.81%	7.81%	7.81%	32.00%	7.03%	1
423	Carbonic anhydrase 2 OS=Homo sapiens GN=CA2 PE=1 SV=2	CAH2_HUMAN	29 kDa	0.4	1	3	0	7	6	1	3.46%	14.60%	1	15.40%	15.40%	4.62%
424	Cluster of Histone H2A OS=Plasmodium falciparum (isolate 3D7) GN=PFC0920w PE=3 SV=1 (O97320_PLAF7)	O97320_PLAF7	16 kDa	0.3	2	0	1	7	4	0	12.00%	1	7.03%	26.60%	19.00%	1
424.1	Histone H2A OS=Plasmodium falciparum (isolate 3D7) GN=PFF0860c PE=3 SV=1	C6KT18_PLAF7	14 kDa	1	1	0	1	2	2	0	6.82%	1	6.82%	6.82%	6.82%	1
424.2	Histone H2A.J OS=Homo sapiens GN=H2AFJ PE=1 SV=1	H2AJ_HUMAN	14 kDa	1	1	0	0	2	2	0	6.98%	1	1	6.98%	6.98%	1
424.3	Histone H2A.Z OS=Homo sapiens GN=H2AFZ PE=1 SV=2	H2AZ_HUMAN	14 kDa	1	0	0	1	5	0	0	1	1	7.03%	18.00%	1	1
424.4	Histone H2AX OS=Homo sapiens GN=H2AFX PE=1 SV=2	H2AX_HUMAN	15 kDa	1	0	0	0	0	0	0	1	1	1	1	1	1
424.5	Histone H2A OS=Plasmodium falciparum (isolate 3D7) GN=PFC0920w PE=3 SV=1	O97320_PLAF7	16 kDa	0.3	2	0	1	7	4	0	12.00%	1	5.70%	26.60%	19.00%	1
425	Flotillin-2 OS=Homo sapiens GN=FLOT2 PE=1 SV=1	E7EMK3_HUMAN	53 kDa	0.3	1	1	0	7	0	0	2.69%	2.28%	1	10.40%	0.00%	0.00%

Figure S1. FASTA sequences of DEAD/DEAH box Helicase and Methionyl-tRNA formyltransferase in isolate 3D7 and other proteome databases from UniProt.

A. FASTA sequence of Methionyl-tRNA formyltransferase from isolate 3D7 proteome database. The Cys is at the 5th position from the C-terminus.

```
>tr|Q8IEI1|Q8IEI1_PLAF7 Methionyl-tRNA formyltransferase, putative
OS=Plasmodium falciparum (isolate 3D7) GN=PF3D7_1313200 PE=4 SV=2
MYVKCFFYVIQILFIIFLKCHCYKIKCFNILDLNKKKYYSFREHINCEHIRNSVNRNNLS
NVLLRRRTKNALVKELYVSKLKDNYKTHTNFIRTNNIFLEEDKKIQECNINNIINNNVDI
QENVEKYNILYKNQLDDINILYILLFNTLMIYKKKYDFFMNENYIRSYYYIYKNNLGRDK
KIYNTKNYFINTFSITWYNTIKPYMNNIFLEILNIIENTLGNKIFYIDIKQELNKNRDEI
INTLYNIYKGNFRKRDKKPIKLLFIGSNEYSNLCFKIILLIIKRLRNDIILDNVITKSPR
RKGRNLILKKSNVEDEAIKNNINVFYYDKLKNNIHMLQNKKMDLCISISFGEIFNCNFFK
TIKSNIFSLHPSLLPFYKGASPIQRSLLNNEILYGYSVFLTTLNIDSGNVIMKKPFWFNS
NYNFNDIITILFTQGTLSLLKNISYLANYNKDIPHKNIYNNNICEETKNNLNQNHVQNKY
DSEINIHNSNLENKNNSRNNILPLNINNVLNNYNNKMYIQNDYNINNNYAPKIKNDEKYV
CFFCSTSLFIHNKIRSFINWPKAECTLFLLQNEVIKPLEIKIIKSSYDLNNNYKFIKYDG
LINTHDQHTCFDNIPRNFVYIQNDSLNILCKNNTLLKIYKLQQKNKKIVDAMSFINSINK
CSLLY
```

B. FASTA sequence of DEAD/DEAH box helicase from isolate 3D7 proteome database. There is no putative prenylation motif at the C-terminus.

```
>tr|Q8I511|Q8I511_PLAF7 ATP-dependent RNA helicase DBP9, putative
OS=Plasmodium falciparum (isolate 3D7) GN=PF3D7_1241800 PE=4 SV=2
MNDAQIKDMMNDEEEKEEIEIKENINDKINEIDDNDDNDDGDDDGDDDDEDEDEDSDDND
DEDNDEDEDNDDNDDNDDSDNNDDDHNDVKKSSKNKKKNNEEMFDRSNHFDNFDNIMLDV
RLRKALLYLFKYQHPTIIQKMSICKILNGHDVIISSKTGSGKTMAYLIPVVHNLIKFNLN
EKDHLKFFYKCIIICPTEELCLQIYDVTKKLCTYLKDIITVNHNVNNTFYEHPTILISTP
KDLCTHIIEKKKKNNLDILMNLKILILDEADVLHTQEFQSYLKTLTSYLPKKFNKKYQIV
MASATLKRNILEKTKLFLHNPIYVSHEQKNESSFEKKKNKTNNTNSVIMKREEAGKNNIH
DEGNEGKTKYQKFTGKAFYYVYKEELIKYIYLYNLIKIKIIPYKSIIFTTTIHDAYKIKI
FLTYLNVSSSILNPNHPILIRQNIISAFNNSKFHFLICPQYEKNNMKHVKGVLGSNKMDN
VDGGDDNDDDEDDGDYNDDDDNDDDDNDDDNDDDNDNDDDEDNDEDEDNDDDDGDDNDDD
NDDNNDDDNDNDDQTLNEPLSDNNSCRTYNSDDENEKTDDTTKLNEEKDFLYSRGLDFYD
VKCVVNFDMPSDSETFIHRIGRTCRLNNKGKCISFVNELNYGEKEFLQKLIEDKNICTMI
KKNIQYNIVEKYRYRVESTLNKCTNKKIKLFIQKEILYQSLKSKELKDFFNTNINEKRKI
NKIIKHFNKAVIPQKLIKDRNQSIFLNKSKVKNKIIQKNNTNNKNKNNNNNIKPFALKNN
NGLVITEQGYESQLTKEPEREVADPSKLPPLCGQRLRNYMYLKYIKGKKNKNGSNMNKSY
NSNNKKRKNNNKYNNRYNKYNKKGINKNHNKRNTNFNR
```

C. FASTA sequence of DEAD/DEAH box helicase from isolate HB3 proteome database. The putative prenylation motif CMLQ is present.

```
>tr|A0A0L7KBA8|A0A0L7KBA8_PLAFX Uncharacterized protein OS=Plasmodium
falciparum (isolate HB3) GN=PFHG_02136 PE=4 SV=1
MNDAQIKDMMNDEEEKEEIEIKENINDKINEIDDVDGGDDNDDNDDGDDDGDDDDEDEDE
DNDDNDDEDNDNDEDNDDDEDNDDDEDNDEDEDNDDNDDNDDSDNNDDDHNDVKKSSKNK
KKNNEEMFDRSNHFDNFDNIMLDVRLRKALLYLFKYQHPTIIQKMSICKILNGHDVIISS
KTGSGKTMAYLIPVVHNLIKFNLNEKDHLKFFYKCIIICPTEELCLQIYDVTKKLCTYLK
DIITVNHNVNNTFYEHPTILISTPKDLCTHIIEKKKKNNLDILMNLKILILDEADVLHTQ
EFQSYLKTLTSYLPKKFNKKYQIVMASATLKRNILEKTKLFLHNPIYVSHEQKNESSFEK
KKNKTNNTNSVIMKREEAGKNNIHDEGNEGKTKYQKFTGKAFYYVYKEELIKYIYLYNLI
KIKIIPYKSIIFTTTIHDAYKIKIFLTYLNVSSSILNPNHPILIRQNIISAFNNSKFHFL
ICPQYEKNNMKHVKGVLGSNKMDNVDGGDDNDDDEDDGDYNDDDDNDDDDNDDDNDDDDN
DDDEDNDEDEDNDEDEDNDDDDGDDNDDNNDDQTLNEPLSDNNSCRTYNSDDENEKTDDT
```

TKLNEEKDFLYSRGLDFYDVKCVVNFDMPSDSETFIHRIGRTCRLNNKGKCISFVNELNY
GEKEFLQKLIEDKNICTMIKKNIQYNIVEKYRYRVESTLNKCTNKKIKLFIQKEILYQSL
KSKELKDFFNTNINEKRKINKIIKHFNKAVIPQKLIKDRNQSIFLNKSKVKNKIIQKNNT
NNKNKNNNNNIKPFALKNNNGLVITEQGYESQLTKEPEREVADPSKLPPLCGQRLRNYMY
LKYIKGKKNKNGSNMNNSYNSNNKKRKNNNKYNNRYNKYNKKGINKNHNKRNTNFNRIQK
KIIFLLSLFCMLQ

D. FASTA sequence of DEAD/DEAH box helicase from isolate Camp/Malaysia proteome database. The putative prenylation motif CMLQ is present.

>tr|A0A024X4J9|A0A024X4J9_PLAFC Uncharacterized protein (Fragment)
OS=Plasmodium falciparum (isolate Camp / Malaysia) GN=PFMC_04004 PE=4
SV=1 DDDNDNDDQTLNEPLSDNNSCRTYNSDDENEKTDDTTKLNEEKDFLYSRGLDFYDVKCVV
NFDMPSDSETFIHRIGRTCRLNNKGKCISFVNELNYGEKEFLQKLIEDKNICTMIKKNIQ
YNIVEKYRYRVESTLNKCTNKKIKLFIQKEILYQSLKSKELKDFFNTNINEKRKINKIIK
HFNKAVIPQKLIKDRNQSIFLNKSKVKNKIIQKNNTNNKNKNNNNNIKPFALKNNNGLVI
TEQGYESQLTKEPEREVADPSKLPPLCGQRLRNYMYLKYIKGKKNKNGSNMNNSYNSNNK
KRKNNNKYNNRYNKYNKKGINKNHNKRNTNFNRIQKKIIFLLSLFCMLQ

Figure S2. Comparison of peptide sequences identified between *P. falciparum* and human Rab protein homologs. *P. falciparum* proteins contain peptides present in human homologs but with statistically higher probabilities, spectral counts, and percent coverages.

A. Comparison of peptide sequences identified between Pf Rab7 and human Rab7a.

C0H516_PLAF7 (100%), 23,788.5 Da
PfRab7, GTPase OS=Plasmodium falciparum (isolate 3D7) GN=Rab7 PE=3 SV=1
7 exclusive unique peptides, 8 exclusive unique spectra, 25 total spectra, 121/206 amino acids (59% coverage)

MSNKKRTILK **VIILGDSGVG** **KTSLMNQYVN** **K**KFTNQYK**AT** **IGADFLTKET**
IVDNEQITMQ **IWDTAGQER**F QSLGVAFYRG ADCCVLVFDL TNYK**TYESLE**
SWKDEFLIQA **SPK**DPENFPF VIIGNKVDET NKRKVQSLKV LQWCK**SNNNI**
PYFETSAKNA **INVDQAFDEI** **AR**KAMK**QEHQ** **EEQIYLPETF** **ALNNQSEQK**M
YKSRCC

C9J8S3_HUMAN (99%), 18,026.8 Da
Ras-related protein Rab-7a OS=Homo sapiens GN=RAB7A PE=1 SV=1
0 exclusive unique peptides, 0 exclusive unique spectra, 10 total spectra, 43/160 amino acids (27% coverage)

MTSRKKVLLK **VIILGDSGVG** **KTSLMNQYVN** **KK**FSNQYK**AT** **IGADFLTK**EV
MVDDRLVT**MQ** **IWDTAGQER**F QSLGVAFYRG ADCCVLVATK RAQAWCYSKN
NIPYFETSAK EAINVEQAFQ TIARNALKQE TEVELYNEFP EPIKLDKNDR
AKASAESCSC

B. Comparison of peptide sequences identified between Pf and human Rab1B.

Rab1b, GTPase OS=Plasmodium falciparum (isolate 3D7) GN=Rab1b PE=4 SV=1
3 exclusive unique peptides, 4 exclusive unique spectra, 19 total spectra, 74/200 amino acids (37% coverage)

MNDSYDSLFK **ILLIGDSGVG** **K**SCLLLRFAD DTYTDS**YIST** **IGVDFK**IKTI
EIEDKIIK**LQ** **IWDTAGQER**F R**TITSSYYRG** **AQGIIIVYDV** **TDR**DSFNNVK
NWIIEIEKYA SEDVQKILIG NKIDLKNDRN VSYEEGKELA DSCNIQFLET
SAK**IAHNVEQ** **AFK**TMAYEIK NKSQHETINK GKTNINLNAR PIKDTKKKCC

A0A087WTI1_HUMAN (36%), 18,678.1 Da
Ras-related protein Rab-1B OS=Homo sapiens GN=RAB1B PE=1 SV=1
0 exclusive unique peptides, 0 exclusive unique spectra, 13 total spectra, 40/162 amino acids (25% coverage)

MNPEYDYLFK **LLLIGDSGVG** **K**SCLLLRFAD DTYTES**YIST** **IGVDFK**IRTI
ELDGKTIK**LQ** **IWDTAGQER**F R**TITSSYYR**G AHGIIVVYDV TDQESYANVK
QGCRRLTAMP ARTSISSWWA TRATSPPRRW WTTPQPRSLQ TLWASPSWRR
APRMPPMSSR RS

C. Comparison of peptide sequences identified between Pf and human Rab1A.

Q8I3W9_PLAF7 (100%), 23,860.6 Da
PfRab1a OS=Plasmodium falciparum (isolate 3D7) GN=Rab1a PE=3 SV=1
2 exclusive unique peptides, 2 exclusive unique spectra, 12 total spectra, 45/207 amino acids (22% coverage)

M T E N R **S R D Y D** **Y L Y K I I L I G D** **S G V G K** S C I L L R F S D D H F T E S Y I T T I G V D F R
F R T I K V D D K I V K **L Q I W D T A G** **Q E R** F R T I T S A Y Y R **G A D G I I I** **I Y D T T D R** N S F
L H I N D W M N E I N K Y T N E D T C K L L V G N K A D C K D D I E I T T M E G Q N K A K E L N I S
F I E T S A K D A T N V E L A F T M I T Q E L I K K K K K N F T S L K N N H A K L K L S T H D N S
P Q S F C S C

RAB1A_HUMAN (27%), 22,678.5 Da
Ras-related protein Rab-1A OS=Homo sapiens GN=RAB1A PE=1 SV=3
0 exclusive unique peptides, 0 exclusive unique spectra, 13 total spectra, 40/205 amino acids (20% coverage)

M S S M N P E Y D Y L F K **L L L I G D S** **G V G K** S C L L L R F A D D T Y T E S **Y** **I S T I G V D F K** I
R T I E L D G K T I K **L Q I W D T A G Q** **E R** F R **T I T S S Y** **Y R** G A H G I I V V Y D V T D Q E S F N
N V K Q W L Q E I D R Y A S E N V N K L L V G N K C D L T T K K V V D Y T T A K E F A D S L G I P F
L E T S A K N A T N V E Q S F M T M A A E I K K R M G P G A T A G G A E K S N V K I Q S T P V K Q S
G G G C C

D. Comparison of peptide sequences identified between Pf and human Rab5C.

Q8I274_PLAF7 (100%), 24,329.4 Da
Rab5c, GTPase OS=Plasmodium falciparum (isolate 3D7) GN=Rab5c PE=3 SV=1
7 exclusive unique peptides, 8 exclusive unique spectra, 18 total spectra, 91/214 amino acids (43% coverage)

M A Y Y L S N L N N N E K Y E T N Q S Y N S T K V F N S K **L** **V L L G D T S V G K** S C I V V R F A K N
E F Y E Y Q E S T I G A A F M T Q L I D I G E C T I K **F E I** **W D T A G Q E R** Y R **S L A P M Y Y R G A**
S A A V I V Y D I T **N K K** S F E G A K G W I H E L K **S V H S** **N D I I I A L A G N** **K** N D L E E H R A V
D R **E L A E S F A N** **S N N I L F I E T S** **A K T G Q N V N E L** **F L R** I A K K L P L H K K E Q E R C P A
I Q I N N T E E T K K K C C

RAB5C_HUMAN (47%), 23,482.7 Da
Ras-related protein Rab-5C OS=Homo sapiens GN=RAB5C PE=1 SV=2
0 exclusive unique peptides, 0 exclusive unique spectra, 6 total spectra, 11/216 amino acids (5% coverage)

M A G R G G A A R P N G P A A G N K I C Q F K L V L L G E S A V G K S S L V L R F V K G Q F H E Y Q
E S T I G A A F L T Q T V C L D D T T V K **F E I W D T A G Q** **E R** Y H S L A P M Y Y R G A Q A A I V V
Y D I T N T D T F A R A K N W V K E L Q R Q A S P N I V I A L A G N K A D L A S K R A V E F Q E A Q
A Y A D D N S L L F M E T S A K T A M N V N E I F M A I A K K L P K N E P Q N A T G A P G R N R G V
D L Q E N N P A S R S Q C C S N

MS Analysis Parameters

Parameter	Value	
	MS1 (Orbitrap)	*MS 2 (Ion trap)*
Orbitrap resolution	30000	-
Mass range	normal	-
CID collision energy (%)	-	35
Activation Q	-	0.25
Scan range (m/z)	300-1500	auto
AGC Target	500,000	5,000
Max. Injection time (ms)	50	25
Microscans	1	1
Data type	centroid	centroid

MS Filters:

Charge state: 2-7
    Exclude undetermined charge states
Dynamic exclusion duration (s): 90
    Mass tolerance: 0.01 m/z for both high and low
Intensity threshold: 500
